# Mining single-cell data for cell type–disease associations

**DOI:** 10.1093/nargab/lqae180

**Published:** 2024-12-18

**Authors:** Kevin G Chen, Kathryn O Farley, Timo Lassmann

**Affiliations:** Precision Health, The Kids Research Institute Australia, 15 Hospital Ave, Nedlands, 6009, WA, Australia; Precision Health, The Kids Research Institute Australia, 15 Hospital Ave, Nedlands, 6009, WA, Australia; Precision Health, The Kids Research Institute Australia, 15 Hospital Ave, Nedlands, 6009, WA, Australia

## Abstract

A robust understanding of the cellular mechanisms underlying diseases sets the foundation for the effective design of drugs and other interventions. The wealth of existing single-cell atlases offers the opportunity to uncover high-resolution information on expression patterns across various cell types and time points. To better understand the associations between cell types and diseases, we leveraged previously developed tools to construct a standardized analysis pipeline and systematically explored associations across four single-cell datasets, spanning a range of tissue types, cell types and developmental time periods. We utilized a set of existing tools to identify co-expression modules and temporal patterns per cell type and then investigated these modules for known disease and phenotype enrichments. Our pipeline reveals known and novel putative cell type–disease associations across all investigated datasets. In addition, we found that automatically discovered gene co-expression modules and temporal clusters are enriched for drug targets, suggesting that our analysis could be used to identify novel therapeutic targets.

## Graphical Abstract

**Graphical Abstract:**
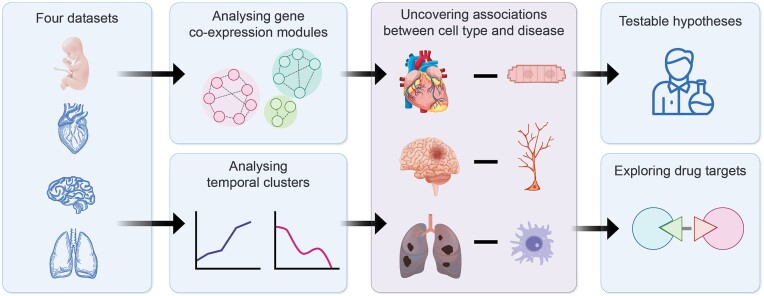

## Introduction

Understanding the biological mechanisms associated with diseases is essential for developing effective interventions and treatments. Diseases are often characterised by changes in cellular gene expression (1–3) and therefore, studying the transcriptomic profile of different cells can help identify genes and cellular populations relevant to disease pathogenesis (4,5). The development of single-cell RNA sequencing (scRNA-seq) technologies and associated single-cell atlases allows us to examine the transcriptomes of individual cells in unprecedented detail, enabling the exploration of cellular populations at a high resolution (6,7). scRNA-seq technologies have advanced our understanding of cell types and states within various human cellular systems (8–10), as well as the heterogeneous cell behaviour underlying diseases such as cancers (11–13).

Although the throughput and cost per cell for scRNA-seq have improved over time, the cost per sample remains high, preventing its widespread use (14). Additionally, technical considerations, such as maintaining RNA integrity during sample handling (15) or access to relevant tissue for sampling (16,17), can present further difficulties in studying diseases. These factors currently limit the range of diseases and cell types that can be studied using scRNA-seq technologies. To address these challenges, interrogating single-cell atlases of healthy individuals can enhance our understanding of the biology underlying diseases (18). For example, analysing expression patterns of genes implicated in a disease could help identify cell types relevant to that disease.

Prior research using single-cell data to explore disease mechanisms involved comparing cellular expression profiles in affected and unaffected individuals (19–21) and characterizing cells expressing known disease-relevant genes (22,23). In large-scale single-cell atlases, the latter approach is more common, as obtaining relevant cell types directly from patients is often not feasible. Techniques such as the one-sample z-test (24) and over-representation tests (25) have been applied in the past to demonstrate the relevance of specific cell types in various diseases (26–28). Other methods, such as Expression Weighted Cell Type Enrichment (EWCE) (29), leverage the resolution of single-cell data in identifying associations between phenotypic traits and cell types. The granularity afforded by single-cell data improves the specificity of these associations, and expanding this knowledge will enhance our understanding of cell type–disease associations. For example, previous studies have shown that analysing gene networks can be valuable in studying cell type–disease associations (30–32) by revealing a cell type’s involvement in disease and offering insights into functionally related genes. Similar studies have explored the temporal dynamics associated with disease (33,34). A comprehensive overview of cell type–disease associations, along with the co-expression and temporal modules relevant to disease, would contribute to a better understanding of the etiologies for many diseases.

Given the vast amount of single-cell data currently available, we sought to utilize these data to characterize associations between cell types and disease using a suite of existing tools. Specifically, we aimed to develop a standardised procedure to explore single-cell atlases, identifying cell types relevant to diseases. Our approach employs unsupervised methods to construct gene co-expression modules and clusters of genes sharing common temporal patterns. We then test whether these gene sets are enriched for known disease genes. We hypothesized that applying our pipeline systematically across multiple single-cell atlases would reveal biologically interesting associations between genes, cell types and diseases.

## Materials and methods

### Selection of datasets

We selected four scRNA-seq datasets to explore cell type to disease associations using our standardized analysis pipeline. These datasets were the Human Gene Expression During Development (Fetal Gene Atlas, accessed 9 August 2023) (35), the Heart Cell Atlas (accessed 13 September 2023) (36), the Developmental Cell Atlas of the Human Cerebellum (Fetal Cerebellar Atlas, accessed 2 October 2023) (37), and the *in vitro* and *in vivo* Development of the Human Airway (Fetal Lung Atlas, accessed 30 September 2023) (38). Datasets were selected to capture a variety of sequencing protocols, organ and cell types, and dataset sizes. Cells in these datasets had existing cell-type annotations and age information. For each dataset, we downloaded the raw counts matrix and associated metadata.

### Expression weighted cell type enrichment analysis

The EWCE package (v1.9.3) (29,39) was employed to act as a baseline method to identify disease and cell type associations across our datasets. The raw gene count matrices were first normalised and scaled using the sctransform (v0.3.5) framework (40), removing genes not detected in any cells. All other parameters were left as default. Subsequently, genes detected in at least 5% of cells in any cell type were included in the remainder of the EWCE analysis. The normalised count matrices were pseudo bulked via the generate_celltype_data function in EWCE, which was used to determine mean expression and gene specificity for each cell type described in the metadata. These objects were utilised to enrich cell types for gene lists linked to Human Phenotype Ontology (HPO) terms (41) via the MultiEWCE::gen_results function, which parallelised the enrichment analysis. We applied 10 000 bootstrap replicates to produce the results. Associations with a *q*-value <0.05 were considered significant.

### Gene co-expression module analysis

We utilised the hdWGCNA package (42) to generate and analyse co-expression modules from the single-cell data. Our objective was to expand upon the EWCE findings through exploring disease associations mediated by gene co-expression modules by both identifying enriched phenotypes and cell types these modules were active in.

Raw counts matrices were first processed via Seurat (v4.3.0) (43). Specifically, this entailed filtering to exclude any genes which were not detected in any cell, as well as removing cells with no detected genes. The data were then normalised, with the normalised data used to identify variable genes, using default settings throughout. Variable genes were used for data scaling and linear dimensionality reduction, also employing default parameters. Subsequently, a non-linear dimensionality reduction using UMAP with 30 dimensions was performed before cells were grouped into clusters via FindNeighbors and FindClusters with a resolution of 1.5.

For each dataset, the processed Seurat object served as input into hdWGCNA (v0.2.21) (42) to determine gene co-expression modules across all cells. Genes were only included if they were detected in 5% of cells in any cell type. Cell type annotations and the biological sample origin acted as grouping variables when applying the MetacellsByGroups function to construct metacells. For this function, the maximum number of shared cells was set to 30 and the *k*-parameter was set to 50 for all our analyses to accommodate the large datasets. Following the authors’ guidelines, we used default settings for normalisation via NormalizeMetacells and constructed the co-expression network using ConstructNetwork.

A one-sided two-sample *t*-test was used to test the association between gene co-expression modules and cell types. This test compared the mean Seurat module score, determined by AddModuleScore, for each cell type against the mean module score for all other cells across all cell type-module pairs. This test was conducted for all cell type-module pairs in a dataset, with a Benjamini-Hochberg correction performed via stats::p.adjust. Pairs with a Benjamini-Hochberg corrected *P*-value < 0.05 were called as a significant association. Subsequently, clusterProfiler (v4.8.3) (44) was used to enrich each co-expression module for HPO terms against a background of all genes included in hdWGCNA, utilising the enricher function in compareCluster and specifying the ‘BH’ parameter for pAdjustMethod. Any module to HPO term enrichment with a Benjamini-Hochberg corrected *P*-value < 0.05 and at least 10 contributing genes was retained.

### Temporal clustering analysis

In addition to the gene co-expression module analysis, we identified temporally related groups of genes that were enriched for disease. This analysis was designed to identify time course patterns and specific timepoints that may be relevant to disease pathogenesis. To group genes into shared temporal patterns in individual cell types, the raw counts matrix was first processed using the sctransform framework as outlined in our preparation for EWCE. For each cell type, we first identified genes that were detected in at least 5% of the cells. Then, for these selected genes, we calculated their average expression at each defined time point using Seurat’s AverageExpression function, with time points based on each cell’s sampling time according to the metadata. The average expression matrix was then used as input for TCseq (v1.24.3) (https://bioconductor.org/packages/TCseq), which clustered genes into eight groups representing different temporal patterns. Finally, each time cluster was enriched for HPO terms against a background of the selected genes. The datasets stored age information heterogeneously, including formats such as days, weeks or years. Due to this variability, we defined significant associations more flexibly, focusing on those with 10 or more contributing genes and a Benjamini-Hochberg corrected *P*-value ≤ 0.1.

### Validation of enrichment methodology

We validated enrichments captured by our gene co-expression methodology. To do so, we employed 5-fold cross-validation approach using the Heart Cell Atlas dataset. We applied our enrichment methodology to the training dataset to identify significant cell type–HPO term associations. In the test group, we assessed these enrichments using AUCell (v1.26.0) (https://bioconductor.org/packages/release/bioc/html/AUCell.html) to calculate the activity of HPO gene sets in each cell through an area under the curve (AUC) score. For each HPO gene set, we identified the associated cell types from the training dataset. We then compared the distribution of AUC scores between associated and non-associated cells using a one-sided Wilcoxon rank-sum test, using the alternative hypothesis that associated cells would have higher AUC scores. To control for the false discovery rate due to multiple comparisons, we performed a Benjamini-Hochberg multiple hypothesis correction on the *P*-values generated from the Wilcoxon rank-sum test. We recorded the proportion of enrichments in the training dataset that were validated in the test group, investigating consistency when adjusted *P*-value thresholds of 0.05 and 0.01 were applied for both our enrichment methodology and validation tests.

### Identifying drug targets in gene co-expression modules and temporal clusters

To illustrate the utility of our integrated pipeline, we investigated whether gene co-expression modules and temporal clusters could reveal drug targets for phenotypes that were identified as enriched in these modules and clusters. We used the OpenTargets platform (45) to identify drug targets for phenotypes defined in the HPO, comprising 1135 different phenotypes. We performed a hypergeometric test using the phyper() R function to determine if any co-expression module was over-represented in drug targets for HPO terms defined in OpenTargets. Within each dataset, this hypergeometric test was performed independently for all possible HPO term and co-expression module or temporal cluster combination, and hence Benjamini-Hochberg corrections were performed. We defined any association with an adjusted *P*-value ≤ 0.05 as a co-expression module or temporal clustering being over-represented for known drug targets for a certain phenotype.

## Results

### Validation and novel insights from EWCE analysis across multiple single-cell atlases

We assessed the potential of EWCE to uncover cell type–disease associations across four single-cell datasets. The Fetal Gene Atlas, previously analysed by the authors of EWCE, served as a control to ensure we could replicate their findings. The other three datasets had not been analysed explicitly for cell type–disease associations via EWCE. For the Heart Cell Atlas and Fetal Cerebellar Atlas, we compared our findings to those reported in their respective publications. Since no cell type–disease associations were reported for the Fetal Lung Atlas in its publication, we compared our findings to those previously reported in literature.

**Figure 1.**
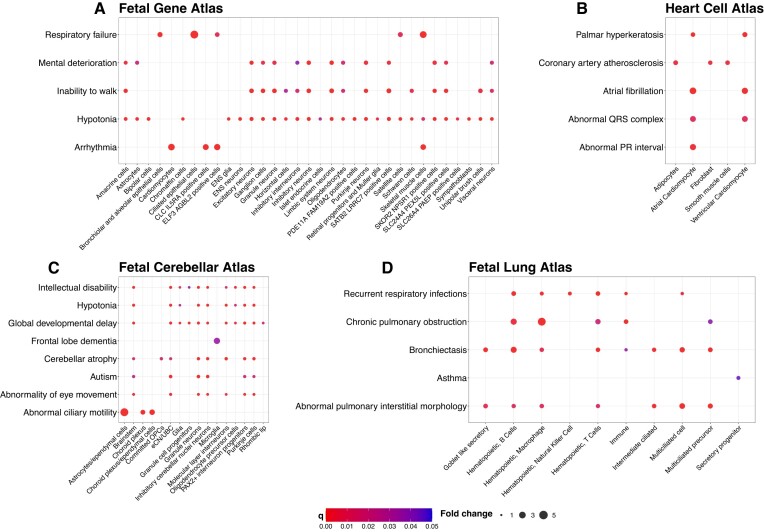
Cell type-HPO term associations identified by EWCE. Dotplots depict HPO terms which were identified to be enriched in particular cell types. The presence of a dot indicates a significant association. (**A**) Fetal Gene Atlas, (**B**) Heart Cell Atlas, (**C**) Fetal Cerebellar Atlas (eCN/UBC = Excitatory cerebellar nuclei neurons/Unipolar brush cells) and (**D**) Fetal Lung Atlas. For each dot-plot, *x*-axis represents the cell type and the *y*-axis represents the HPO term. Only associations where *q* < 0.05 are shown.

In the Fetal Gene Atlas, our results largely aligned with those reported by Murphy *et al.* (Supplementary Table S1). Consistent associations included those between ‘Respiratory failure’ and bronchiolar and alveolar epithelial cells, ciliated epithelial cells and skeletal muscle cells; ‘Mental deterioration’ and central nervous system neurons and amacrine and ganglion cells; ‘Arrhythmia’ and cardiomyocytes; ‘Hypotonia’ and various neurons and glia including inhibitory and excitatory neurons as well as ENS glia and astrocytes; and ‘Inability to walk’ and excitatory neurons and Schwann cells (Figure 1A). However, inconsistencies arose in enrichments for ‘Recurrent Neisserial infections’. While Murphy *et al.* reported a significant association for both hepatoblasts and AFP/ALP + cells, we were unable to capture this enrichment (*q*-value = 0.09 for both cell types). Upon further inspection, differences in filtering compared to Murphy *et al.* resulted in the genes C6 and C7, encoding complement proteins, being retained in our analysis. Enrichments from EWCE are driven by genes displaying cell type-specific expression. Both C6 and C7 did not display high specificity in their expression within hepatoblasts and AFP/ALB + cells, and as such, retention of these genes contributed to non-significant ‘Recurrent Neisserial infections’ associations.

Next, we compared EWCE enrichments in the Heart Cell Atlas (Supplementary Table S2) to disease associations reported by Litviňuková *et al.* (36). In brief, Litviňuková *et al.* performed GWAS enrichment analyses for each cell type using MAGMA (46) with a selection of GWAS traits. We found both atrial and ventricular cardiomyocytes to be enriched for ‘Atrial fibrillation’ and ‘Abnormal PR interval’, consistent with the findings of Litviňuková *et al.* We then examined enrichments for ‘Coronary artery atherosclerosis’ and, in agreement with Litviňuková *et al.*, identified associations with smooth muscle cells and fibroblasts. We also identified an association with adipocytes, which appeared plausible given adipocytes have been associated with coronary artery disease (47). We next compared our EWCE enrichments for ‘Prolonged QRS duration’ to the association between neurons and QRS duration reported by Litviňuková *et al.* We instead found enrichments for both atrial and ventricular cardiomyocytes. Given that the QRS complex is responsible for the initiation of ventricular contraction, the associations we found were reasonable.

In extending our findings beyond comparisons against Litviňuková et al., we noted that atrial and ventricular cardiomyocytes were both enriched for terms relating to skin abnormalities, such as ‘Palmar hyperkeratosis’ (Figure 1B). Associations between cardiomyopathies and skin disease have been documented, for example, in Naxos disease, where these phenotypes are mediated through mutations in the plakoglobin-encoding *Jup* in cardiomyocytes and keratinocytes, respectively (48,49).

We similarly assessed the EWCE enrichments in the Fetal Cerebellar Atlas (Supplementary Table S3), comparing them against the cell types identified by Aldinger *et al.* (37) as being enriched for mutations causing various diseases via a one-sample *z*-test. Consistent with Aldinger *et al.*, we identified numerous cell types enriched for the HPO term ‘Autism’ (Figure 1C). Aldinger *et al.* also investigated cell types relevant to structural cerebellar malformations. In agreement with their findings, we captured an association between ‘Cerebellar atrophy’, a type of structural cerebellar malformation, and Purkinje cells. Also consistent with Aldinger *et al.*, we identified ‘Frontal lobe dementia’, a characteristic manifestation of Alzheimer’s disease, to be associated with microglia.

We also compared our results to the authors’ findings on Joubert syndrome (JS). While they did not identify significant associations with any cell type, we identified cell types that plausibly contribute to disease. We assessed enrichments for symptoms associated with JS, specifically ‘Abnormality of eye movement’, ‘Hypotonia’ and ‘Global developmental delay’, and found excitatory cerebellar nuclei neurons, unipolar brush cells, rhombic lip and Purkinje cells to be enriched for all three phenotypes (Figure 1C). Existing literature has indicated the involvement of cerebellar defects in JS (50,51); however, the role of specific cell types is not yet well characterised. These findings provide insights into cell types that may be relevant to disease onset and progression. To further investigate cell types relevant to JS, we considered its classification as a primary ciliopathy (52) and sought to identify cell types enriched for ‘Abnormal ciliary motility’. We found this term to be enriched in ependymal cells and cells in the choroid plexus. These are both well-characterised ciliated cells (53,54), and it is possible dysfunction in the cilia of these cells contributes to onset of JS.

Lastly, we investigated the Fetal Lung Atlas (Supplementary Table S4) and examined enrichments for phenotypes associated with lung diseases reported in the literature. Initially, we explored cystic fibrosis and identified ‘Bronchiectasis’ and ‘Recurrent bacterial infections’ as significantly enriched in various immune and ciliated cells (Figure 1D). This finding aligns with the known immune dysregulation and impaired mucociliary clearance associated with these phenotypes (55). We additionally found that ‘Elevated sweat chloride’, a hallmark of cystic fibrosis, was enriched in club-like and goblet-like secretory cells, as well as secretory progenitor cells (Figure 1D). Although lung secretory cells are not known to contribute to sweat homeostasis, it is plausible these cells have similar expression profiles to secretory cells of the sweat gland, which would explain the observed association between lung secretory cells and elevated sweat chloride. Our analysis of chronic obstructive pulmonary disease (COPD) revealed an enrichment between ‘chronic pulmonary obstruction’ with multiple immune and ciliated cells (Figure 1D). This was reasonable given these cells can drive the inflammation associated with COPD (56). We then checked enrichments for ‘asthma’, which we found to be in secretory progenitors and goblet-like secretory cells. This was consistent with the role of these cells in abnormal mucin production, which is a known contributing factor to asthma (57). Finally, we extended our analysis to interstitial lung diseases and found ‘Abnormal pulmonary interstitial morphology’ to be enriched across lymphocytes, macrophages, Goblet-like secretory cells and ciliated cells (Figure 1D). Given the immune dysregulation and alveolar surfactant issues associated with some interstitial lung diseases (58), the immune functions of lymphocytes, macrophages and ciliated cells, alongside the surfactant production role of secretory cells, collectively support this putative association.

In summary, we were able to recapitulate results from Murphy *et al.* using the Fetal Gene Atlas. We additionally demonstrated that EWCE captures previously reported, as well as novel, cell type–disease associations. These results demonstrate how the approach can be used to generate new hypotheses and directions for further exploration.

### Linking cell types to diseases using gene co-expression modules

Single-cell data allow us to construct high-quality gene co-expression networks. Here, we investigate whether the presence of co-expression modules in particular cell types is linked to specific phenotypes. Co-expression modules within a dataset were enriched for HPO terms, and we subsequently identified cell types where each co-expression module was highly active to infer associations between cell types and diseases. In our discussion, we refer to each co-expression module by colour.

**Figure 2.**
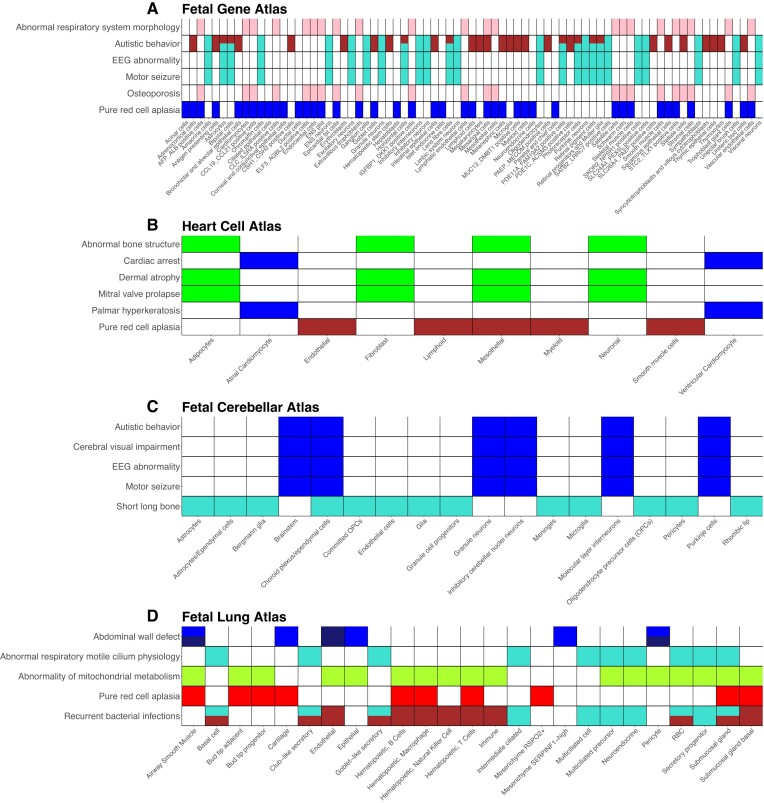
Gene co-expression modules link cell-types to diseases. Presence-absence matrices of selected significant terms when performing enrichment analysis using co-expression modules, where a coloured cell indicates the presence of a significant association. (**A**) Fetal Gene Atlas, (**B**) Heart Cell Atlas, (**C**) Fetal Cerebellar Atlas and (**D**) Fetal Lung Atlas. Colour represents the co-expression module for which the HPO term was significant, with multiple colours representing significant enrichment in multiple modules.

All datasets included a co-expression module enriched for phenotypes associated with ribosomal genes. This was particularly evident in the Fetal Gene Atlas, Heart Cell Atlas and Fetal Lung Atlas, each of which contained a module predominantly enriched for phenotypes such as ‘Pure red cell aplasia’, which are associated with many ribosomal genes (Figure 2A, B and D). These enrichments comprised 92.6%, 79.4% and 100% of HPO term enrichments of these datasets’ ribosomal module, respectively. We also identified a similar module (turquoise) in the Fetal Cerebellar Atlas, though only 64% of enrichments in this module were related to ribosomal genes, such as ‘Short long bone’ (Figure 2C). Ribosomal RNA (rRNA) represents a known challenge in scRNA-seq, as rRNA makes up 80–90% of RNA content in eukaryotic cells (59) and is often not of interest. As such, these modules likely arise as a consequence of the experimental procedure and we excluded the corresponding modules from our discussion below.

To test the robustness of the results, we performed a cross-validation approach as described in the methods section. Across the folds we were able to re-discover between 91.5% and 94.9% of the predicted enrichments in the test data (Benjamini-Hochberg adjusted *P*-value threshold of 0.05 as determined by Wilcoxon rank-sum test). When repeating the analysis with a more stringent threshold of 0.01, we found validation rates ranged from 96.5% to 98.7%, matching the expected error rates. The consistency between expected and actual results demonstrates the robustness of our approach.

Eight co-expression modules were generated for the Fetal Gene Atlas. One of these modules which was enriched for multiple neural HPO terms such as ‘Autistic behaviour’, ‘EEG abnormality’ and ‘Motor seizure’ (Figure 2A, turquoise). We found this module to be associated with multiple neural cell types, including Schwann cells and excitatory neurons. We also found ‘Autistic behaviour’ to be linked to many cell types including astrocytes, bipolar cells, hematopoietic stem cells and ureteric bud cells through the brown module (Figure 2A). The pink module was associated with a broad range of cell types and a diverse set of HPO terms including phenotypes relating to bones and joints such as ‘osteoporosis’, as well as those relating to morphology such as ‘Abnormal respiratory system morphology’ (Figure 2A).

Our analysis on the Heart Cell Atlas revealed eight co-expression modules, two of which were enriched for HPO terms related to cardiac function. The green co-expression module, associated with fibroblasts, adipocytes, mesothelial cells and neuronal cells, was enriched for skin and bone phenotypes, including ‘Dermal atrophy’ and ‘Abnormal bone structure’, in addition to terms relating to cardiac physiology, such as ‘Mitral valve prolapse’ (Figure 2B). The blue co-expression module was enriched for 161 HPO terms, 68 of which were descendants of ‘Abnormality of the cardiovascular system’ such as ‘Cardiac arrest’, and an additional 49 were descendants of ‘Abnormality of the musculature’. In this module, we also noted skin enrichments including ‘Palmar hyperkeratosis’. Genes in this module were most highly expressed in both atrial and ventricular cardiomyocytes, supporting the association that was found using EWCE (Figure 2B).

We generated ten co-expression modules for the Fetal Cerebellar Atlas. In enriching these co-expression modules for HPO terms, we observed the blue module to be enriched for terms such as ‘Autistic behaviour’, ‘Cerebral visual impairment’, ‘EEG abnormality’ and ‘Motor seizure’. We noted that cell types associated for this module included cell types such as Purkinje Cells, inhibitory cerebellar neurons and granule neurons (Figure 2C), which were cell types associated to ‘Abnormality of eye movement’ using EWCE. In addition to these cell types, the blue module was also active in brainstem, choroid plexus/ependymal cells and molecular layer interneurons, supporting the finding from EWCE that autism, when viewed from the perspective of gene expression analysis, is associated with many cell types (Figure 2C).

Lastly, we performed enrichment analysis on co-expression modules generated using data from the Fetal Lung Atlas, identifying 19 co-expression modules. The turquoise co-expression module, active in secretory and ciliated cell types, which was enriched for phenotypes such as ‘Abnormal respiratory motile cilium physiology’ and ‘Recurrent bacterial infections’ (Figure 2D). Additionally, we found associations between airway smooth muscle cells, endothelial cells and pericytes, with ‘Abdominal wall defect’, mediated by an enrichment in the midnight-blue module (Figure 2D). In addition to these, we noted the green-yellow module to be enriched for terms related to ‘Abnormality of mitochondrial metabolism’. This module was active in 17 different cell types, pointing to involvement of a broad range of cell types.

The genes making up co-expression modules are included in supplementary Supplementary Table S5, and full enrichment results are included in Supplementary Tables S6–S9. Taken together, these findings illustrate how we can use unsupervised machine learning techniques to mine public single-cell data for gene modules and examine their putative cell type–disease associations. Our approach provides insight into both the co-expression modules relevant to a disease but also the cell types in which these gene modules are active. Taken together, our findings evidence the cell type-specific nature of some diseases, as well as the broad, systemic relevance of other disease.

### Disease genes share cell type specific temporal gene expression patterns

Given that the datasets we analysed included timepoints associated with each cell, we aimed to determine whether genes exhibiting common temporal expression patterns within a cell type were linked to diseases.

As with the gene co-expression analyses, enrichments due to ribosomal genes were prevalent across all datasets: Fetal Gene Atlas (73.2% associated with ribosomal genes), Heart Cell Atlas (54.8%), Fetal Cerebellar Atlas (41.2%) and the Fetal Lung Atlas (48.3%). As these are unlikely to represent biological signals, our subsequent discussion of results focusses on non-ribosomal enrichments.

**Figure 3.**
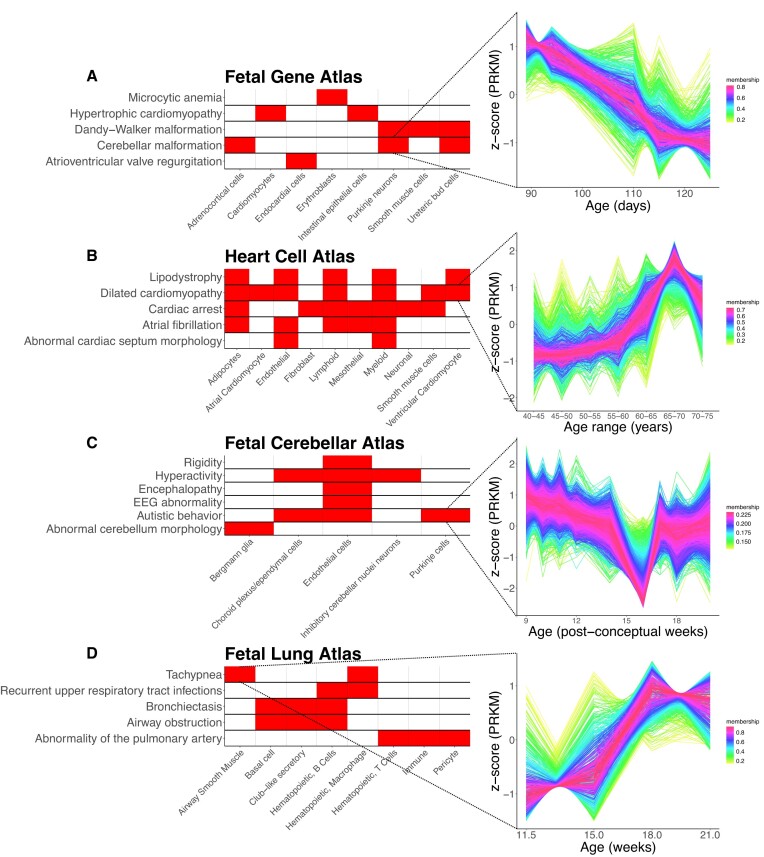
Genes sharing temporal gene expression patterns are enriched for HPO phenotypes. Enrichments identified when performing temporal clustering on the (**A**) Fetal Gene Atlas, (**B**) Heart Cell Atlas, (**C**) Fetal Cerebellar Atlas and (**D**) Fetal Lung Atlas. Left figure is a presence–absence matrix of selected enrichments, with shading indicating cell type–HPO term associations which were identified as significant (adjusted *P*. value < 0.1). For each dataset, one association is expanded, as denoted by the dotted lines, to show the temporal pattern (right) containing the enrichment. Temporal unit in the time-course plots was dependent on the temporal unit recorded in each dataset.

We identified meaningful enrichments in temporal clusters generated for the Fetal Gene Atlas. This included an association between erythrocyte deformities such as ‘Microcytic anemia’, with erythroblasts, with the genes driving this enrichment spiking at various recorded timepoints (Figure 3A). We also found ‘Focal aware seizure’ to be enriched in inhibitory neurons, with the associated genes being most highly expressed before 110 days (Figure 3A). We were also able to identify associations between Purkinje neurons and neural abnormalities such as ‘Cerebellar malformation’ and ‘Dandy-Walker malformation’, enriched in a gene cluster exhibiting high initial expression followed by a gradual decline (Figure 3A). An enrichment for ‘Cardiomyopathy’ was noted in cardiomyocytes, with genes most highly expressed at 94 and 113 days, as well as an enrichment for ‘Atrioventricular valve regurgitation’ in endocardial cells, in which genes were highly expression at 94 days prior to a gradual decrease.

We subsequently examined enrichments in the Heart Cell Atlas. We noted that many cell-type disease associations were linked to genes upregulated later in life. For example, ‘Cardiac arrest’ and ‘Dilated cardiomyopathy’ were enriched in seven and five different cell types respectively, with genes most highly expressed between the ages of 65–75 years (Figure 3B). Similarly, significant enrichments for ‘Lipodystrophy’ were noted in five difference cell types, all mediated in temporal clusters characterised by high expression between 60 and 75 years. The majority of associations with ‘Atrial fibrillation’ involved genes peaking in expression after 60 years, which held true for associations with adipocytes, endothelial, lymphoid, mesothelial and myeloid cells. Finally, associations between ‘Abnormal cardiac septum morphology’ and both endothelial and myeloid cells were identified. In these cases, genes mediating this enrichment were highly expressed between two distinct age ranges: 40–55 years and 60–65 years.

In enriching temporal clusters in the Fetal Cerebellar Atlas, we noted endothelial cells were associated with a range of neural phenotypes, such as ‘Rigidity’, ‘Encephalopathy’, ‘EEG abnormality’ and ‘Hyperactivity’ driven by genes exhibiting constant expression over time barring a spike at 18 weeks. ‘Hyperactivity’ was also enriched in inhibitory cerebellar nuclei neurons and choroid plexus/ependymal cells, where expression was against constant except for a notable decrease at 16 and 18 weeks, respectively. We additionally noted an association between Purkinje cells and ‘Autistic behaviour’, in which the temporal cluster was characterised by a slight decrease over time aside from a sudden decrease at 16 weeks. An association between Bergmann glia and ‘Abnormal cerebellum morphology’ was characterised by genes again slightly decreasing in expression over time with a dip at 16 weeks.

Finally, we assessed enrichments in the Fetal Lung Atlas. We identified both B cells and basal cells to be enriched for ‘Bronchiectasis’ (Figure 3D) consistent with the EWCE results. We identified additional associations with basal cells, club-like secretory cells and RSPO2 + mesenchymal cells. Except for club-like secretory cells, bronchiectasis-associated genes were most highly expressed at 21 weeks in these cell types. A subset of these cell types, specifically basal cells, club-like secretory cells and B cells, were also enriched for ‘Airway obstruction’ (Figure 3D). We detected an enrichment for ‘Tachypnea’ in airway smooth muscle cells and macrophages. In both of these cell types, the genes associated with ‘Tachypnea’ were most highly expressed at 18 weeks. We additionally detected an enrichment for ‘Respiratory tract infection’ in red blood cells, B cells and macrophages (Figure 3D). This enrichment was mediated by genes exhibiting high expression at 21 weeks for B cells and red blood cells, and 11.5 weeks for macrophages. Finally, an enrichment for ‘Abnormal respiratory motile cilium’ was detected in multiciliated precursor cells, driven by genes with high expression at 18 weeks (Figure 3D).

The temporal patterns are shown in Supplementary Figures S1–S4, with associated enrichments in Supplementary Tables S10–S13. Overall, the temporal clustering method corroborated some enrichments found using both above methods. The temporal analysis augmented above findings by providing insights into time points in where disease-relevant genes were active. Further, the broad range of time scales within each dataset highlights the scalability of this approach to other single-cell datasets, regardless of the timepoints used.

### Drug targets are prevalent across gene co-expression modules

To determine if our enrichment analysis could be relevant to the discovery of drug candidates, we extracted known drug targets associated with specific HPO terms from the Open Targets database. We then analysed the distribution and representation of each phenotype’s drug targets in our identified gene co-expression modules and temporal clusters. The data underlying our findings for the gene co-expression module analyses can be found in Supplementary Tables S14–S17, and the data for temporal clusters can be found in Supplementary Tables S18–S21.

**Figure 4.**
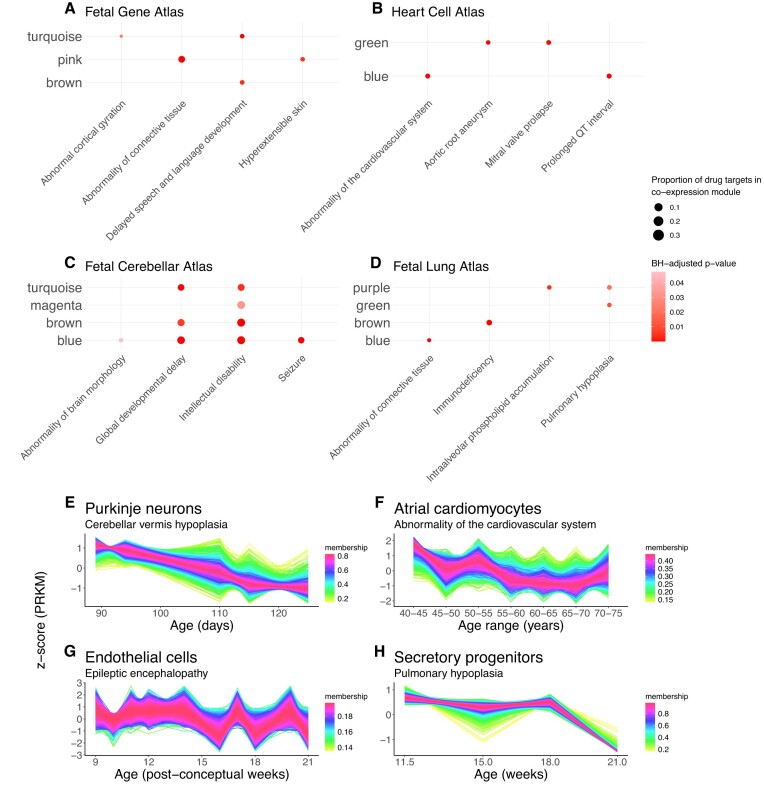
Exploration of known drug targets covered by gene co-expression modules and temporal clusters. (**A–D**) Dotplots of gene co-expression modules (*y*-axis) which were significantly enriched for known drug targets of selected phenotypes (*x*-axis). The presence of a dot indicates a significant overlap (adjusted *P*-value < 0.05), with a larger size indicating a greater overlap. (A) Fetal Gene Atlas, (B) Heart Cell Atlas, (C) Fetal Cerebellar Atlas and (D) Fetal Lung Atlas. (**E–H**) Examples of temporal clusters which were enriched for drug targets. One example was selected for each dataset: (E) Fetal Gene Atlas, (F) Heart Cell Atlas, (G) Fetal Cerebellar Atlas and (H) Fetal Lung Atlas. The cell type and HPO term are indicated in the title and subtitle, respectively, of each panel: for example, panel (E) demonstrates that, in the Fetal Gene Atlas, the specified temporal cluster was identified in Purkinje neurons, and drug targets for ‘Cerebellar vermis hypoplasia’ were overrepresented in that temporal cluster.

We began by evaluating drug target representation in the co-expression modules of the Fetal Gene Atlas. We observed instances were drug targets for certain HPO terms were significantly over-represented in specific co-expression modules. For example, drug targets associated with ‘Abnormal cortical gyration’ were overrepresented in the turquoise co-expression module, with ‘Delayed speech and language development’ drug targets overrepresented in both the turquoise and brown module. Known drug targets for ‘Hyperextensible skin’ and ‘Abnormality of connective tissue’ were enriched in the pink module (Figure 4A). These examples were notable given our above analyses indicated that the turquoise module was enriched for neural phenotypes, while the pink module was associated with skin and bone phenotypes. Our analysis suggests that these co-expression modules not only reflect associations between cell types and phenotypic traits but may also harbour other drug targets related to these phenotypes.

Our examination of the Heart Cell Atlas also revealed numerous cases where drug targets for cardiac phenotypes were overrepresented in our co-expression modules. For instance, drug targets for ‘Abnormality of the cardiovascular system’ and ‘Prolonged QT interval’ were overrepresented in the blue co-expression module (Figure 4B), which was active in cardiomyocytes and enriched for terms descendent from ‘Abnormality of the cardiovascular system’. Similarly, drug targets for ‘Aortic root aneurysm’ and ‘Mitral valve prolapse’ were enriched in the green co-expression module, active in fibroblasts, adipocytes, mesothelial cells and neurons.

In the Fetal Cerebellar Atlas, drug targets for several neural phenotypes were overrepresented in co-expression modules. Such phenotypes included ‘Abnormality of brain morphology’, ‘Global developmental delay’, ‘Intellectual disability’ and ‘Seizure’, in which drug targets were all overrepresented in the blue module (Figure 4C). Targets for ‘Global developmental delay’ were additionally overrepresented in the brown and turquoise modules, with known targets for ‘Intellectual disability’ also overrepresented in the brown, turquoise and magenta modules. Taking into consideration these phenotypes all had drug targets found in the blue module, targeting the function of cell types in which the blue module was active could be a relevant avenue for drug design.

Analysis of significant overlaps in the Fetal Lung Atlas revealed enrichment of drug targets for ‘Immunodeficiency’ with the brown co-expression module, which was active in immune cell types. Other notable overlaps involved overrepresentation of drug targets for ‘Intraalveolar phospholipid accumulation’ in the purple module and ‘Pulmonary hypoplasia’ in the purple and green co-expression modules (Figure 4D). Drug targets for ‘Abnormality of connective tissue’ were overrepresented in the blue module, which was active in cell types involved in structural integrity, including pericytes, airway smooth muscle cells, epithelial cells, and cartilage cells.

We additionally examined the overlap between known drug targets with the temporal clusters we generated. Identifying temporal patterns enriched for drug targets can provide insights into the timing of disease onset or progression, potentially informing strategies for treatment or prevention. Here, we highlight one exemplary example from each dataset.

We identified two examples where drug targets for a given HPO term were enriched in a temporal cluster that itself was enriched for similar HPO terms. In the Fetal Gene Atlas, targets for ‘Cerebellar vermis hypoplasia’ were enriched in a temporal cluster for Purkinje neurons characterized by high initial expression followed by a constant decrease over time (Figure 4E). This same temporal cluster was enriched for terms such as ‘Cerebellar malformation’. Similarly, in the Fetal Cerebellar Atlas, we noted a temporal cluster for endothelial cells enriched for known drug targets of ‘Epileptic encephalopathy’ (Figure 4G). This temporal cluster, which included genes with fluctuating expression, was enriched for ‘Encephalopathy’ as described above. These examples highlight cases where the expression patterns of relevant drug targets parallel the expression patterns of genes mediating disease.

In our analysis involving the Heart Cell Atlas, we identified a case where drug targets for ‘Abnormality of the cardiovascular system’ were overrepresented in a temporal cluster for Atrial cardiomyocytes (Figure 4F). Cardiac diseases in the Heart Cell Atlas tended to be enriched in temporal clusters characterized by high expression at later years (e.g. 60+ years). It was therefore notable that the temporal cluster highlighted here was instead characterised by high expression at comparatively earlier timepoints, with the highest expression at 40–45 years followed by a general decrease over time. While evidence supporting early treatment for heart diseases is lacking (60), it is recognized that risk factors such as blood pressure can manifest in young adulthood (61). As such, our findings here, where expression of druggable targets differs to that of genes mediating disease, appear plausible.

In the Fetal Lung Atlas, we were able to identify overrepresentation for drug targets of ‘Airway obstruction’ in a temporal cluster for secretory progenitor cells (Figure 4H). This temporal cluster was characterised by high initial expression followed by a steady decrease over time. This pattern paralleled the observed pattern for in the temporal cluster enriched for ‘Airway obstruction’ in club-like secretory cells, suggesting that targeting of the progenitor cells giving rise to these cells may be of importance as a drug target.

We were able identify instances where known drug targets were overrepresented in our co-expression modules and temporal clusters. Our analyses of co-expression modules demonstrated that co-expression modules which were themselves enriched for HPO terms were also often overrepresented for drug targets for similar HPO terms, indicating the possibility of identifying further drug targets in these co-expression modules. Analysis of the overlap between temporal clusters and known drug targets provided insight into time points where drug targets were active, helping clarify timepoints that are critical for effective activity of potential therapeutics.

## Discussion

We identified cell type–disease associations across all analysed datasets. Both EWCE and gene co-expression module analyses uncovered biologically plausible associations supported by existing literature, as well as novel associations. The co-expression module analysis additionally provided value by identifying gene networks that were mediating disease. These results were augmented by our temporal clustering analysis, which enabled identification of timepoints critical to disease.

As noted, there were differences in the enrichments we reported in EWCE compared to those of the original authors. For the Fetal Gene Atlas, discrepancies could be attributed to differences in the gene filtering step, with Murphy *et al.* removing genes with a mean count of <0.2 across all cell types. Various filtering steps can be viably applied, and our method was chosen for consistency with the default gene filtering strategy used in hdWGCNA. For the Heart Cell Atlas and Fetal Cerebellar Atlas, we attributed discrepancies to the different methods of generating enrichments. A large suite of tools is currently available designed to perform enrichments (29,44,62), each with strengths and limitations, and it is unsurprising that different methods can yield distinct findings. Nonetheless, the discrepancies in enrichment results reinforce the need for caution when interpreting *in silico* results.

The potential value of our results is illustrated by our findings on the rare diseases Naxos disease and JS. While there is no single HPO term representing either of these diseases, they can be characterized by a set of phenotypes that collectively describe the manifestation of disease. Treatment for Naxos disease is primarily based on knowledge from related, more thoroughly studied diseases (63), while treatment for JS currently focuses on symptom management (64). The lack of effective therapeutics highlights the understudied nature of these diseases, and our findings identify cell types, gene networks and temporal patterns that can serve as the focus of future studies to better understand the pathogenesis and potential treatment options for these diseases.

The gene co-expression module analyses demonstrated that co-expressed genes were often associated with relevant diseases. Our results demonstrate the cell-type specificity of some diseases, as well as the broad cellular involvement in others. The latter is exemplified by the enrichment of ‘Osteoporosis’ across various cell types, as identified in the Fetal Gene Atlas. Osteoporosis is characterized by low bone density and deterioration in bone architecture (65). However, it has been linked to factors involving other cellular systems such as fatty liver disease (66–68), justifying the associations with the liver-related epicardial fat cells and stellate cells, as well as cytokine signalling and other immune system pathways (69,70), supporting the identified associations with the various immune cells.

Our temporal clustering analysis provided insight into timepoints underlying disease, improving our understanding of the etiology of these diseases. We do note that any conclusion drawn about critical timepoints is limited to the age of samples represented in the dataset, and it is possible that biology underpinning the onset of disease instead lies in an age range beyond what each dataset contains. Nevertheless, the findings we highlight are supported by literature, with past studies showing some cerebellar malformations could be linked to alteration in Purkinje neuron firing at early stages (71,72), and cardiomyopathies known to manifest in late life (73,74), for example.

We note that some of our co-expression modules were overrepresented with drug targets for relevant phenotypes, providing evidence that further drug targets may exist within these specific co-expression modules. This information can be supplemented with enrichment analysis of drug targets in temporal clusters, which allow us to gain insight into time periods that may serve as therapeutic windows for drug design and administration. This demonstrates the value of a holistic approach in characterizing cell type-disease associations.

Our computational method identifies putative associations between cell types, gene co-expression, temporal modules and disease. These associations can inform what targeted assays and research directions can be performed to better understand the pathogenesis and potential treatment options for diseases more thoroughly. In addition, while we were able to manually identify literature supporting our enrichments, a systematic method to evaluate associations, for example automated methods integrating existing literature, would be a valuable future direction.

In conclusion, we were able to apply a uniform methodology to characterise cell type-disease associations in four different single-cell datasets. In addition to identifying cell types relevant to a disease, we were able to supplement these findings by determining gene networks and temporal patterns mediating these diseases. Our findings contribute to a deeper understanding of how disease-critical cell types contribute to manifestation of disease, as well as targeted avenues for how interventions can be developed to treat these diseases. We anticipate these benefits can be realized beyond the four datasets investigated in this study, as the method used can be applied to further single-cell datasets.

## Supplementary Material

lqae180_Supplemental_Files

## Data Availability

The data underlying this article, including code to reproduce the figures and analysis, are available in Zenodo, at https://dx.doi.org/10.5281/zenodo.11227998. The datasets were derived from sources in the public domain: Fetal Gene Atlas (https://descartes.brotmanbaty.org), Heart Cell Atlas (https://www.heartcellatlas.org), Fetal Cerebellar Atlas (https://cells.ucsc.edu/?ds=cbl-dev) and the Fetal Lung Atlas (https://cells.ucsc.edu/?ds=fetal-lung).
