# Supplementary material for: Mining single-cell data for cell type–disease associations
Source: NAR Genom Bioinform. 2024 Dec 18;6(4):lqae180. doi: 10.1093/nargab/lqae180 (PMC11655289; doi:10.1093/nargab/lqae180)

Adipocytes time clusters

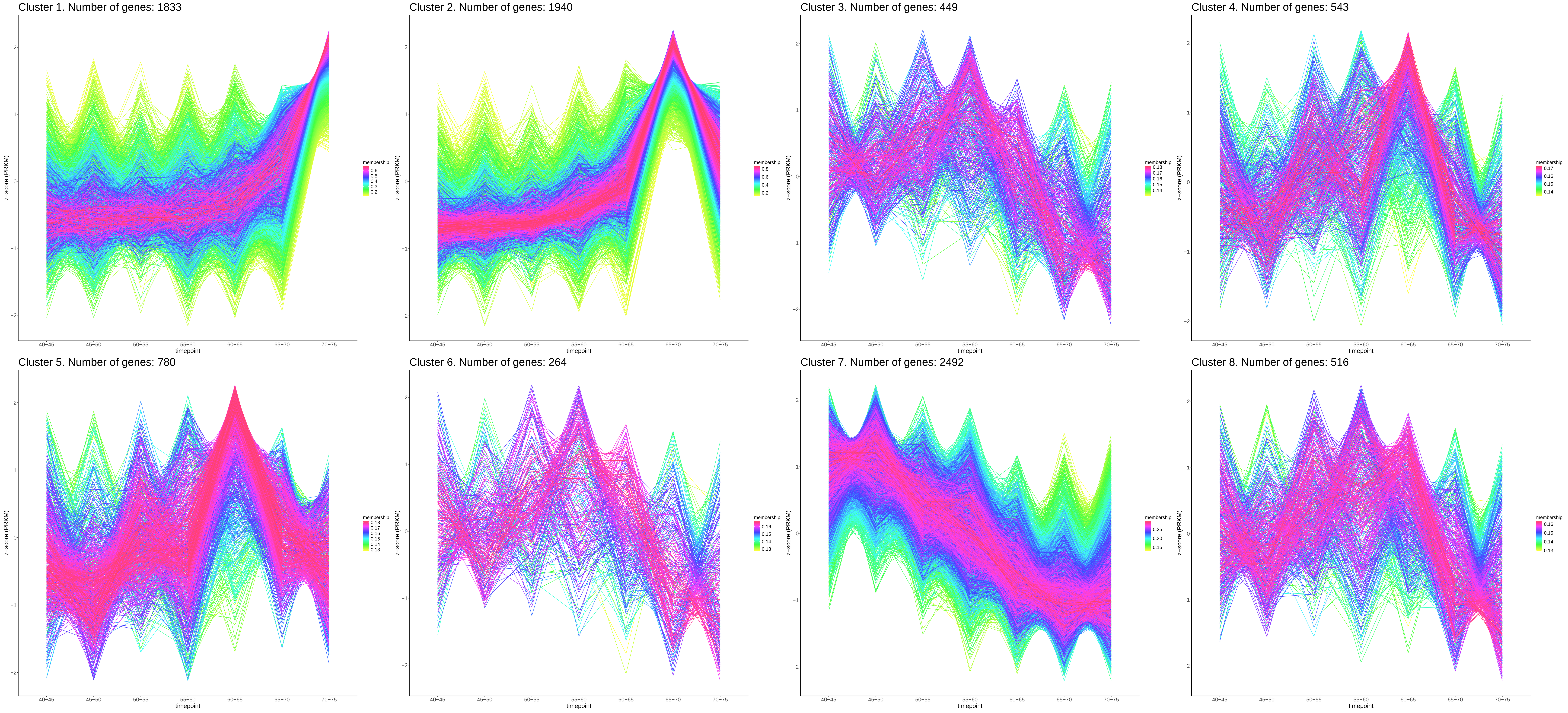

Atrial_Cardiomyocyte time clusters

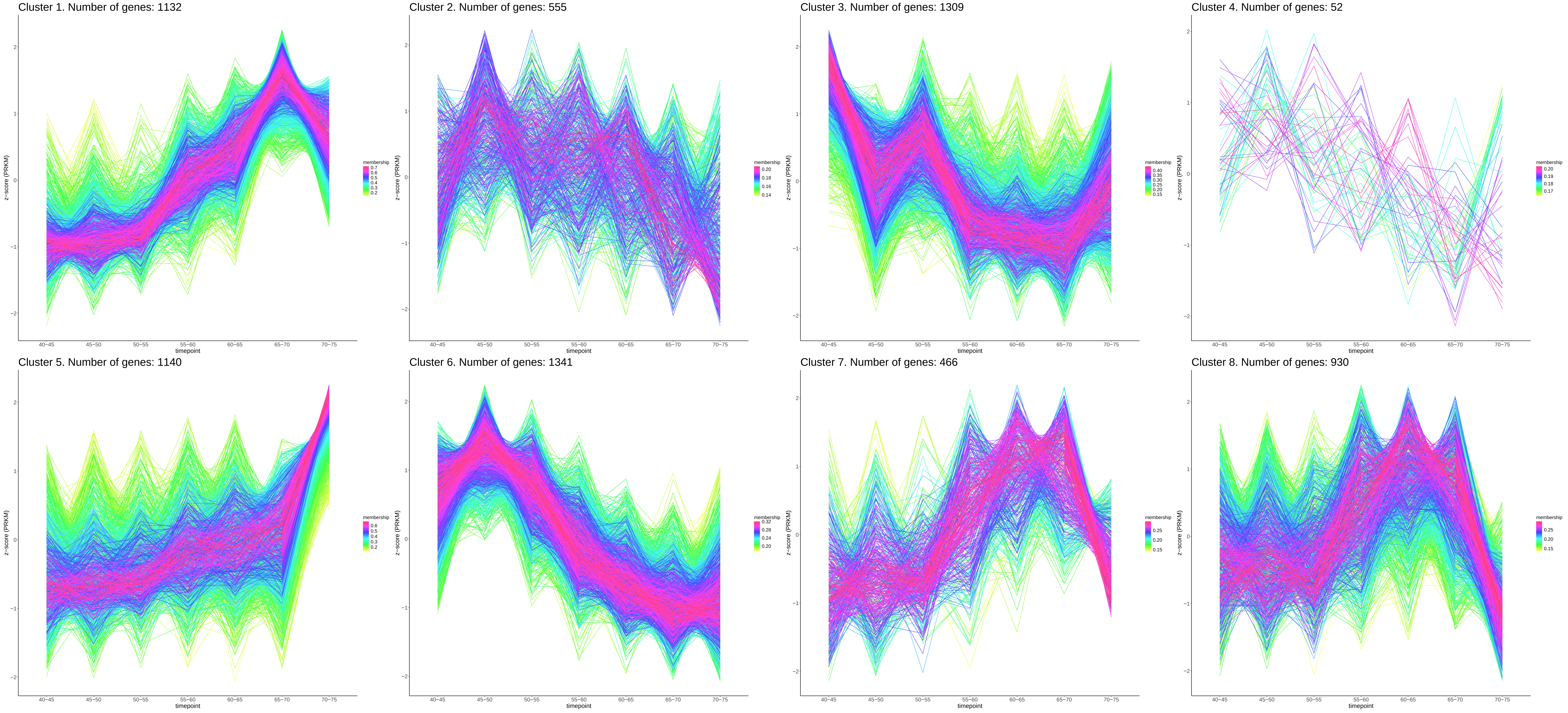

Endothelial time clusters

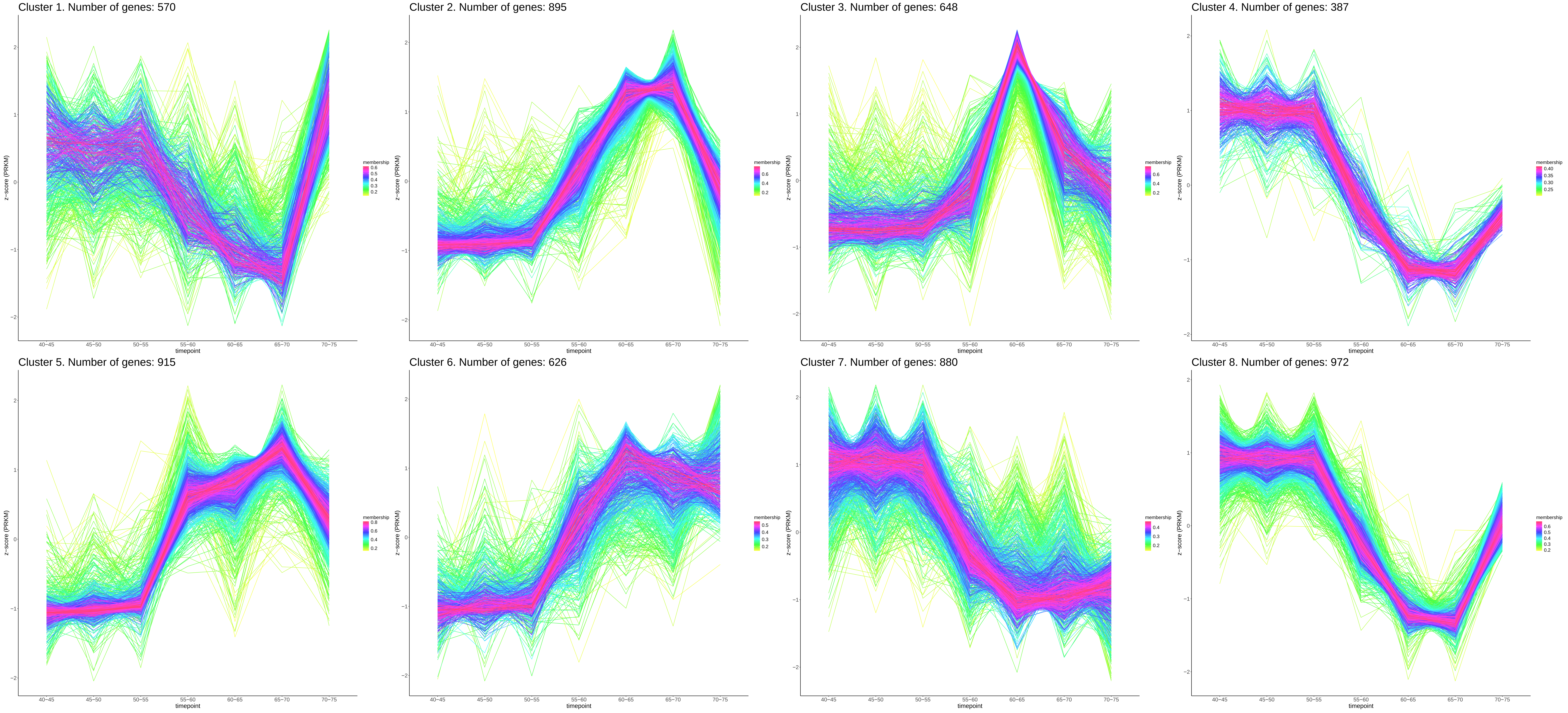

Fibroblast time clusters

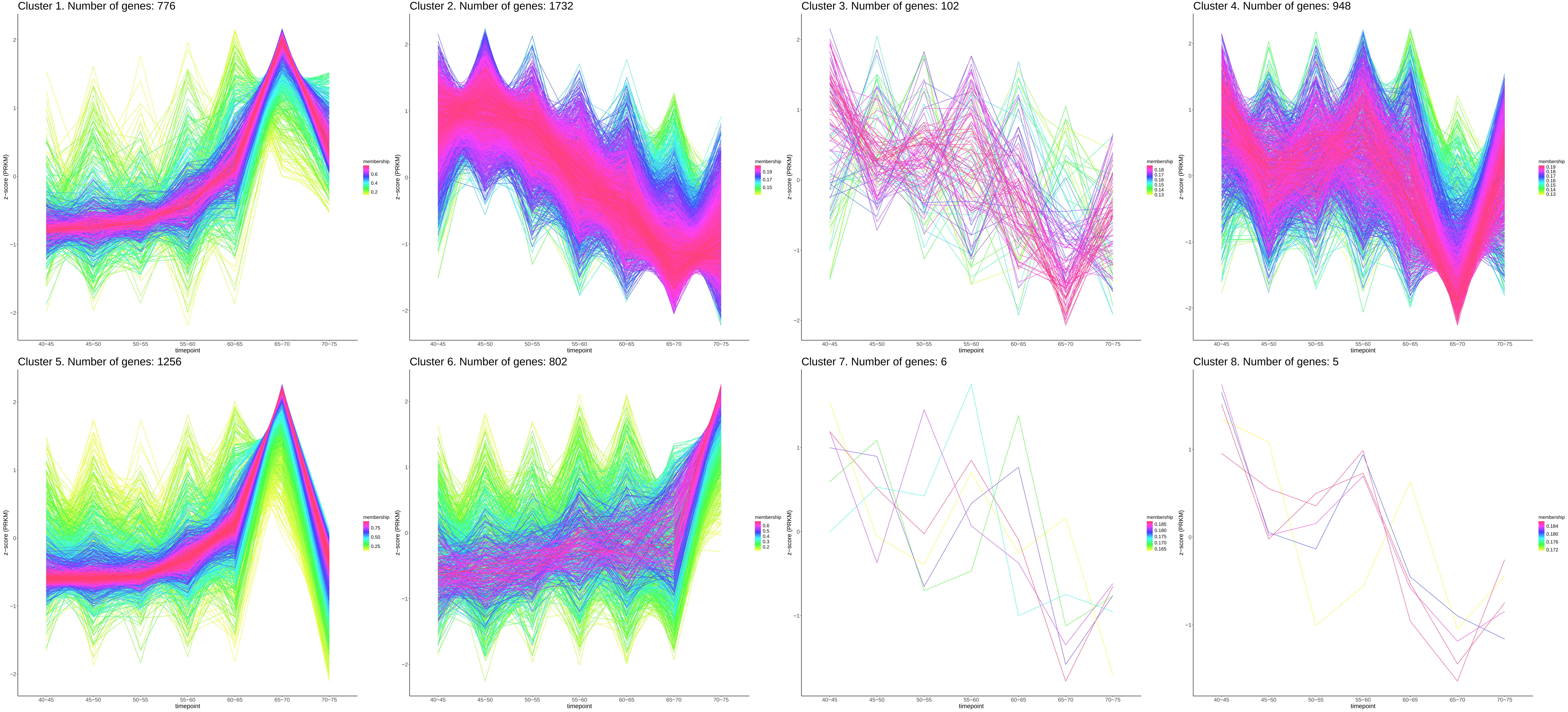

Lymphoid time clusters

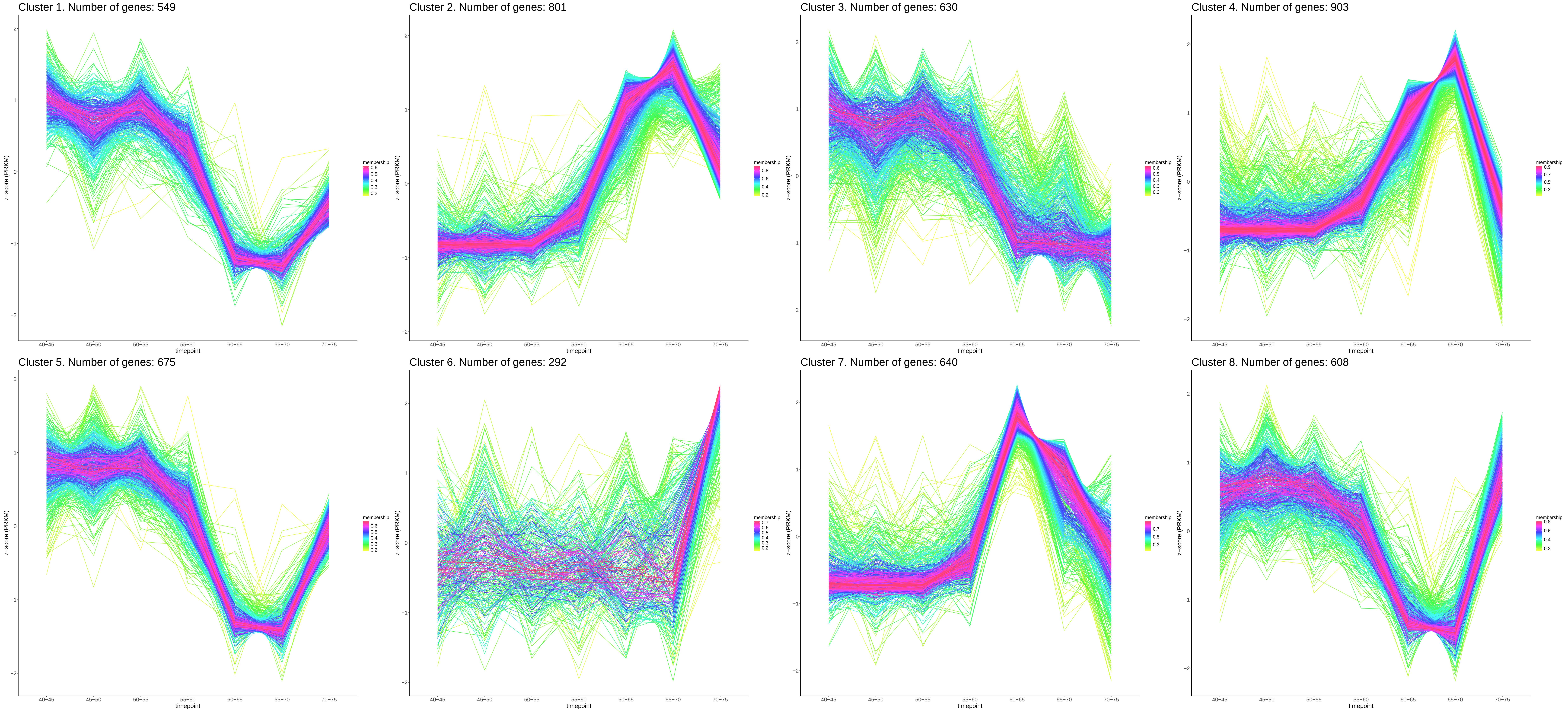

Mesothelial time clusters

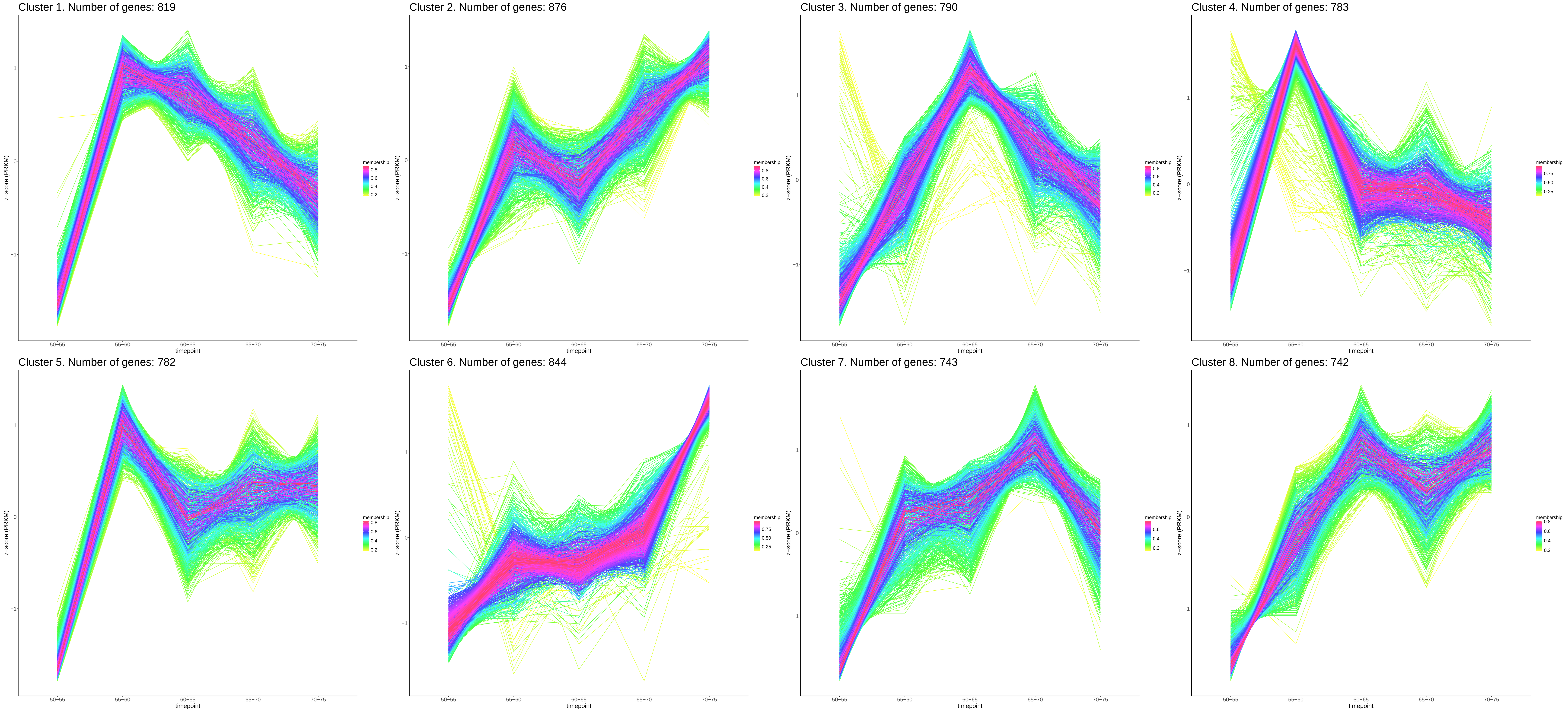

Myeloid time clusters

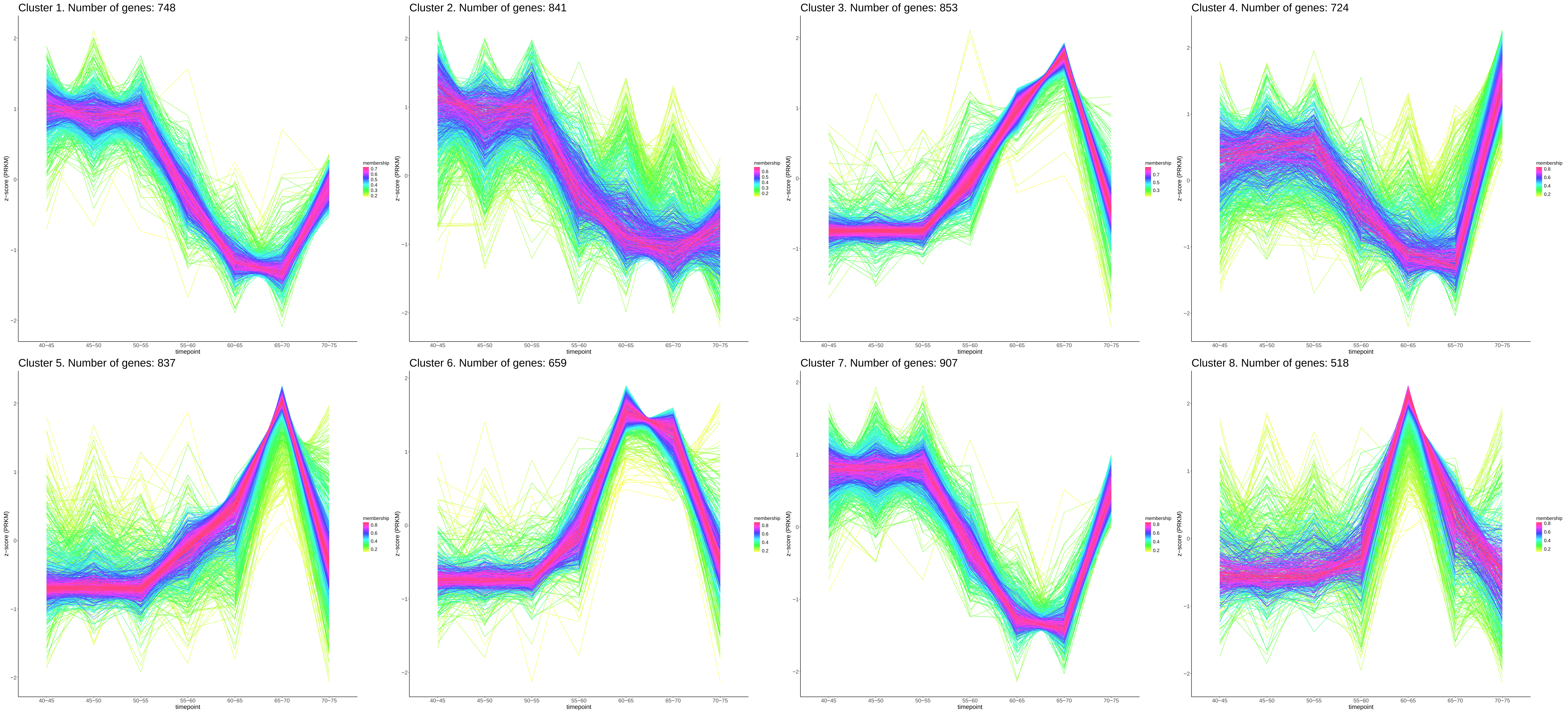

Neuronal time clusters

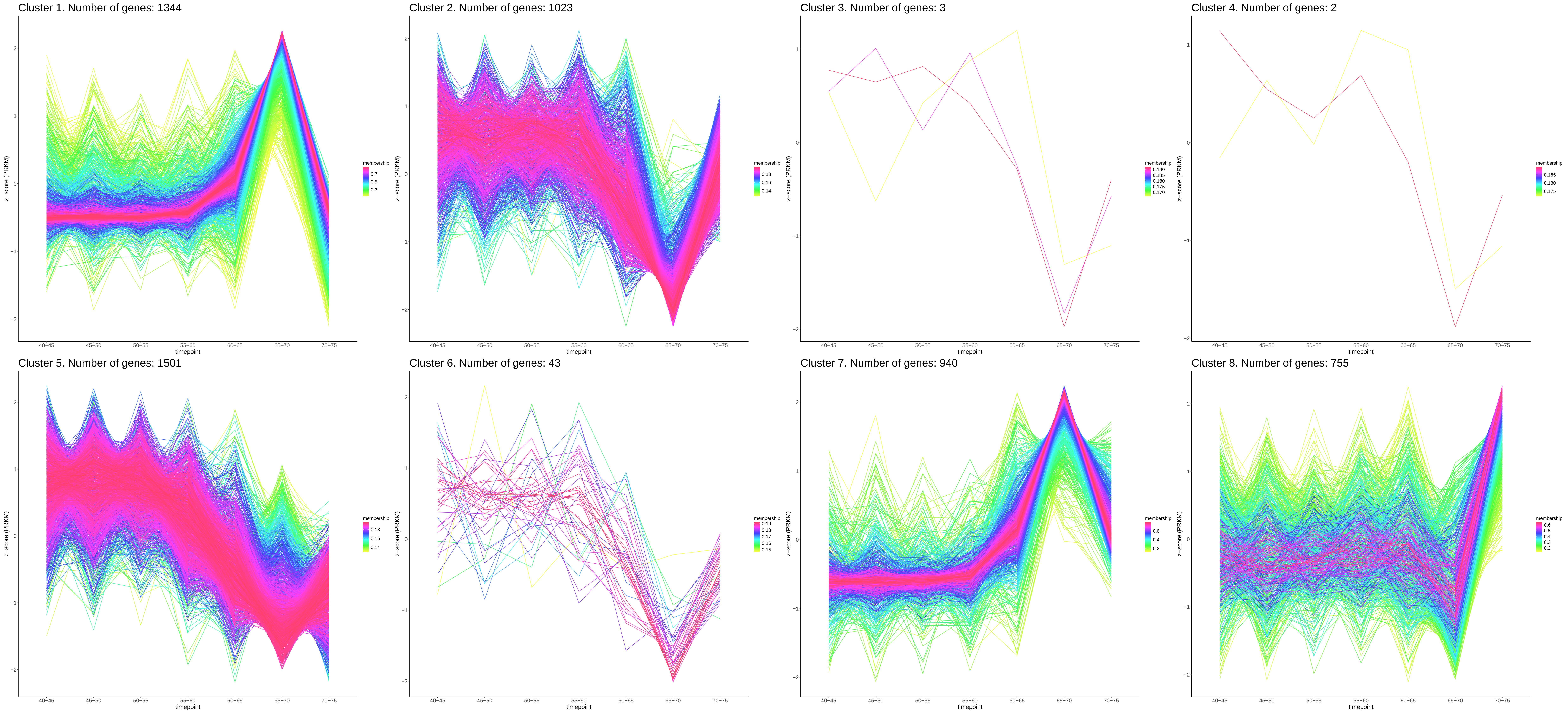

Pericytes time clusters

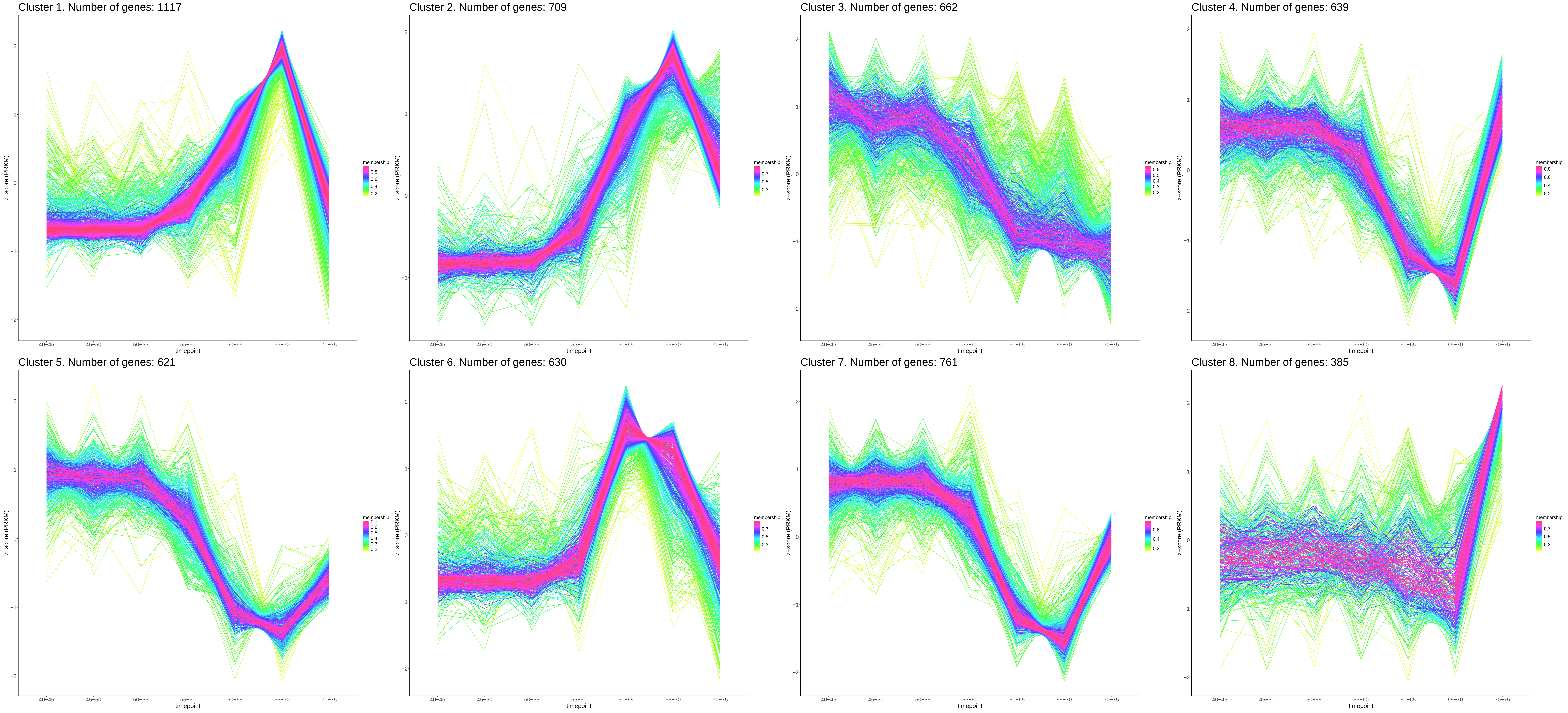

Smooth_muscle_cells time clusters

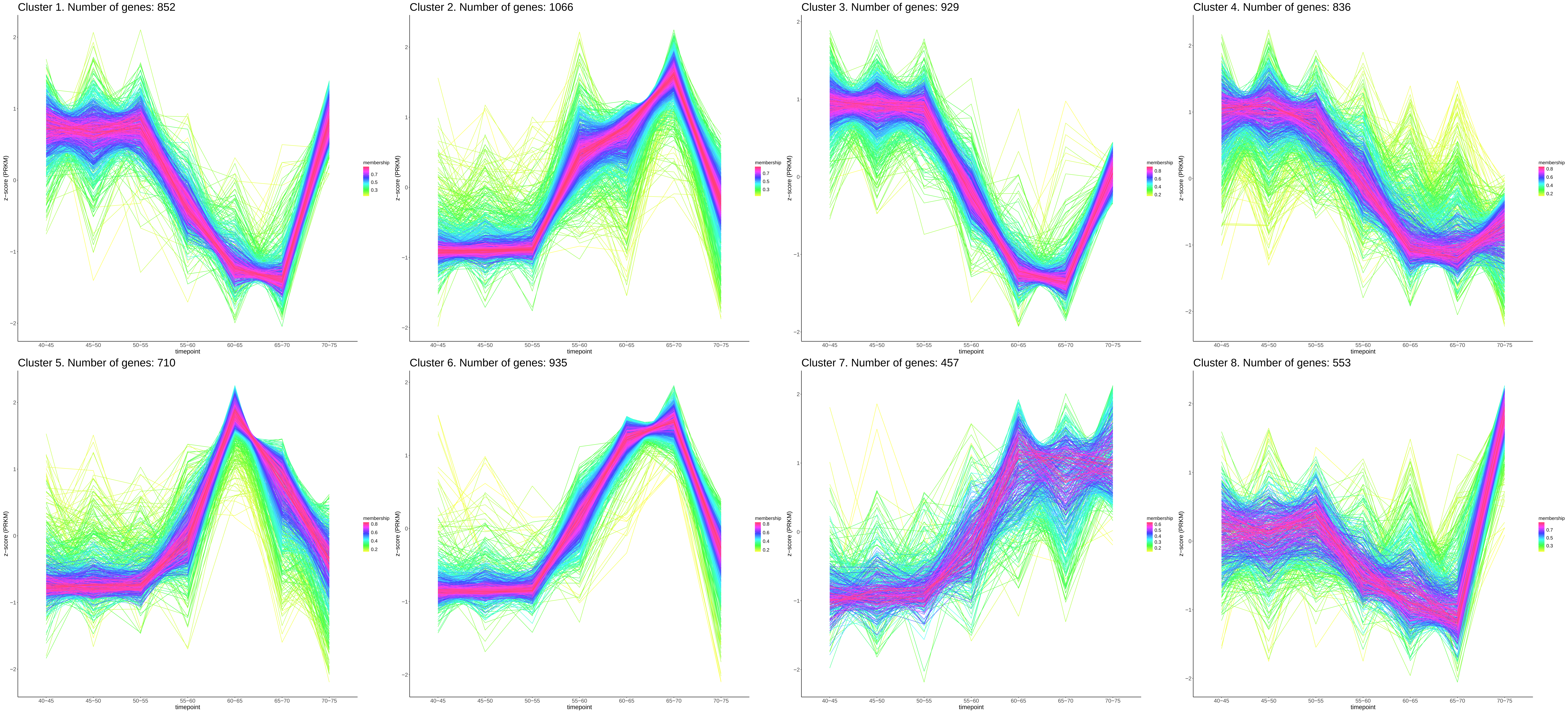

Ventricular_Cardiomyocyte time clusters

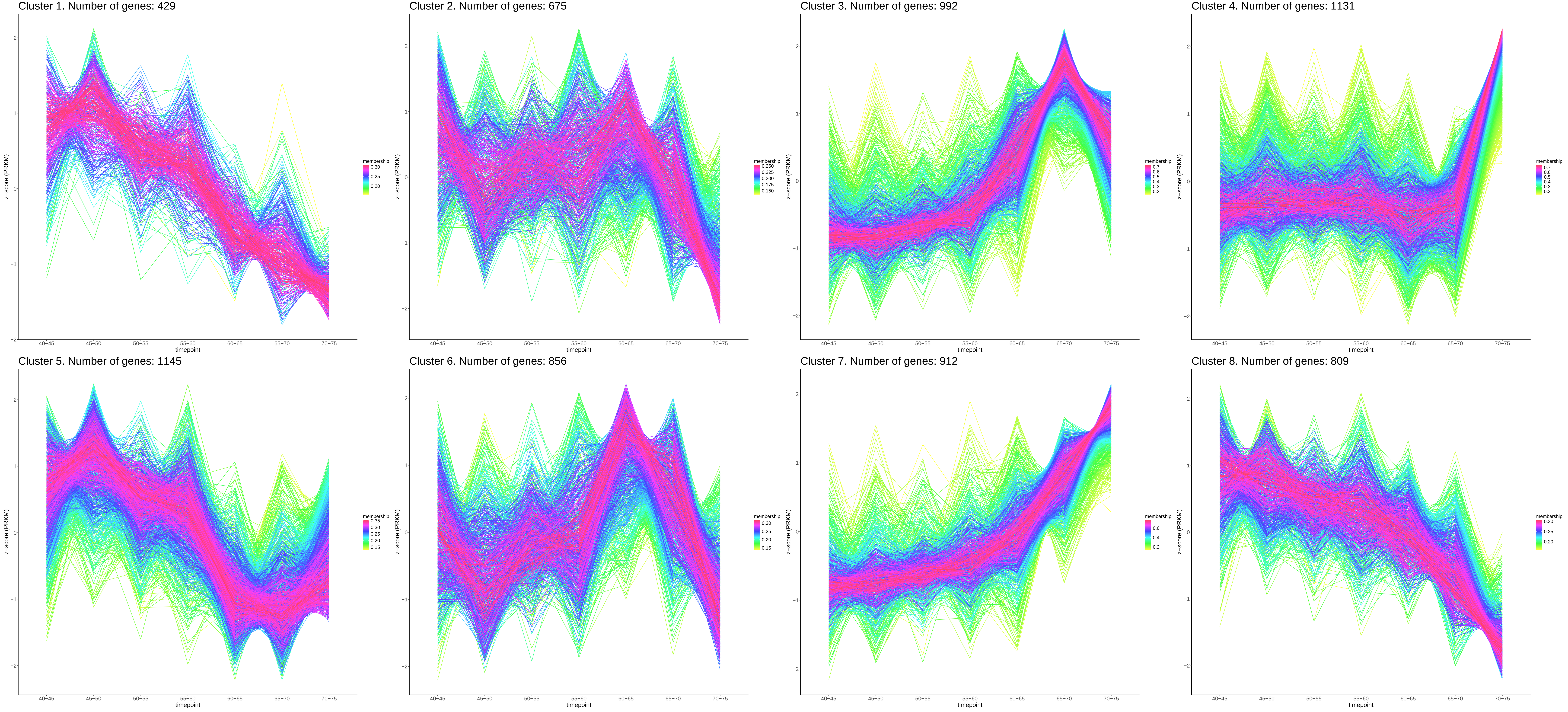

Supplement: lqae180_Supplemental_Files [file lqae180_supplemental_files.zip › FigS2-HeartCellAtlas_TCseqPlots.pdf]
