# Supplementary material for: Mining single-cell data for cell type–disease associations
Source: NAR Genom Bioinform. 2024 Dec 18;6(4):lqae180. doi: 10.1093/nargab/lqae180 (PMC11655289; doi:10.1093/nargab/lqae180)

H−Ast_Ependymal time clusters

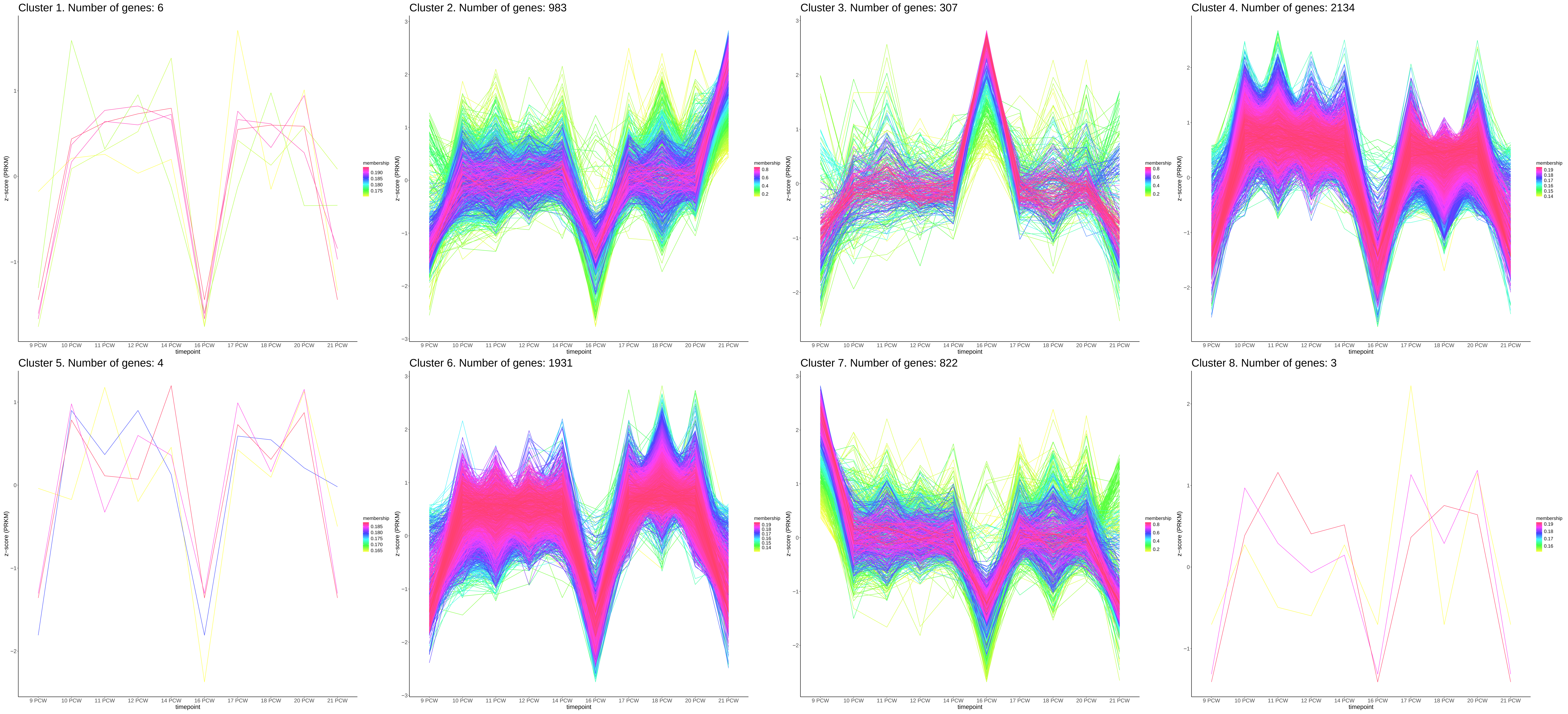

H−BG time clusters

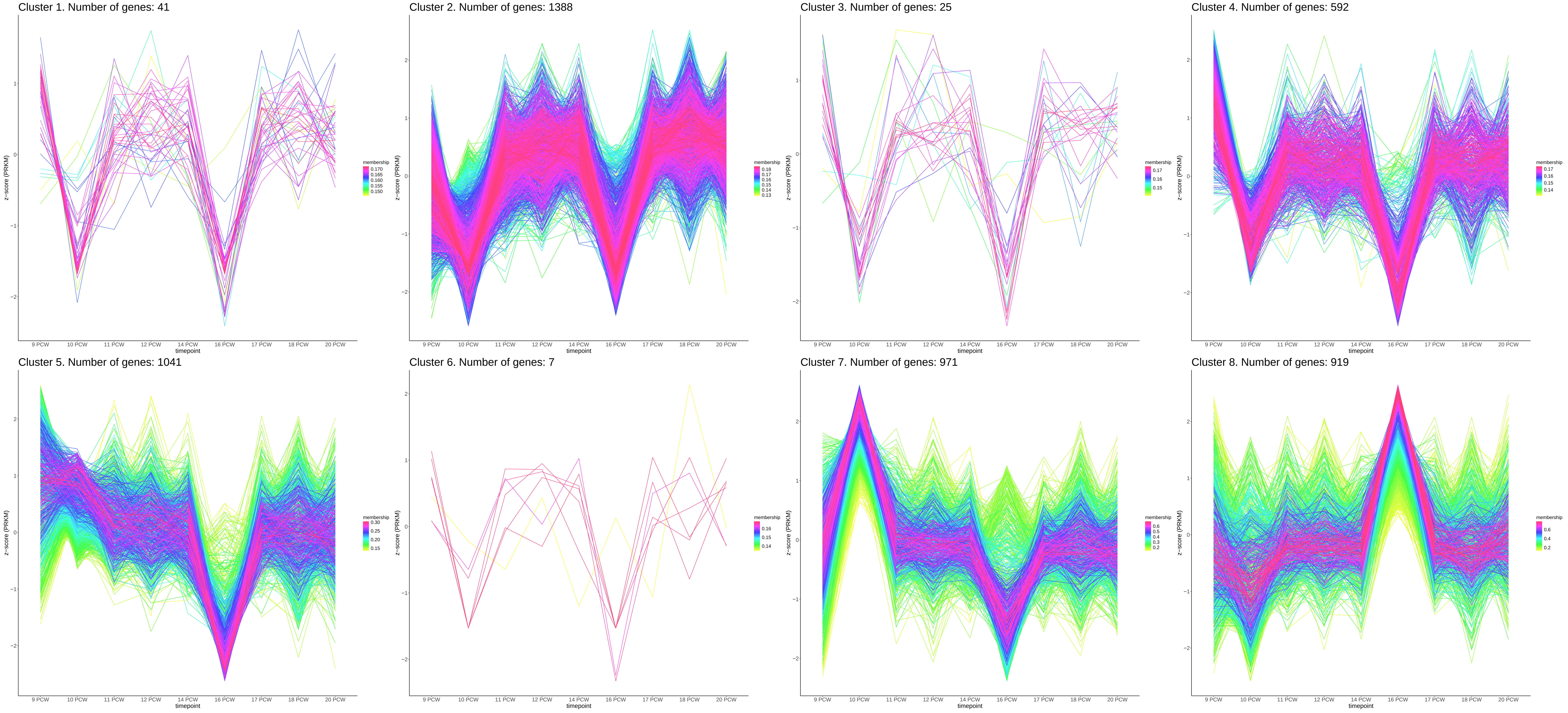

H−BS_Choroid_Ependymal time clusters

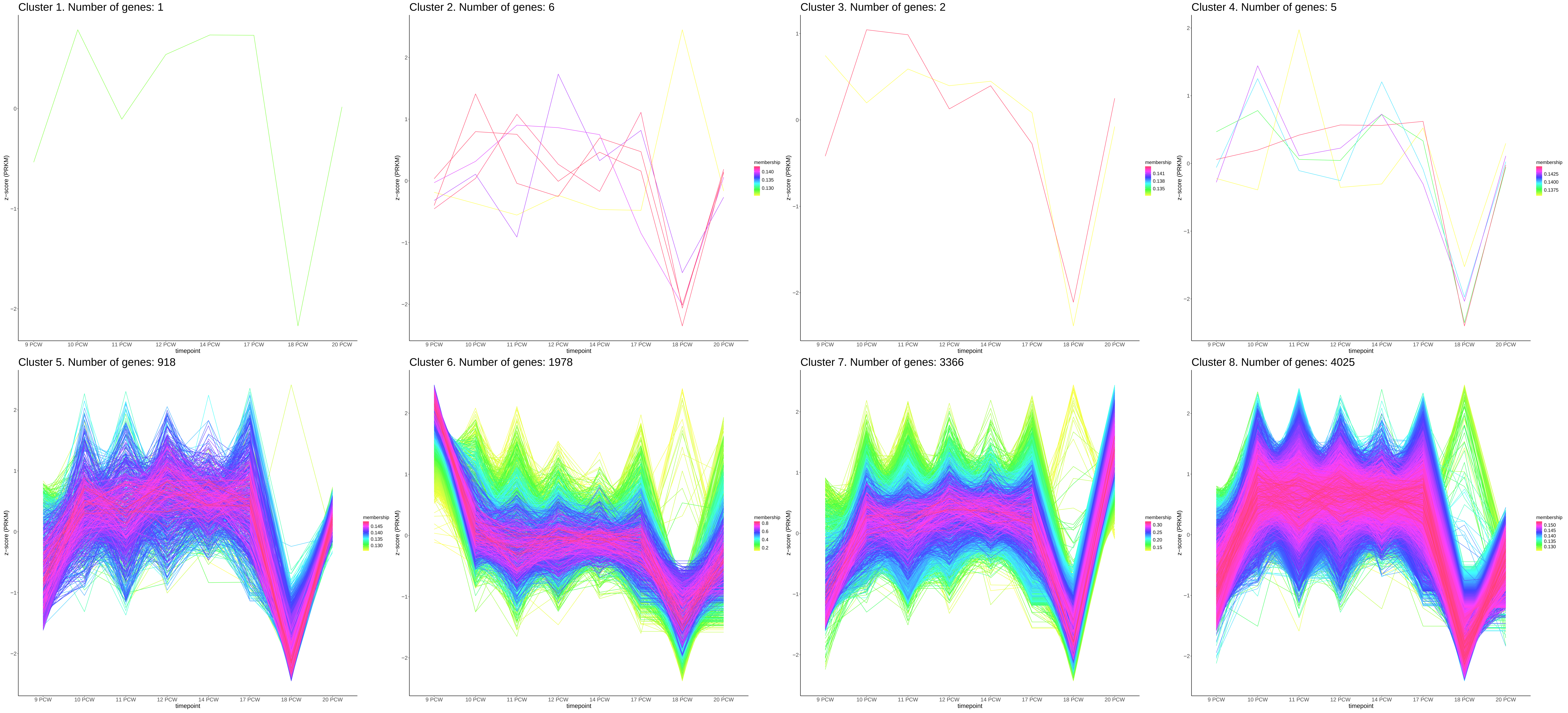

H−eCN_UBC time clusters

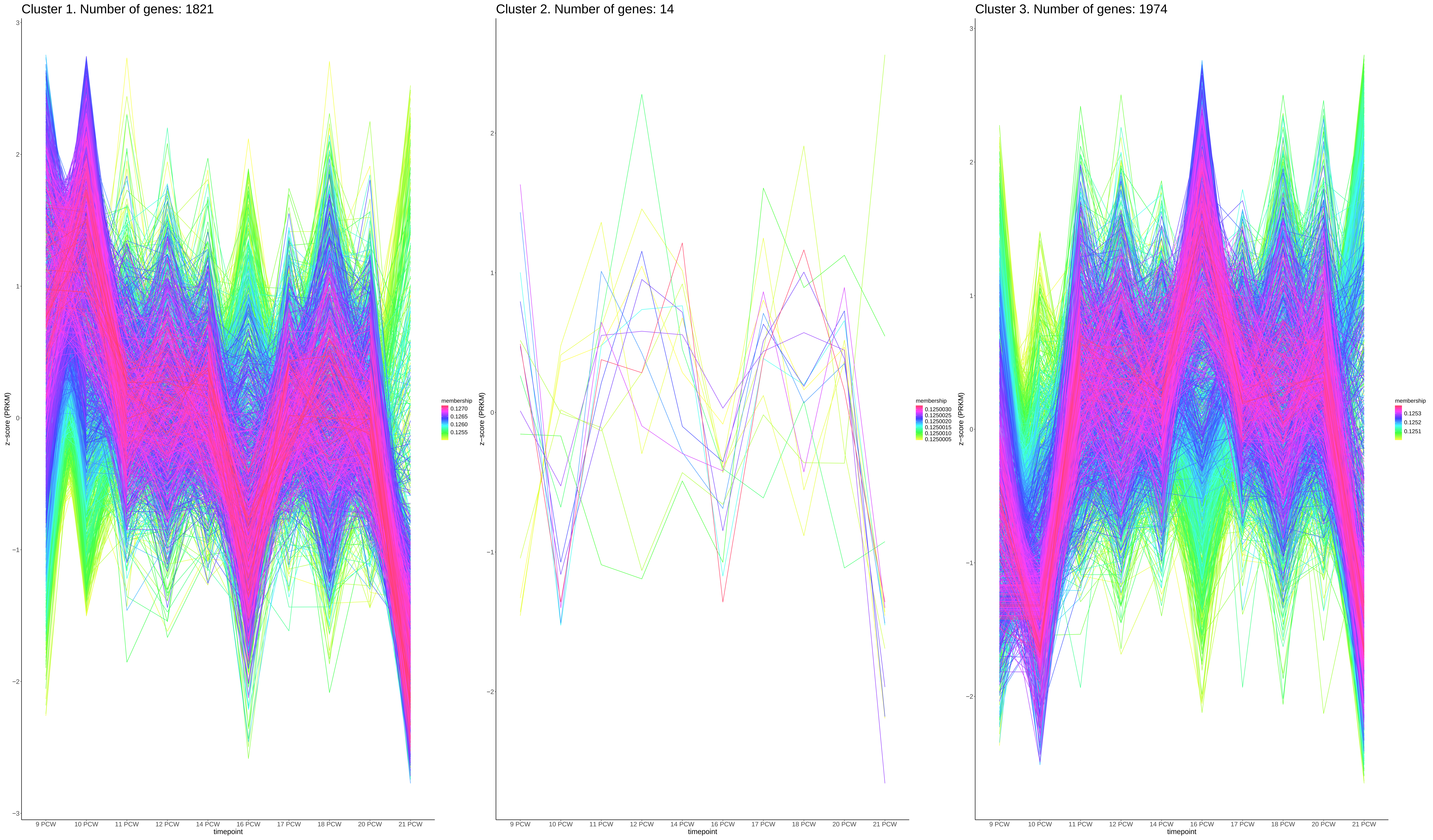

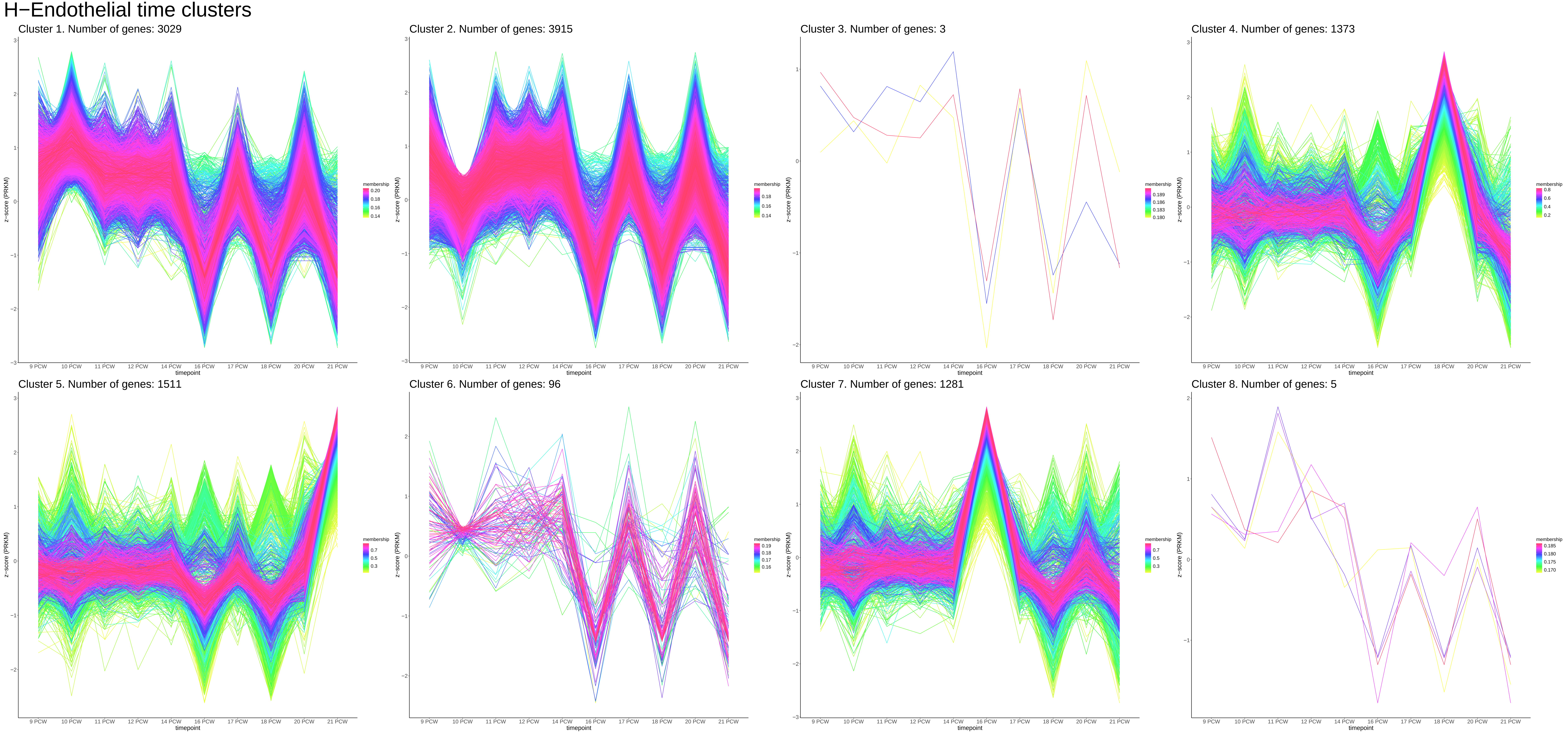
H−Endothelial time clusters
Cluster 1. Number of genes: 3029
Cluster 2. Number of genes: 3915
Cluster 3. Number of genes: 3
Cluster 4. Number of genes: 1373
Cluster 5. Number of genes: 1511
Cluster 6. Number of genes: 96
Cluster 7. Number of genes: 1281
Cluster 8. Number of genes: 5
z−score (PRKM)
timepoint
9 PCW
10 PCW
11 PCW
12 PCW
14 PCW
16 PCW
17 PCW
18 PCW
20 PCW
21 PCW
membership
0.20
0.18
0.16
0.14
0.189
0.186
0.183
0.180
0.8
0.6
0.4
0.2
0.7
0.5
0.3
0.19
0.17
0.185
0.175
0.170

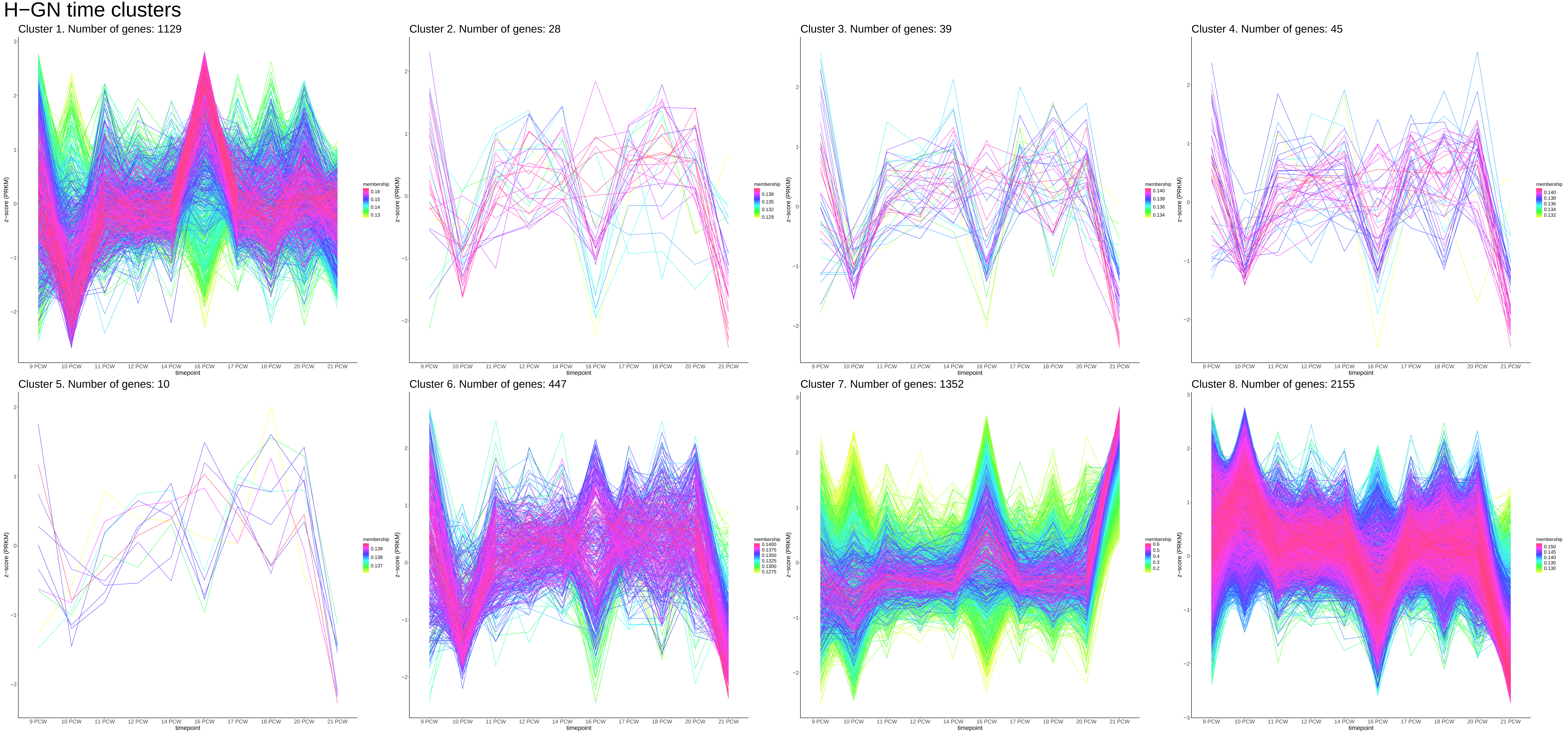
H−GN time clusters
Cluster 1. Number of genes: 1129
Cluster 2. Number of genes: 28
Cluster 3. Number of genes: 39
Cluster 4. Number of genes: 45
Cluster 5. Number of genes: 10
Cluster 6. Number of genes: 447
Cluster 7. Number of genes: 1352
Cluster 8. Number of genes: 2155
z−score (PRKM)
timepoint
9 PCW
10 PCW
11 PCW
12 PCW
14 PCW
16 PCW
17 PCW
18 PCW
20 PCW
21 PCW
membership

H−iCN time clusters

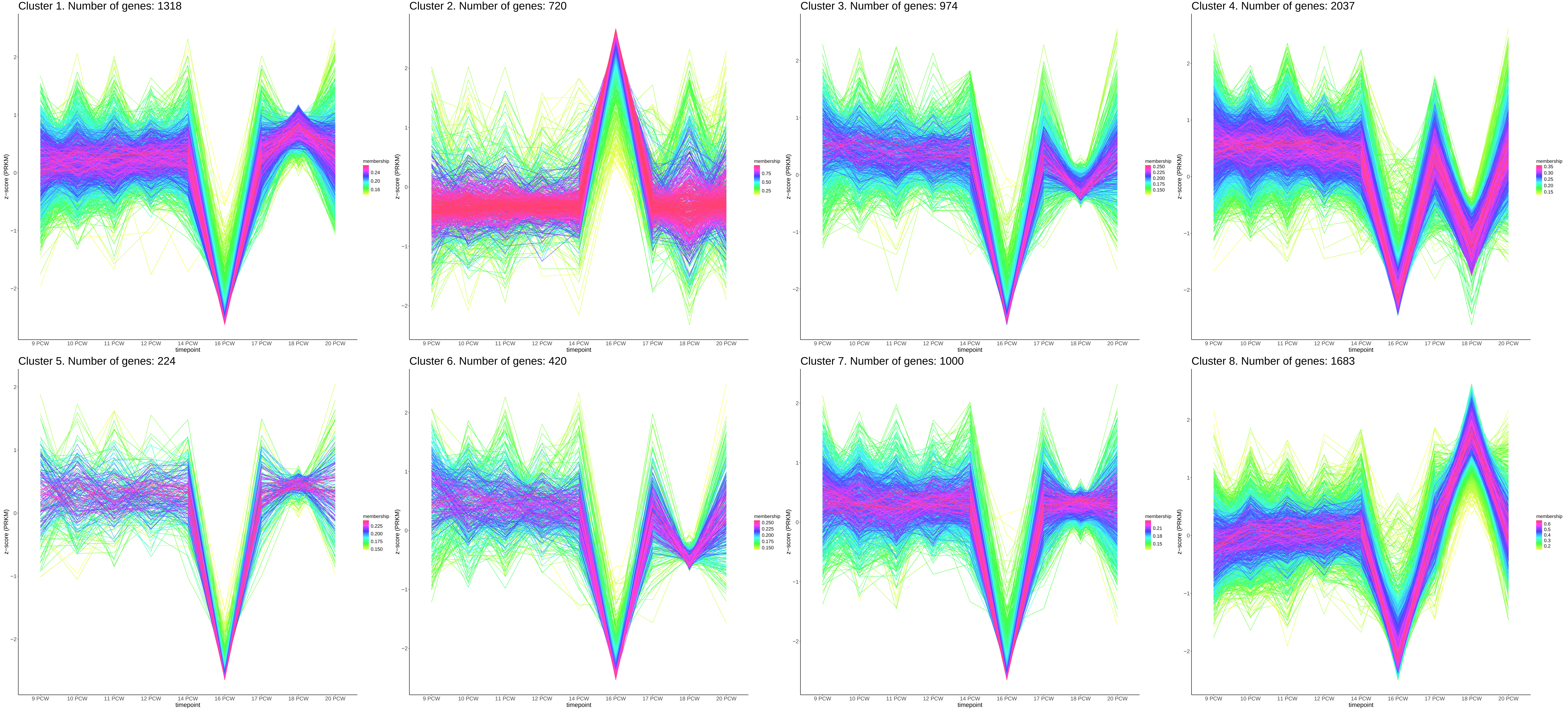

H−PC time clusters

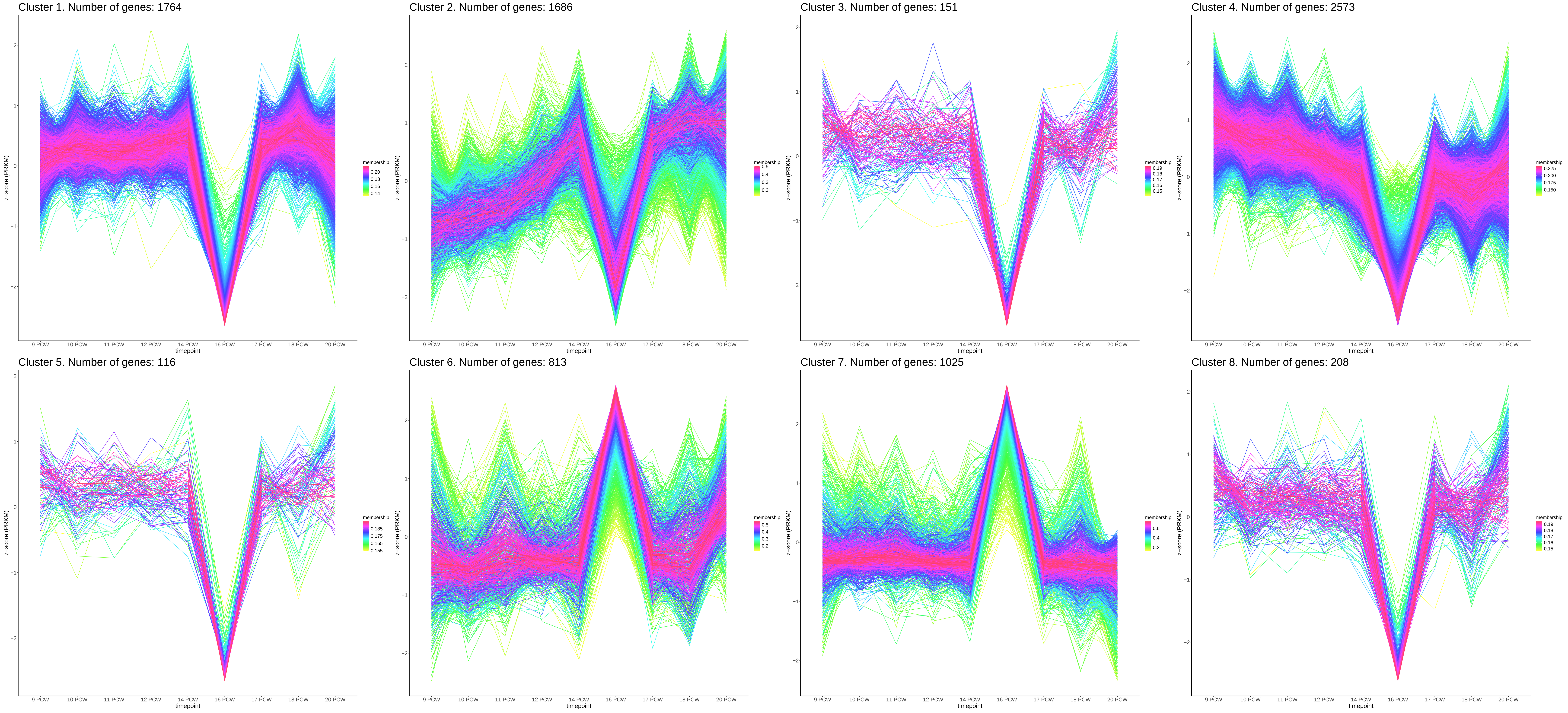

Supplement: lqae180_Supplemental_Files [file lqae180_supplemental_files.zip › FigS3-FetalCerebellarAtlas_TCseqPlots.pdf]
