# Supplementary material for: Mining single-cell data for cell type–disease associations
Source: NAR Genom Bioinform. 2024 Dec 18;6(4):lqae180. doi: 10.1093/nargab/lqae180 (PMC11655289; doi:10.1093/nargab/lqae180)

Airway_Smooth_Muscle time clusters

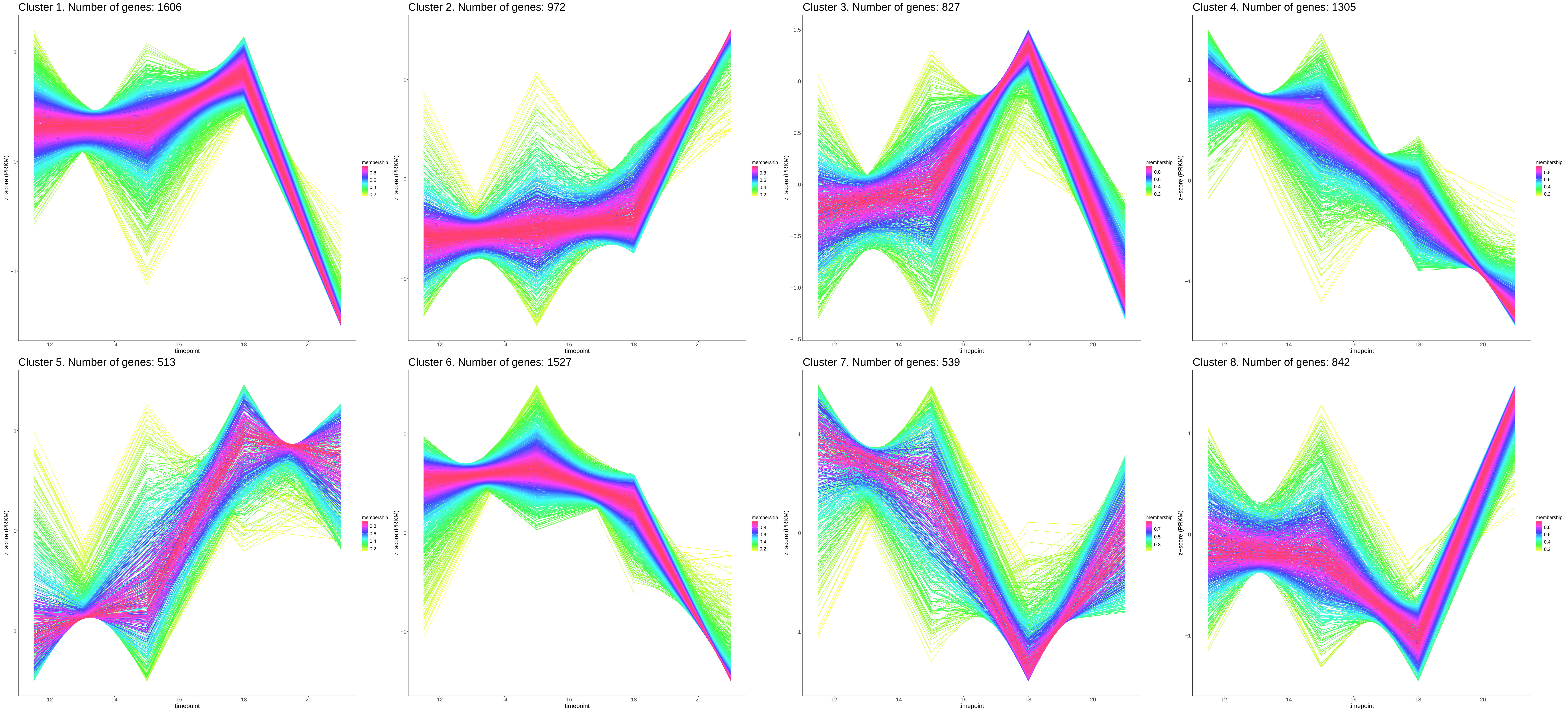

Basal_cell time clusters

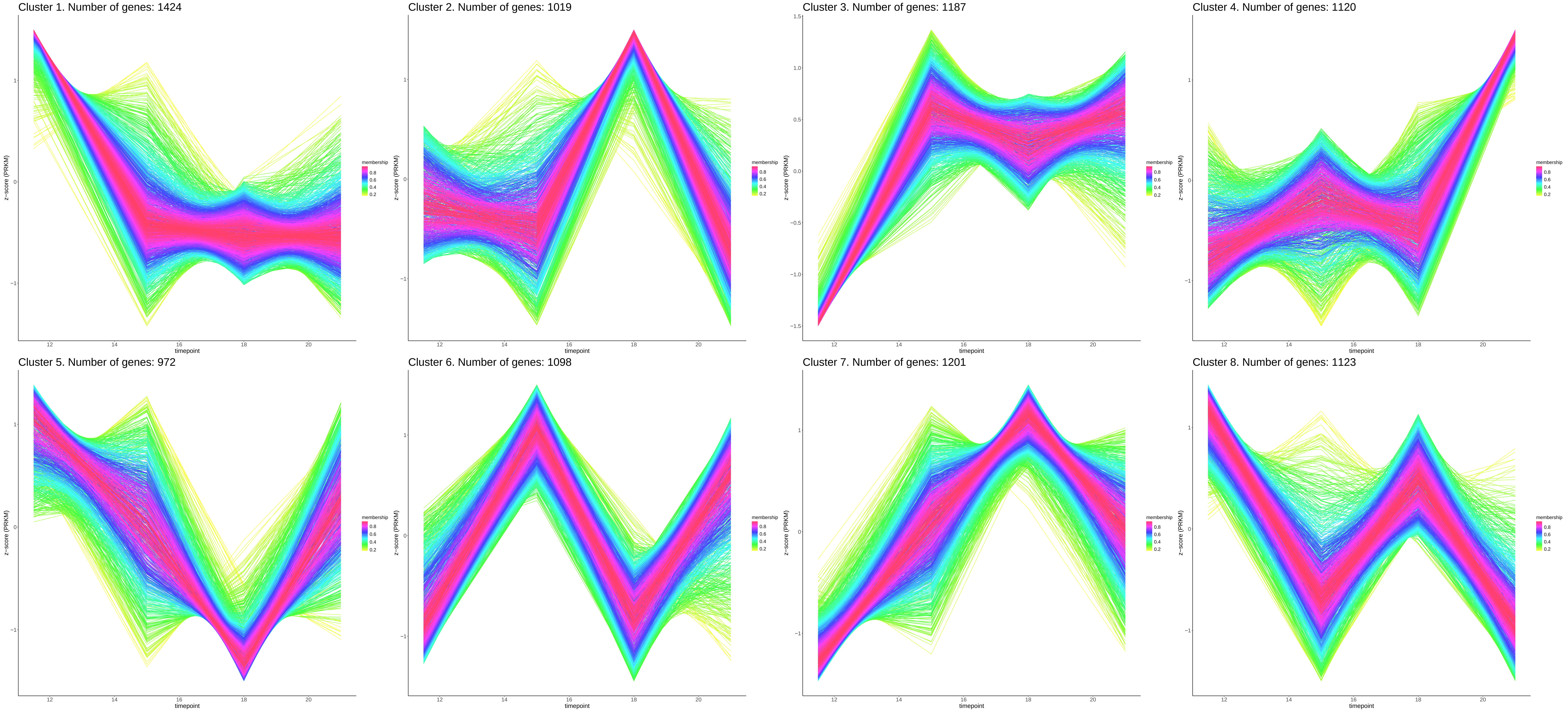

Bud_tip_adjacent time clusters

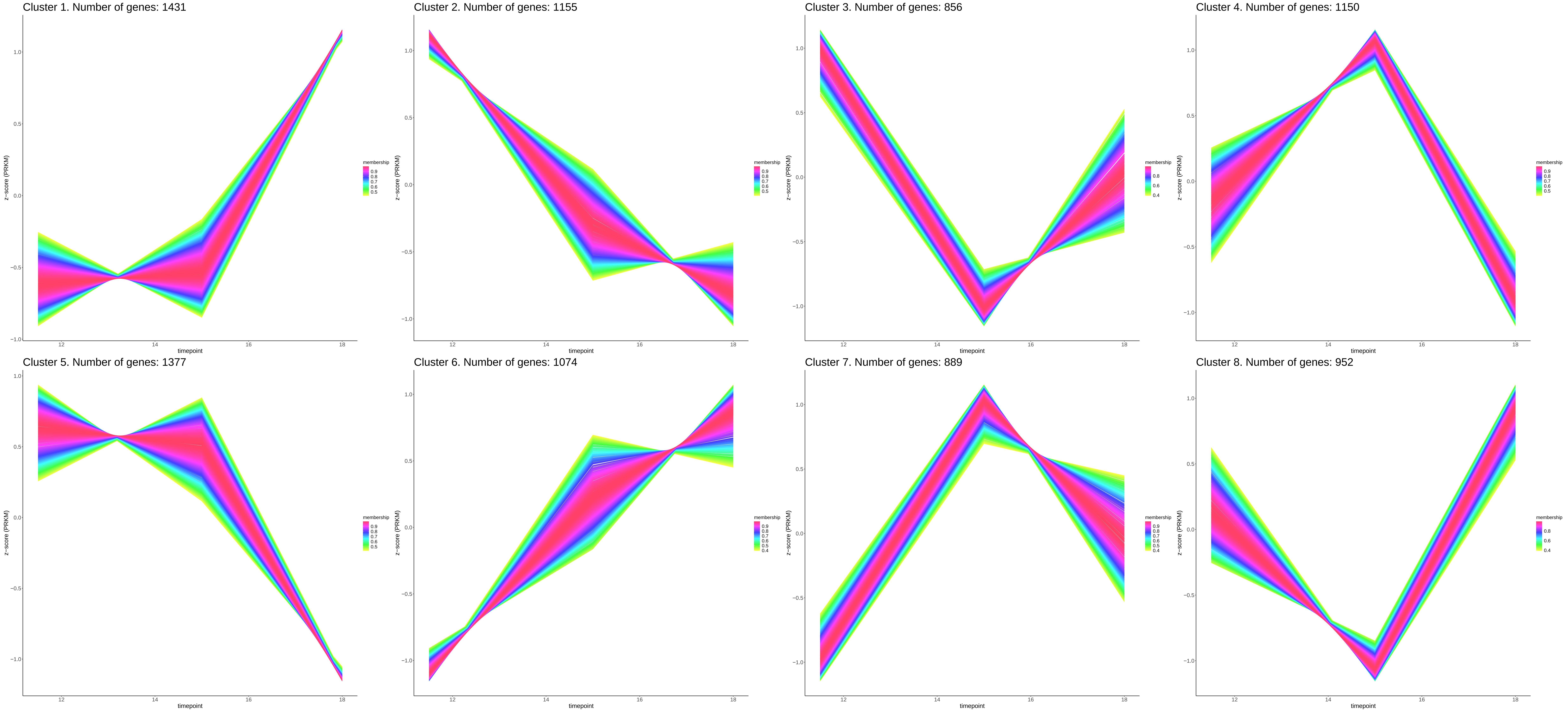

Bud_tip_progenitor time clusters

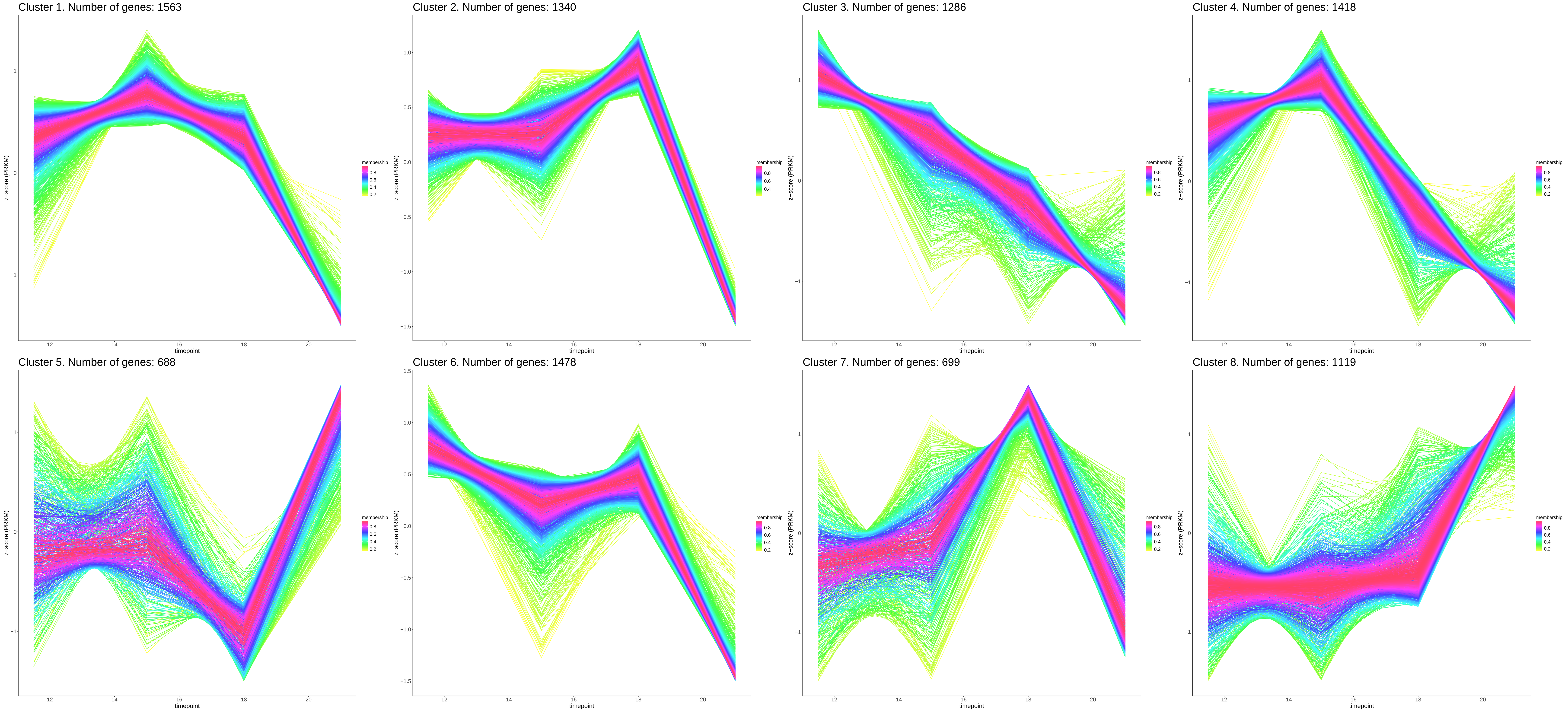

Cartilage time clusters

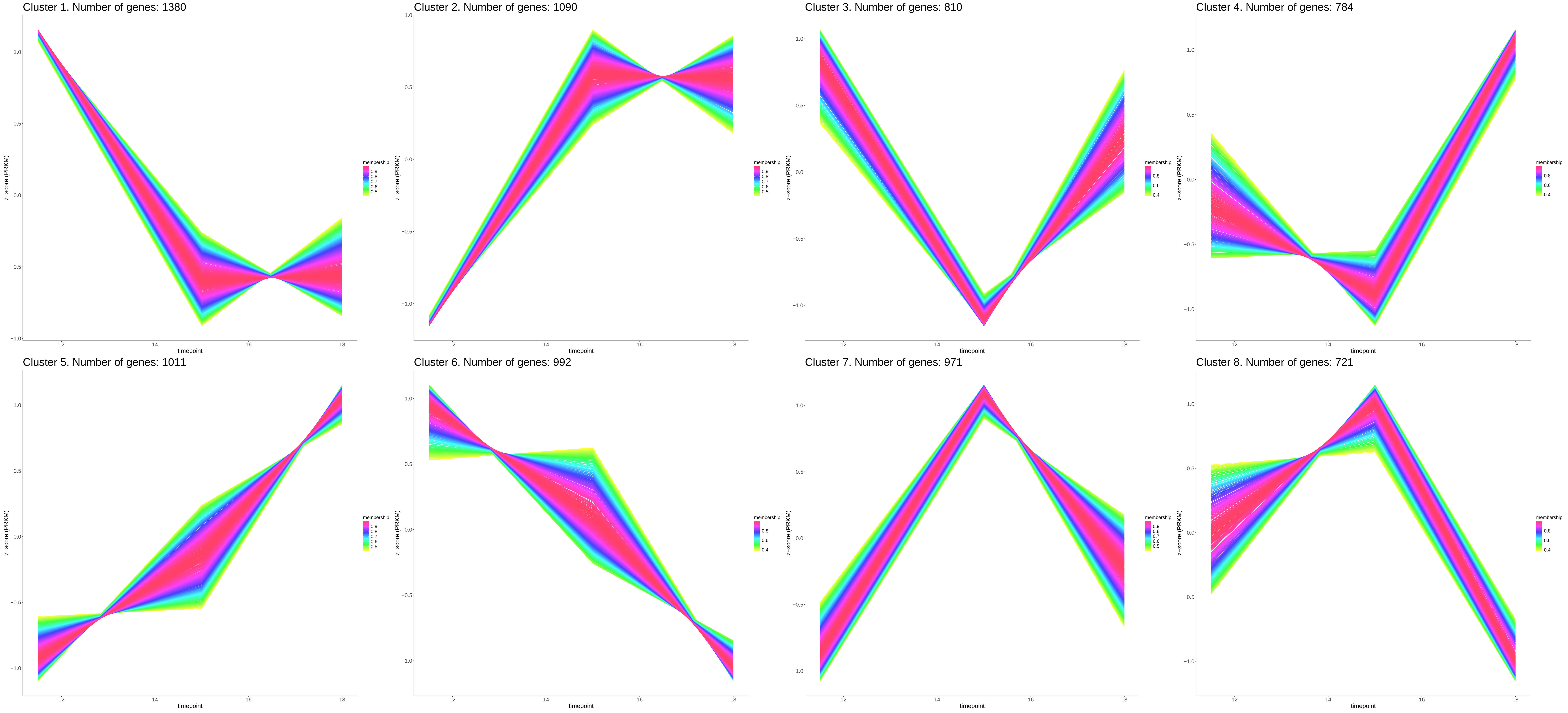

Club−like_secretory time clusters

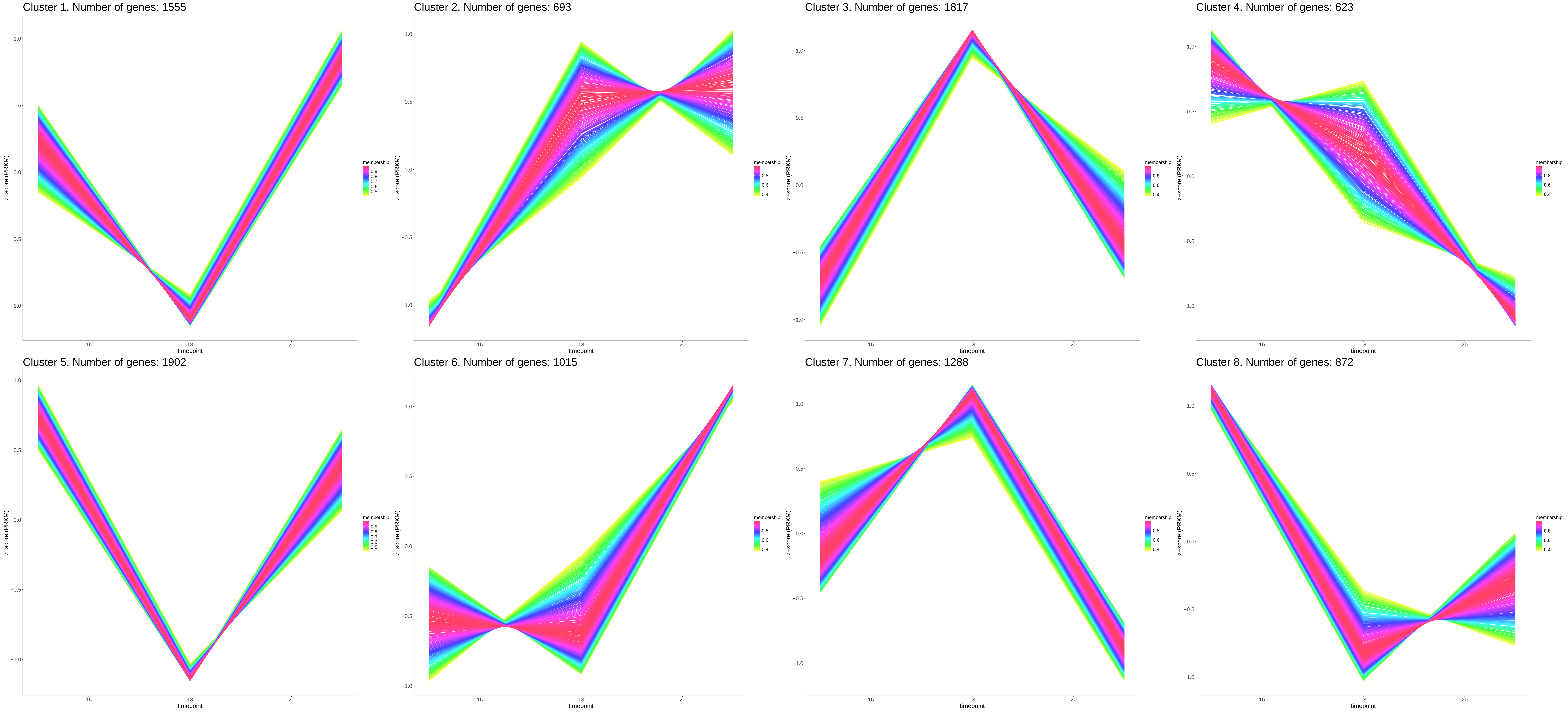

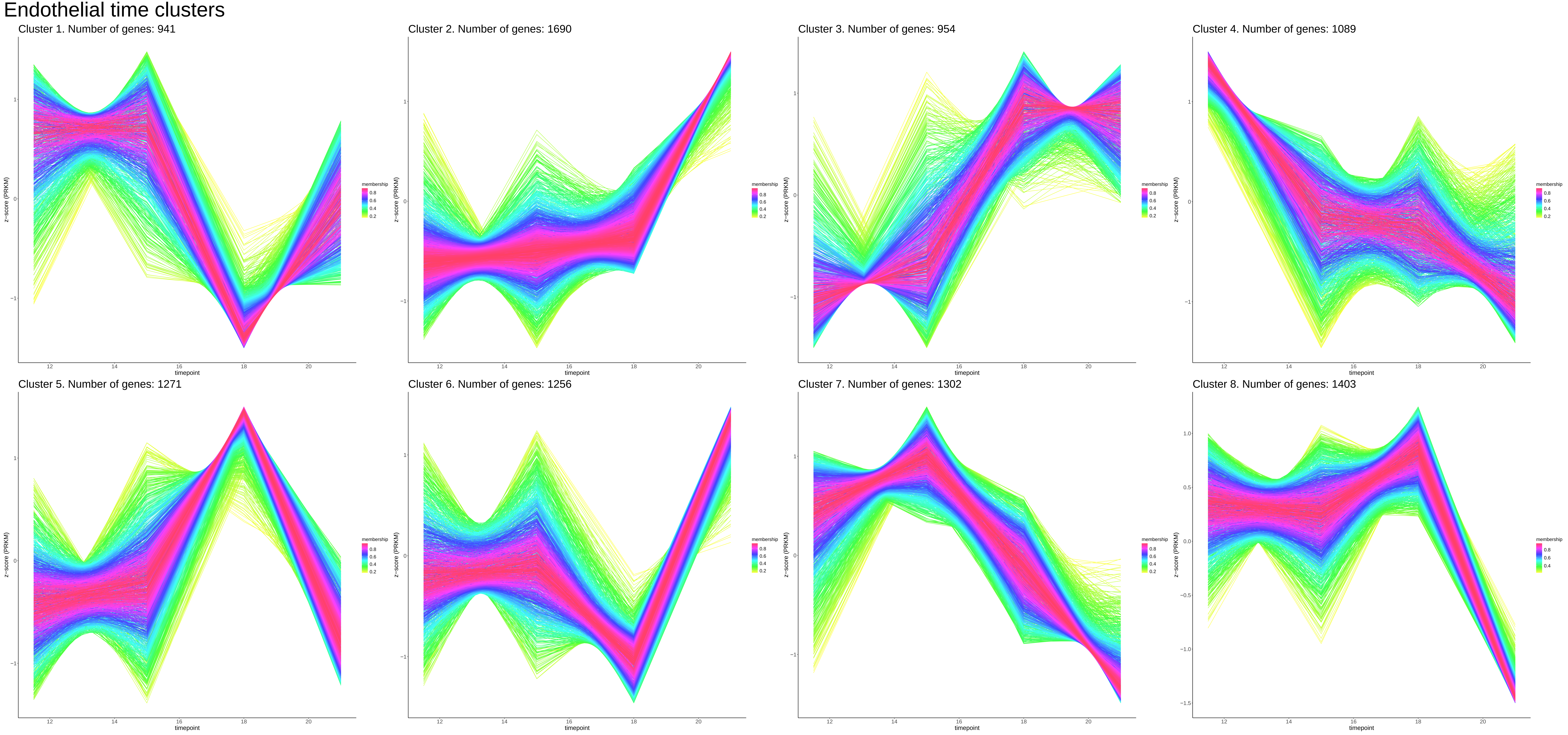
Endothelial time clusters
Cluster 1. Number of genes: 941
Cluster 2. Number of genes: 1690
Cluster 3. Number of genes: 954
Cluster 4. Number of genes: 1089
Cluster 5. Number of genes: 1271
Cluster 6. Number of genes: 1256
Cluster 7. Number of genes: 1302
Cluster 8. Number of genes: 1403
z−score (PRKM)
timepoint
membership

Epithelial time clusters

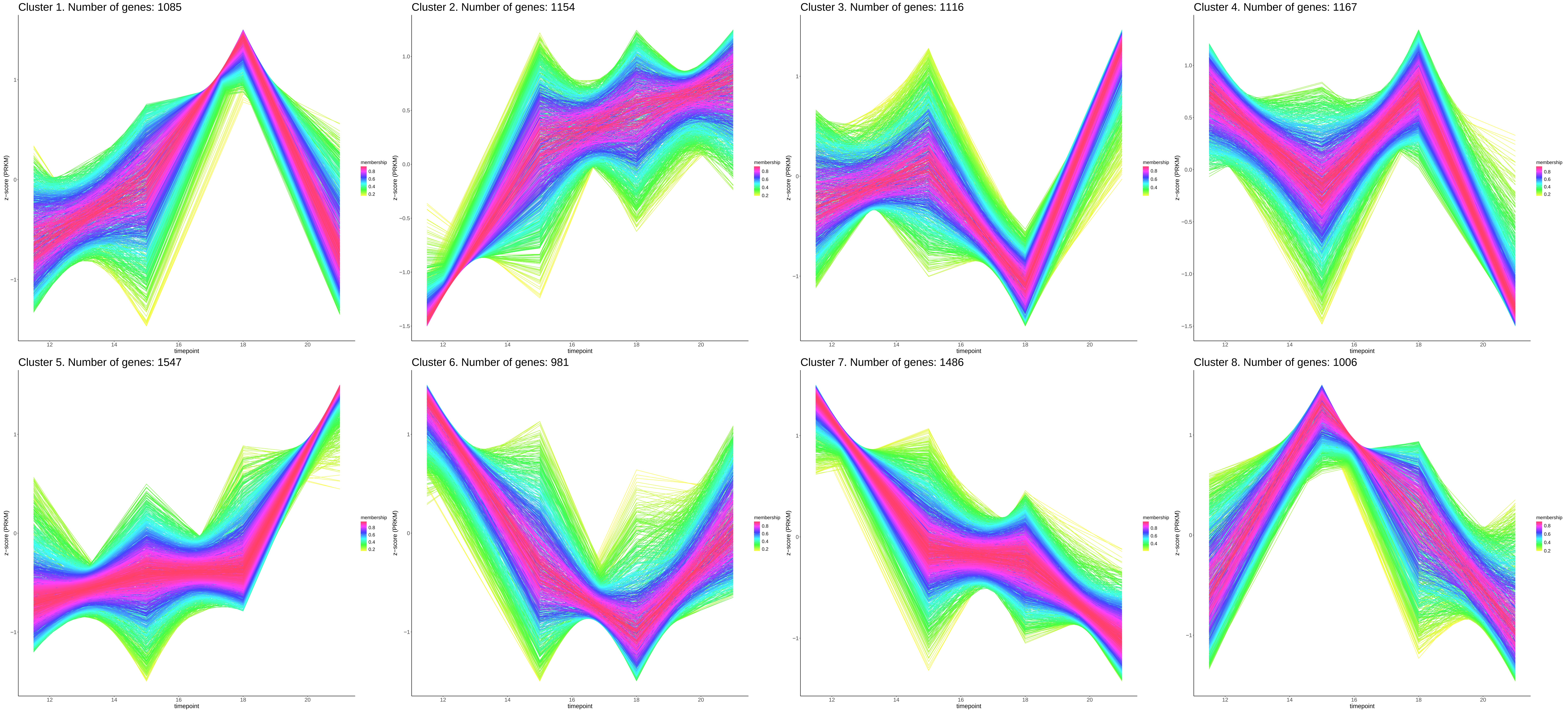

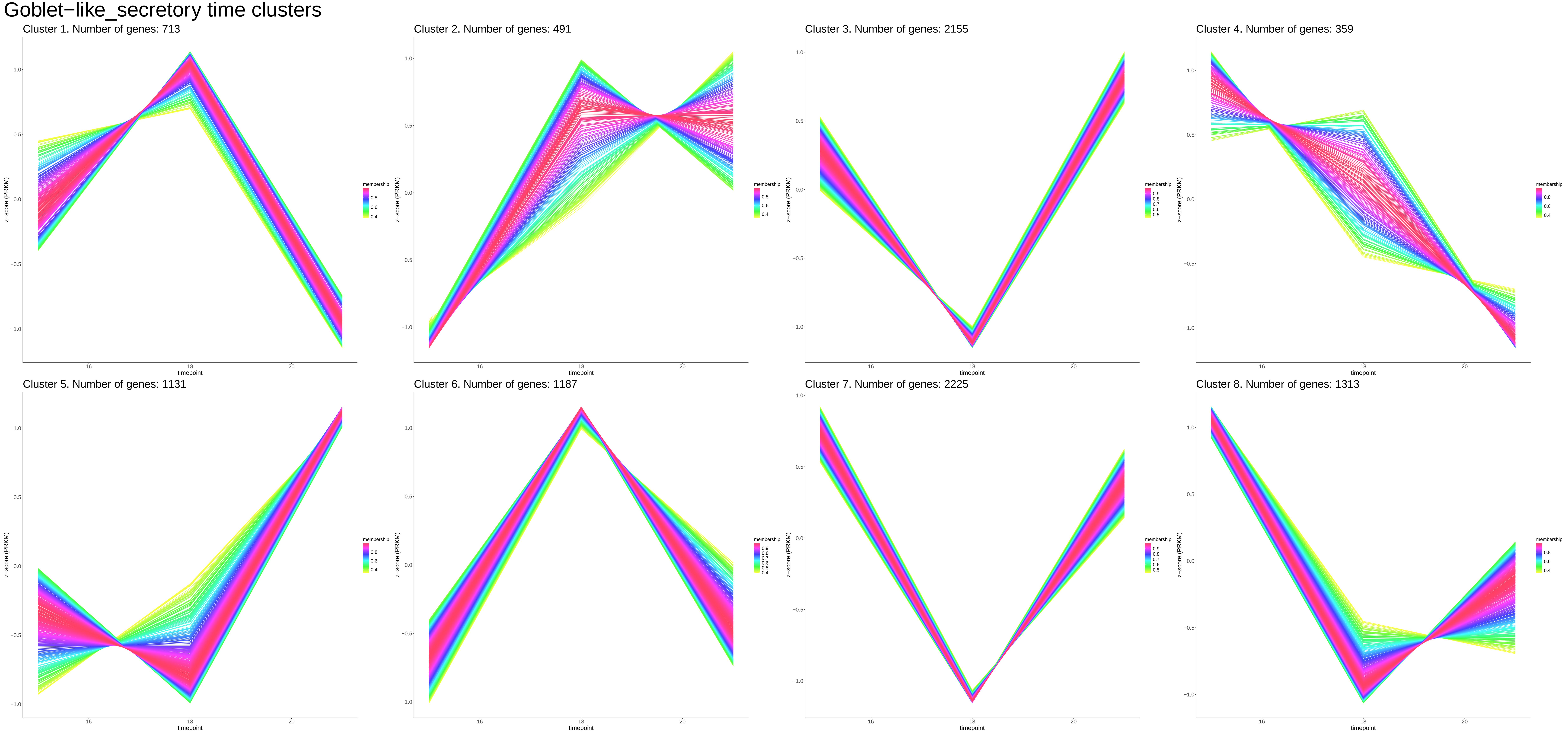
Goblet−like_secretory time clusters
Cluster 1. Number of genes: 713
Cluster 2. Number of genes: 491
Cluster 3. Number of genes: 2155
Cluster 4. Number of genes: 359
Cluster 5. Number of genes: 1131
Cluster 6. Number of genes: 1187
Cluster 7. Number of genes: 2225
Cluster 8. Number of genes: 1313
z−score (PRKM)
timepoint
16
18
20
membership

Hematopoietic,_B_Cells time clusters

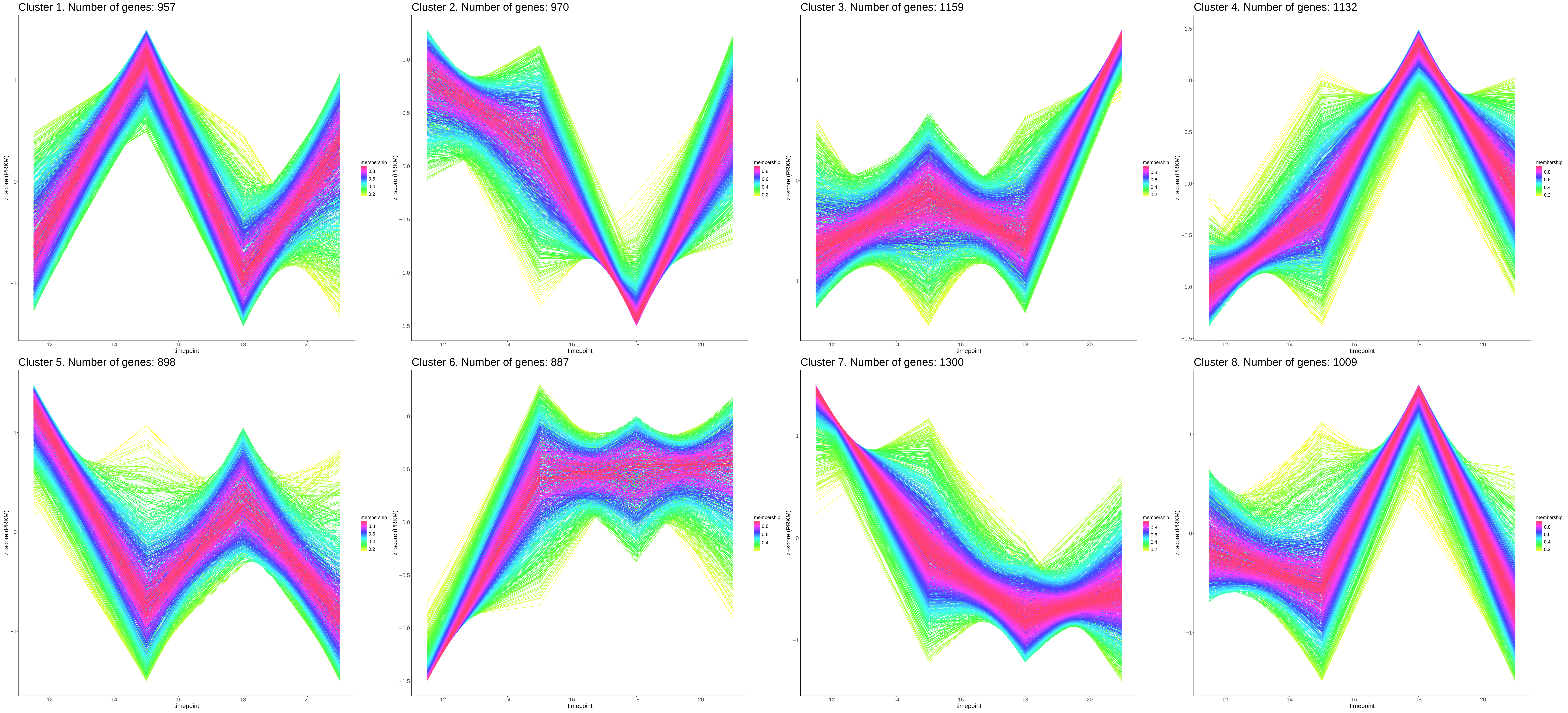

Hematopoietic,_Macrophage time clusters

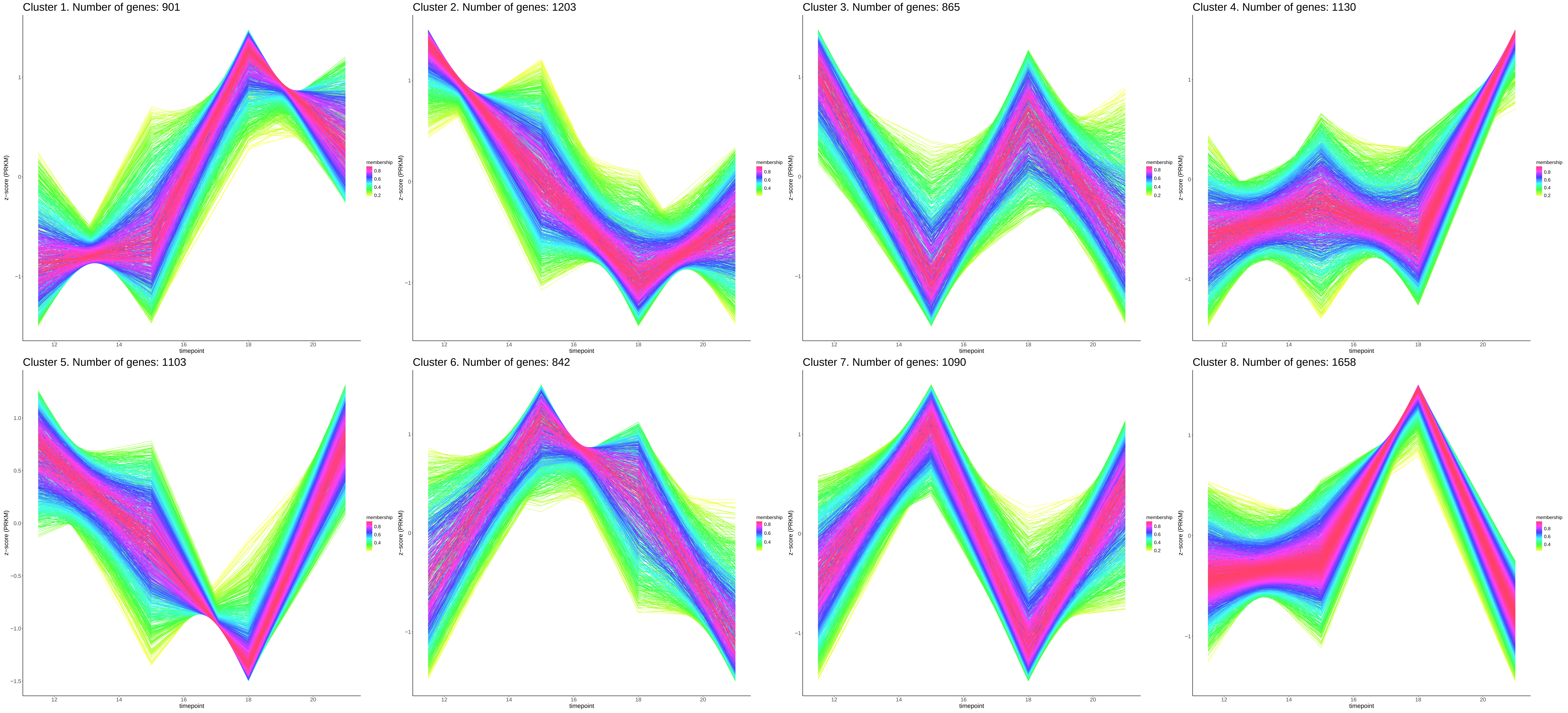

Hematopoietic,_Natural_Killer_Cell time clusters

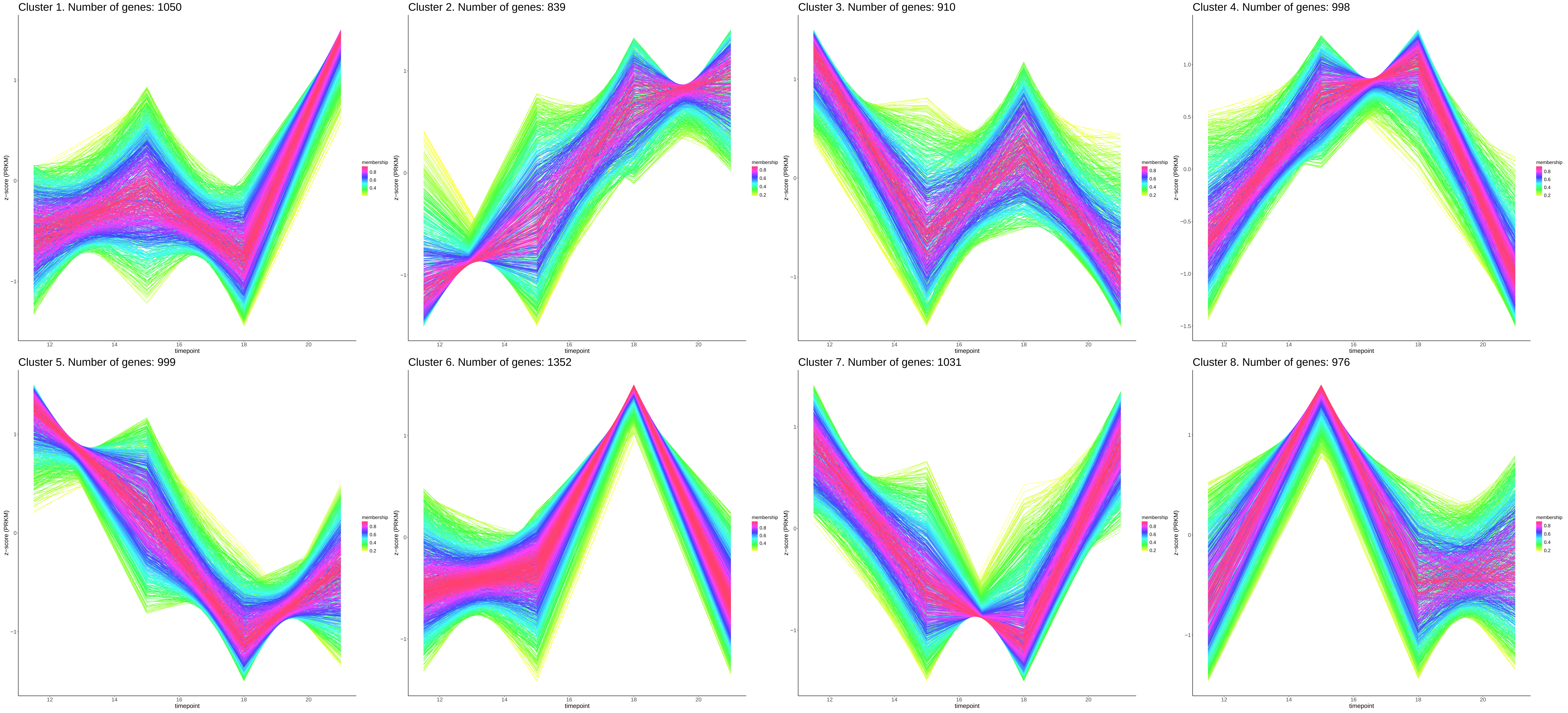

Hematopoietic,_T_Cells time clusters

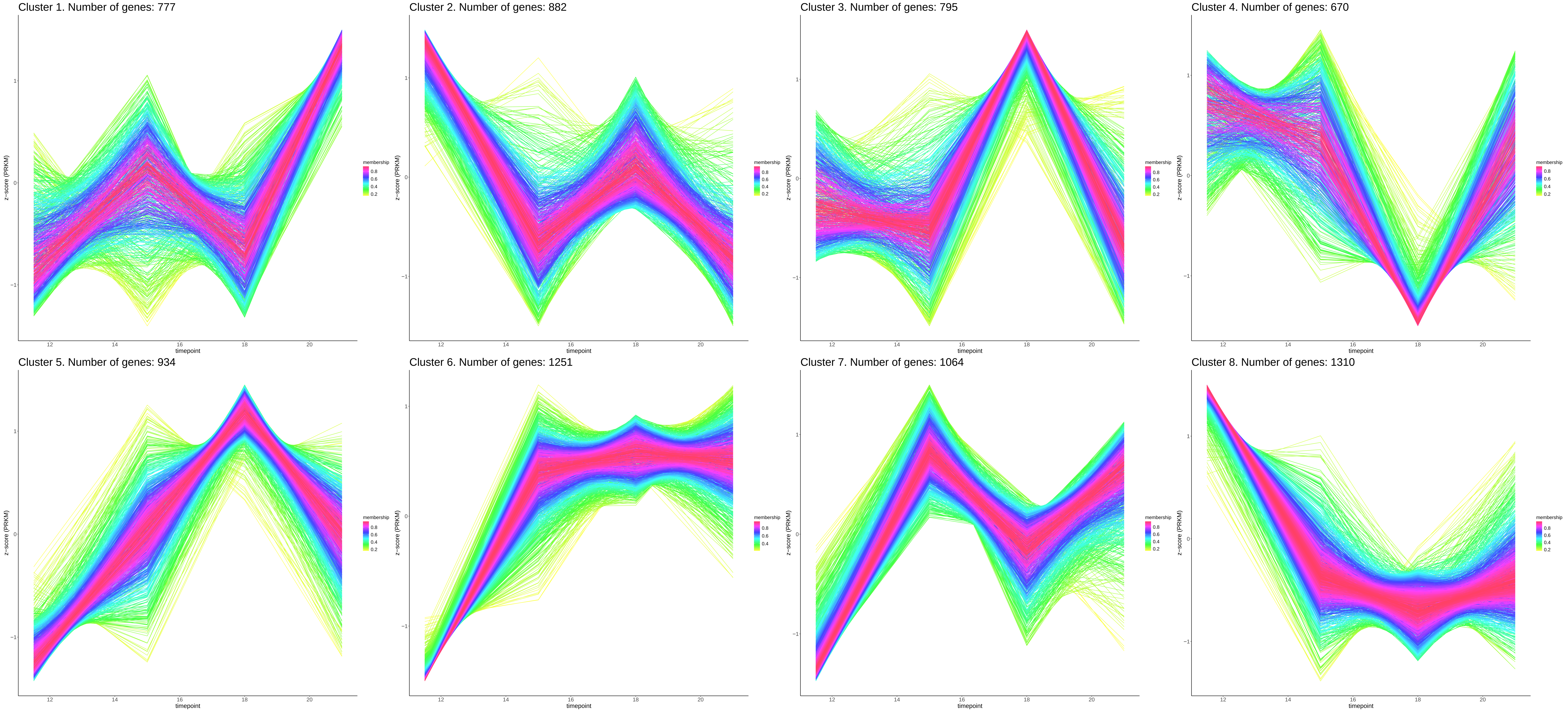

Immune time clusters

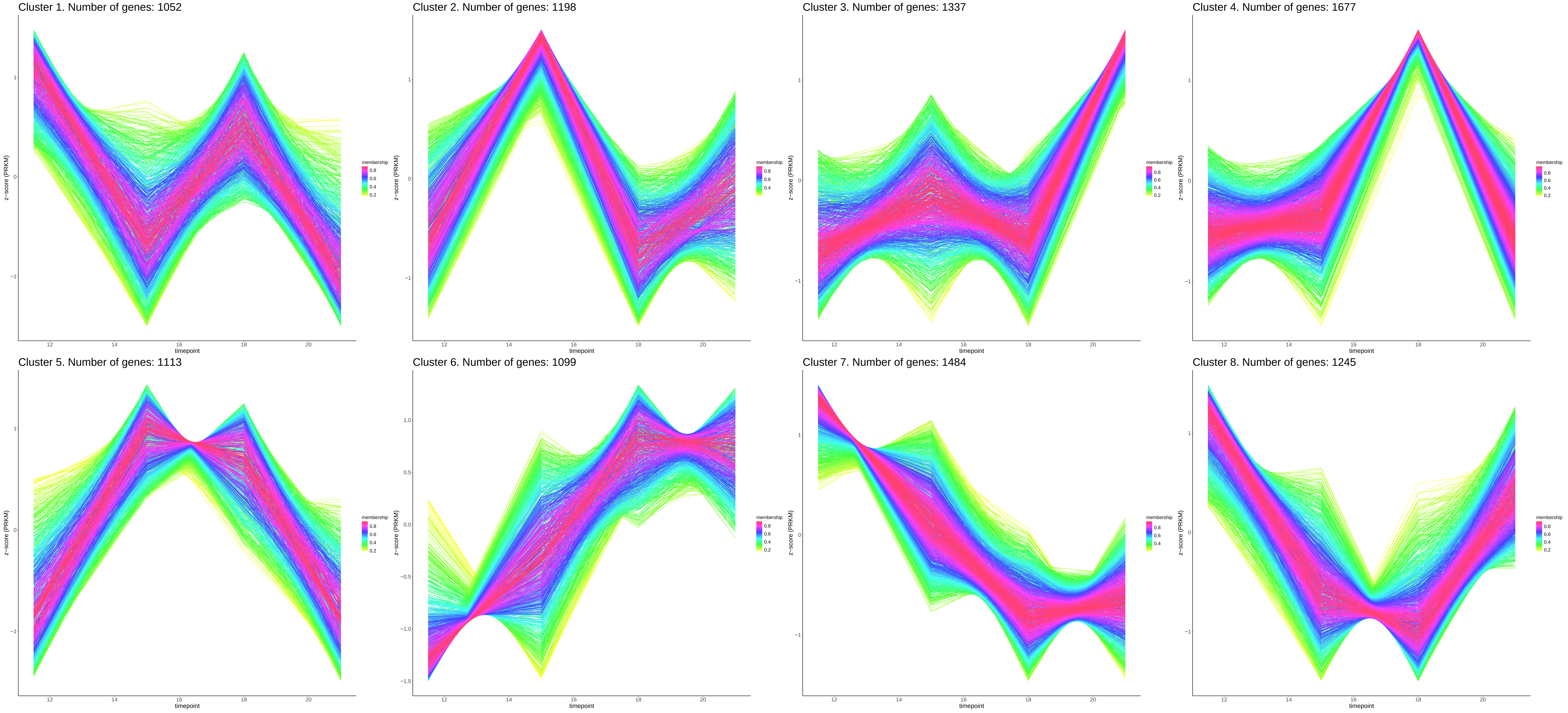

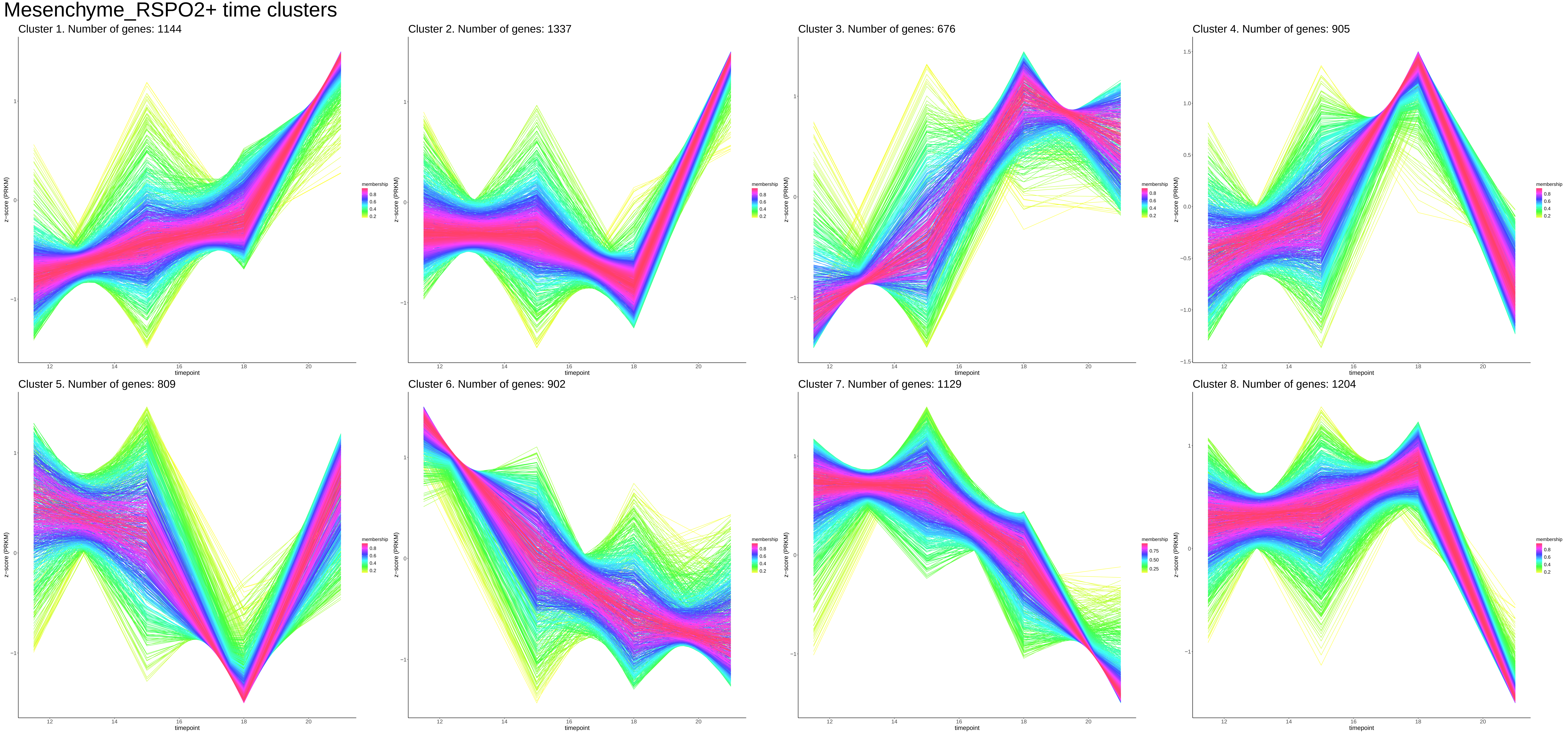
Mesenchyme_RSPO2+ time clusters
Cluster 1. Number of genes: 1144
Cluster 2. Number of genes: 1337
Cluster 3. Number of genes: 676
Cluster 4. Number of genes: 905
Cluster 5. Number of genes: 809
Cluster 6. Number of genes: 902
Cluster 7. Number of genes: 1129
Cluster 8. Number of genes: 1204
z−score (PRKM)
timepoint
12
14
16
18
20
1
0
−1
1.5
1.0
0.5
0.0
−0.5
−1.0
−1.5
membership
0.8
0.6
0.4
0.2
0.75
0.50
0.25

Mesenchyme_SERPINF1−high time clusters

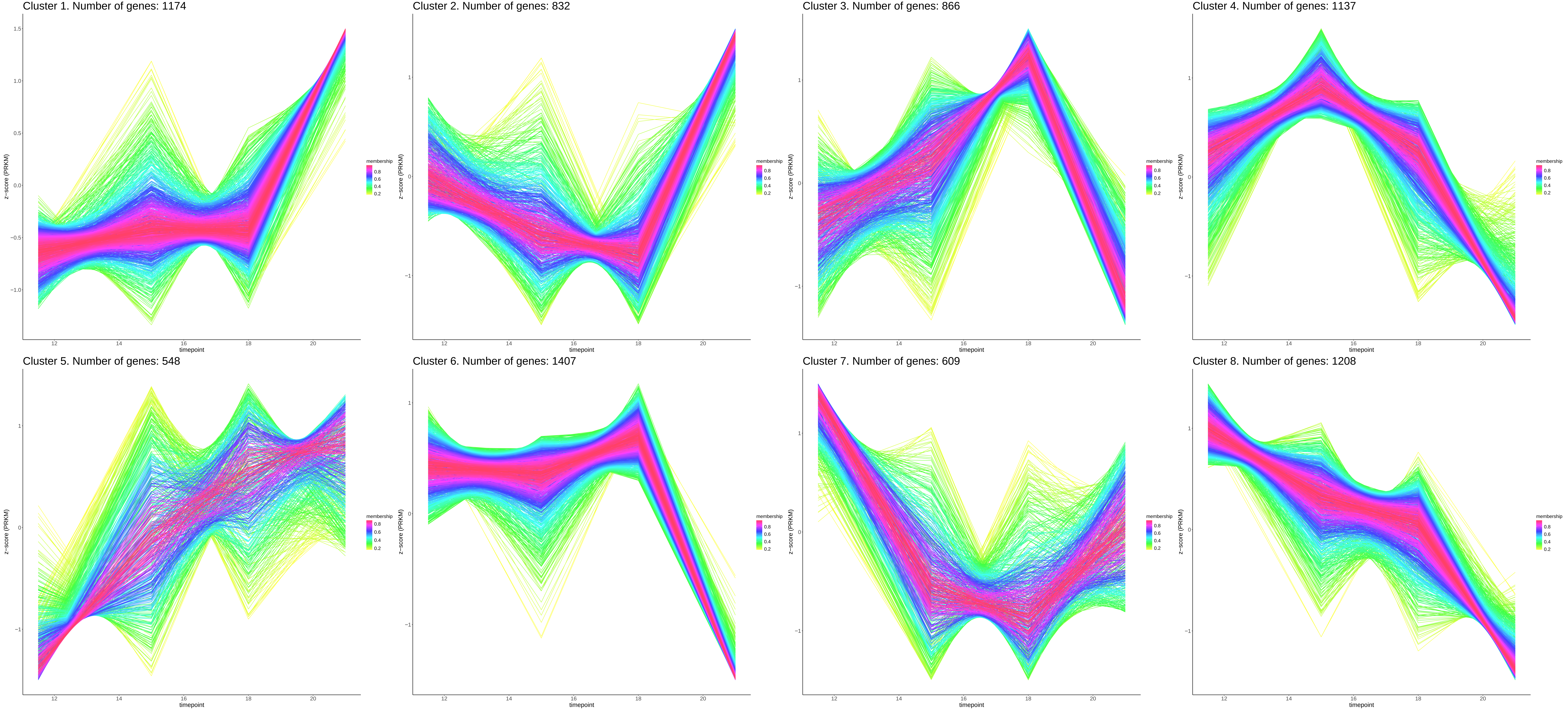

Multiciliated_cell time clusters

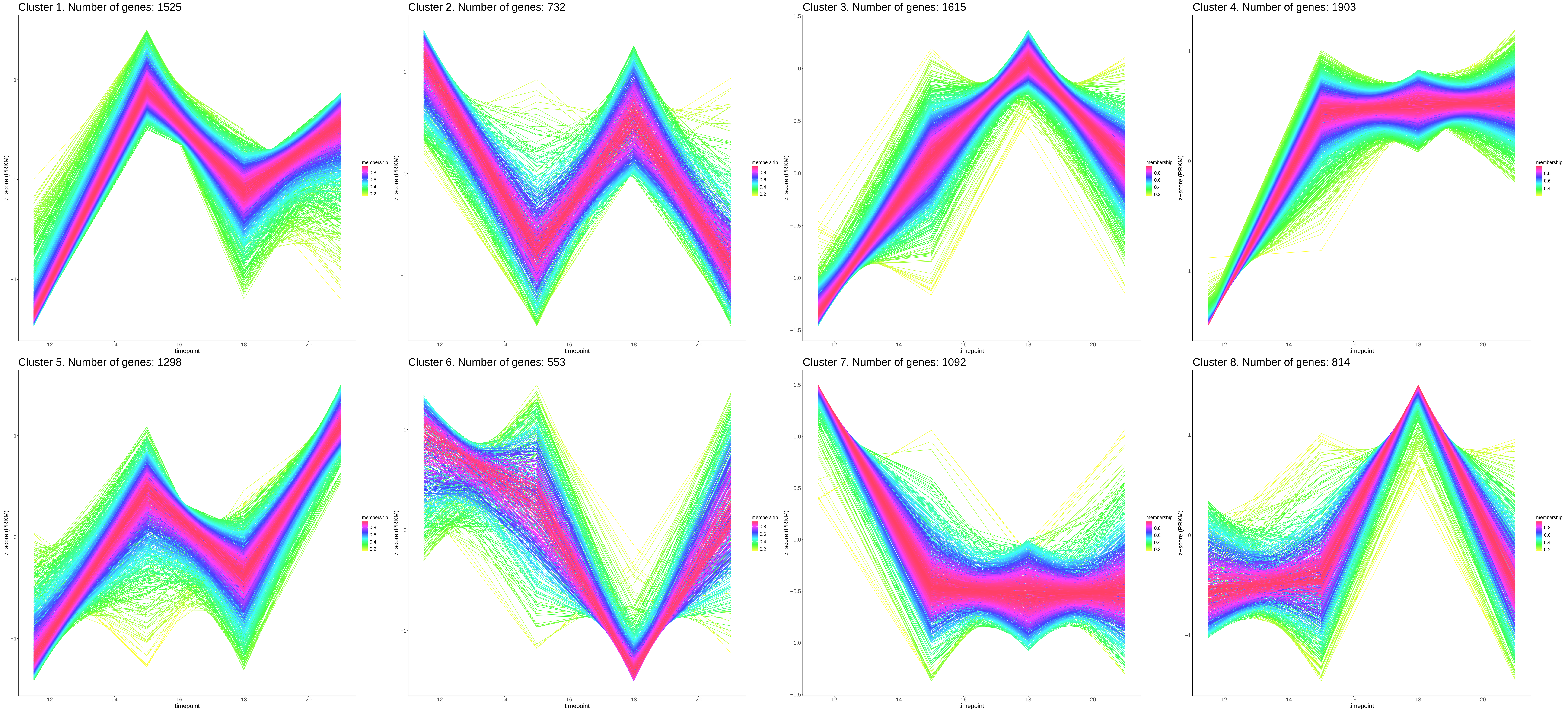

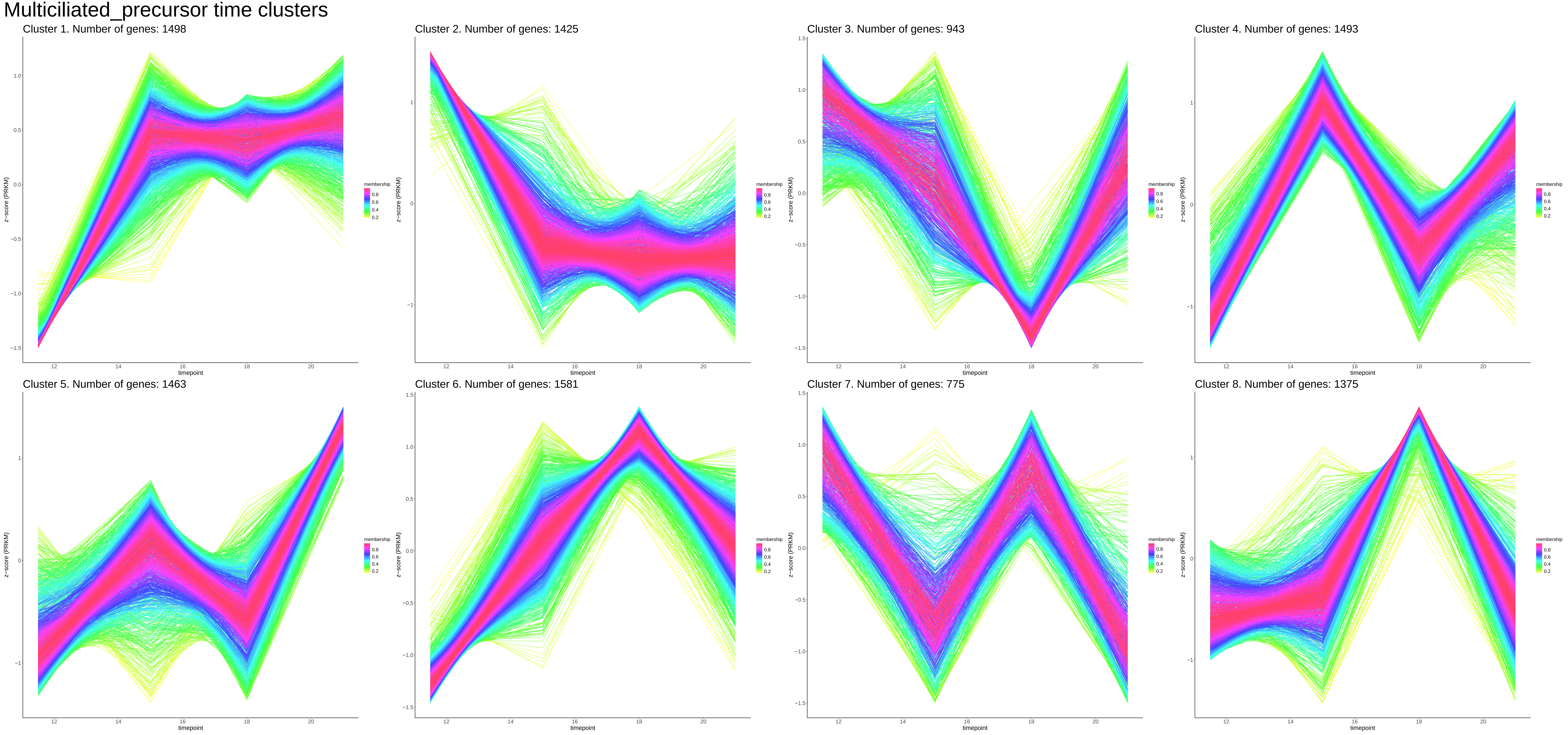
Multiciliated_precursor time clusters
Cluster 1. Number of genes: 1498
Cluster 2. Number of genes: 1425
Cluster 3. Number of genes: 943
Cluster 4. Number of genes: 1493
Cluster 5. Number of genes: 1463
Cluster 6. Number of genes: 1581
Cluster 7. Number of genes: 775
Cluster 8. Number of genes: 1375
z−score (PRKM)
timepoint
membership
0.8
0.6
0.4
0.2

Neuroendocrine time clusters

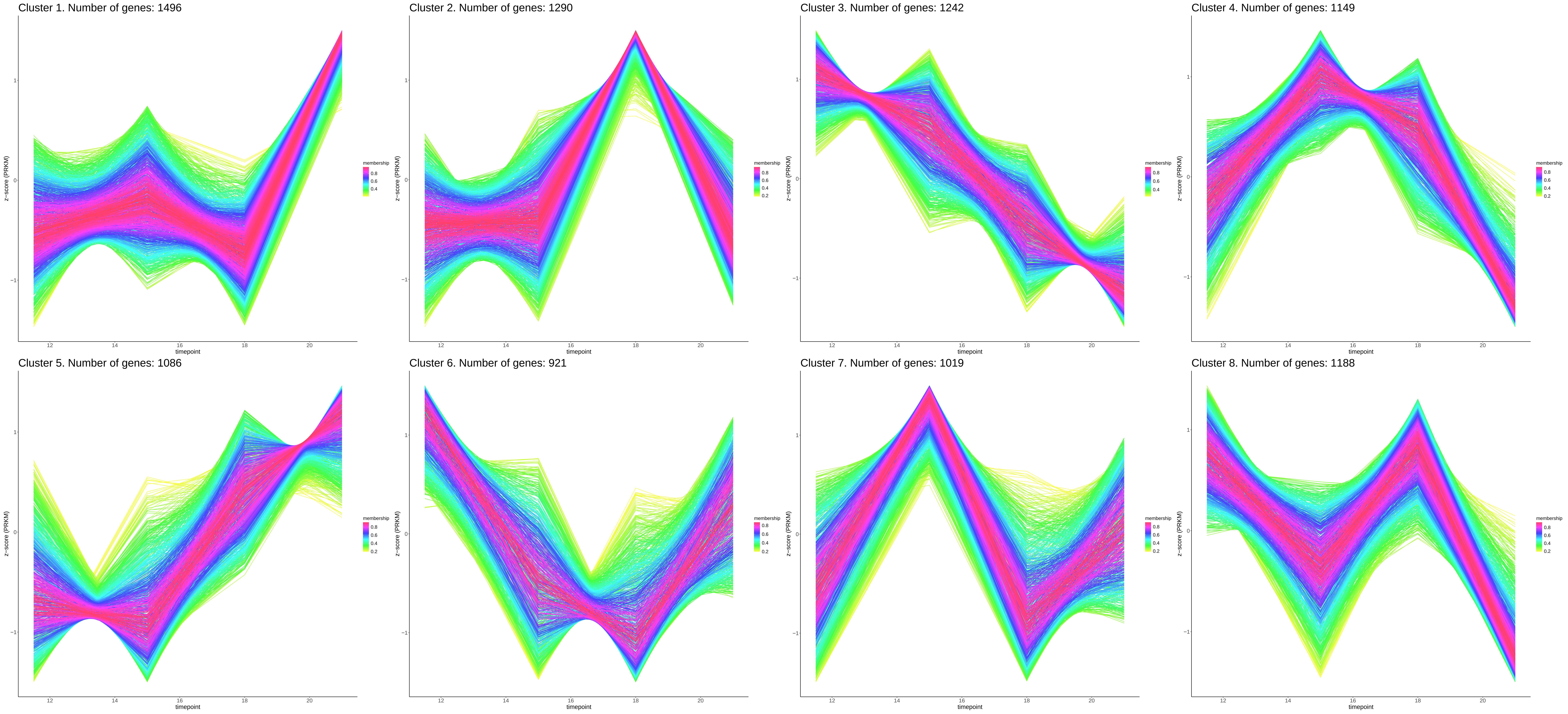

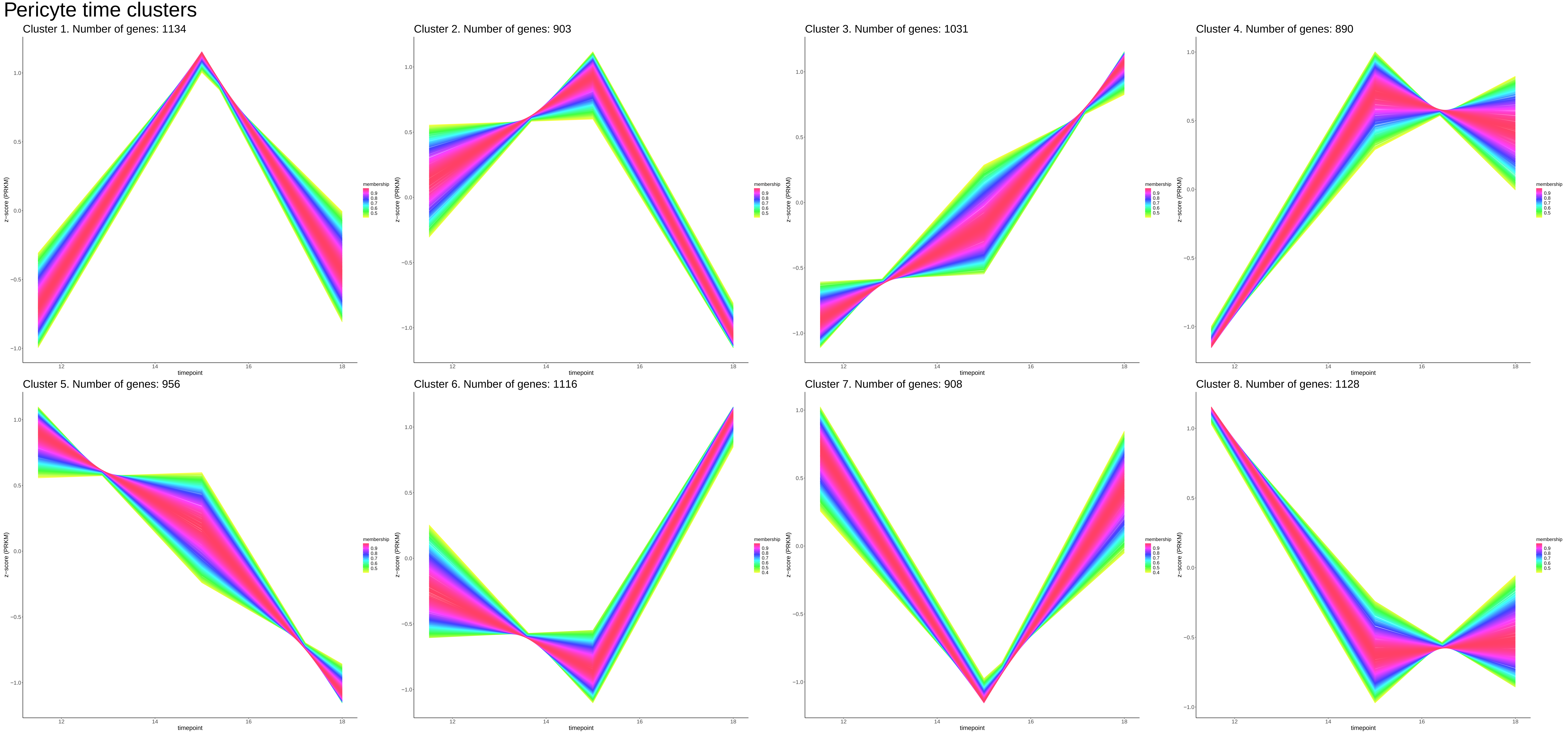
Pericyte time clusters
Cluster 1. Number of genes: 1134
Cluster 2. Number of genes: 903
Cluster 3. Number of genes: 1031
Cluster 4. Number of genes: 890
Cluster 5. Number of genes: 956
Cluster 6. Number of genes: 1116
Cluster 7. Number of genes: 908
Cluster 8. Number of genes: 1128
z−score (PRKM)
timepoint
membership

RBC time clusters

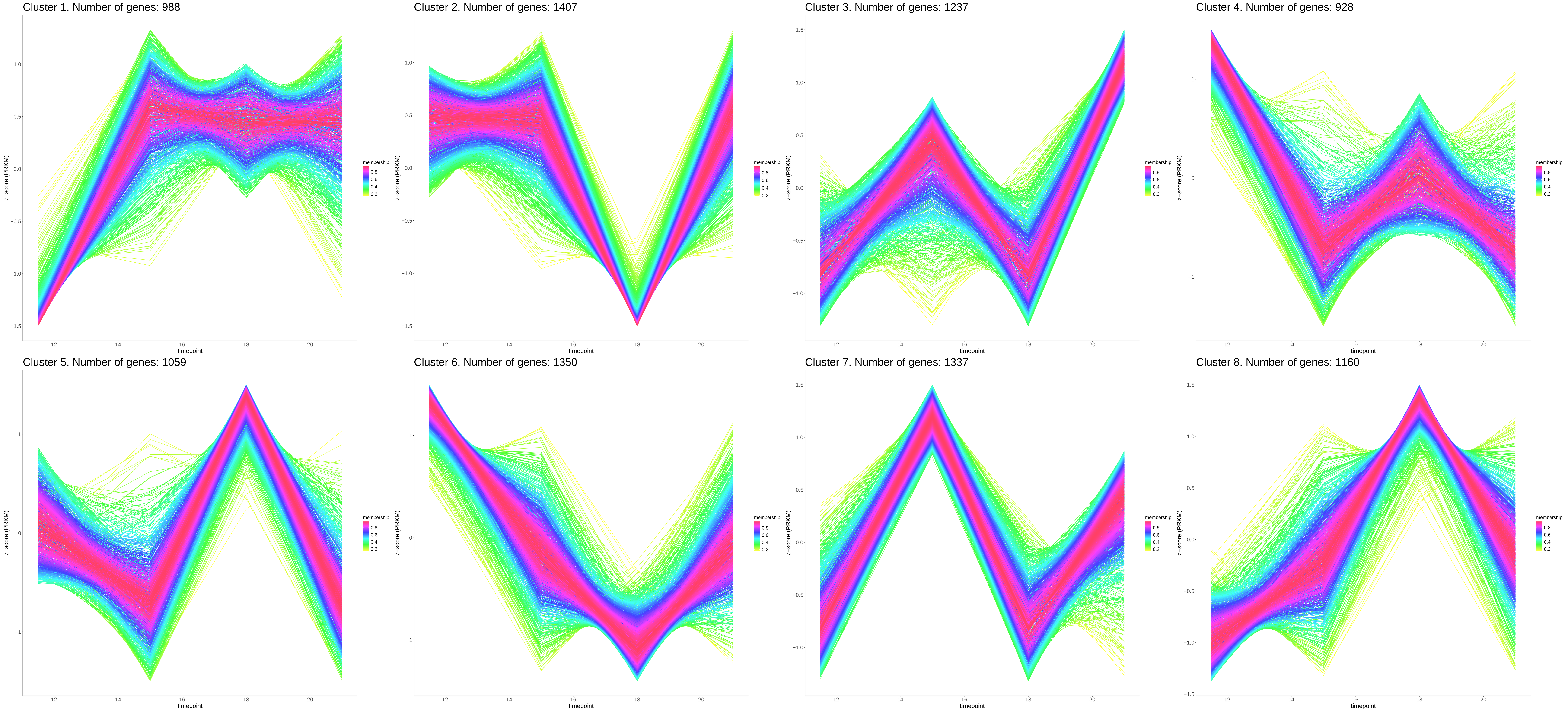

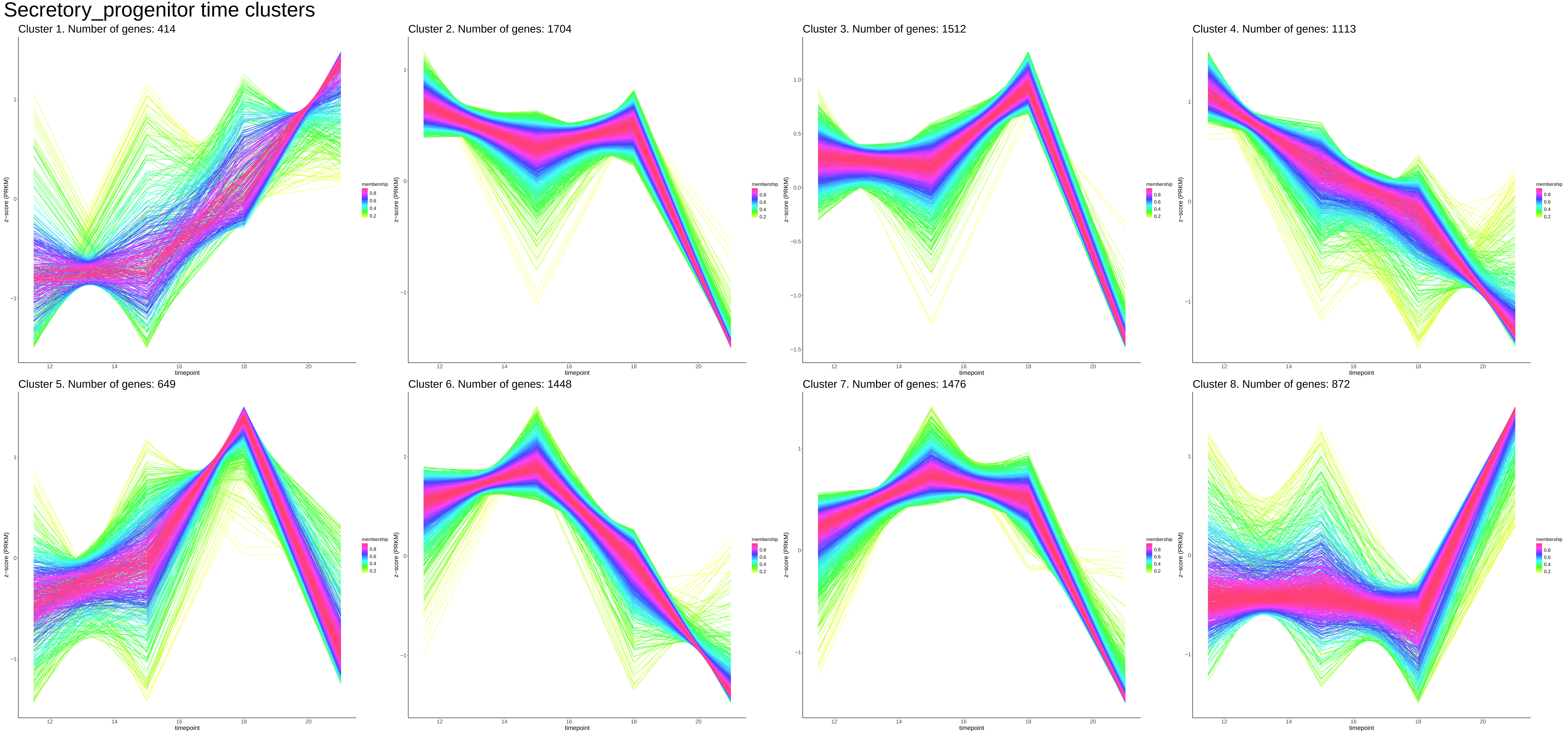
Secretory_progenitor time clusters
Cluster 1. Number of genes: 414
Cluster 2. Number of genes: 1704
Cluster 3. Number of genes: 1512
Cluster 4. Number of genes: 1113
Cluster 5. Number of genes: 649
Cluster 6. Number of genes: 1448
Cluster 7. Number of genes: 1476
Cluster 8. Number of genes: 872
z−score (PRKM)
timepoint
membership
0.8
0.6
0.4
0.2

Submucosal_gland_basal time clusters

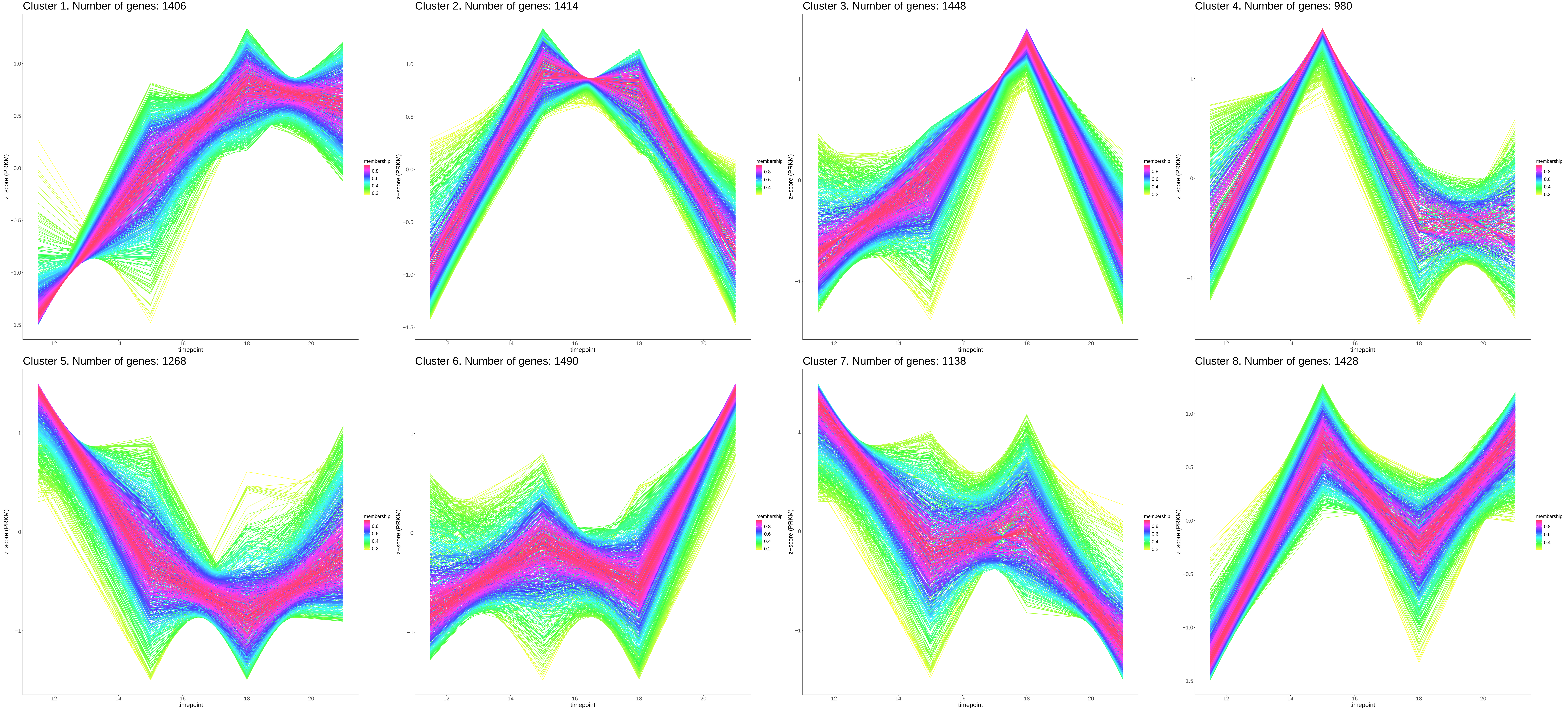

Submucosal_gland time clusters

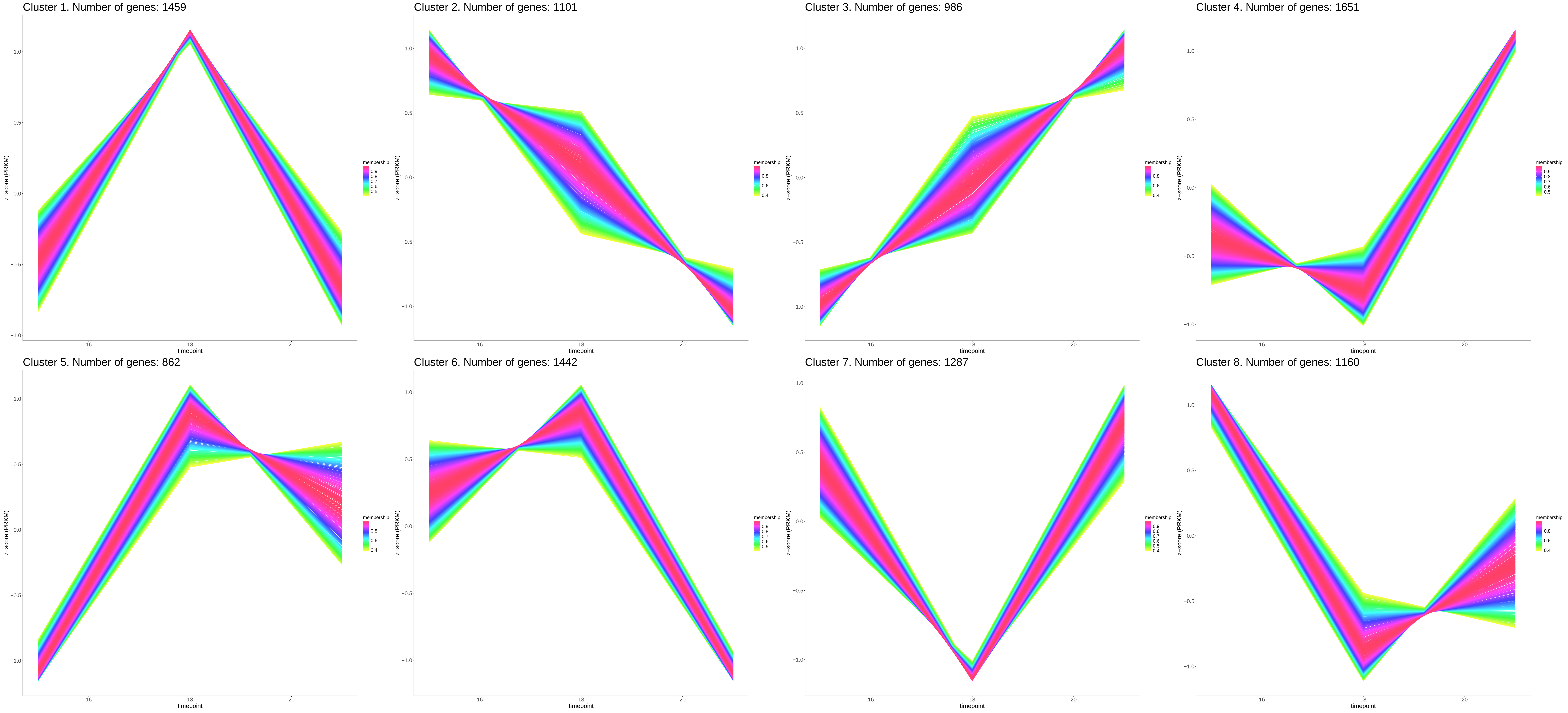

Supplement: lqae180_Supplemental_Files [file lqae180_supplemental_files.zip › FigS4-FetalLungAtlas_TCseqPlots.pdf]
