# Supplementary material for: Mining single-cell data for cell type–disease associations
Source: NAR Genom Bioinform. 2024 Dec 18;6(4):lqae180. doi: 10.1093/nargab/lqae180 (PMC11655289; doi:10.1093/nargab/lqae180)

Adrenocortical_cells time clusters

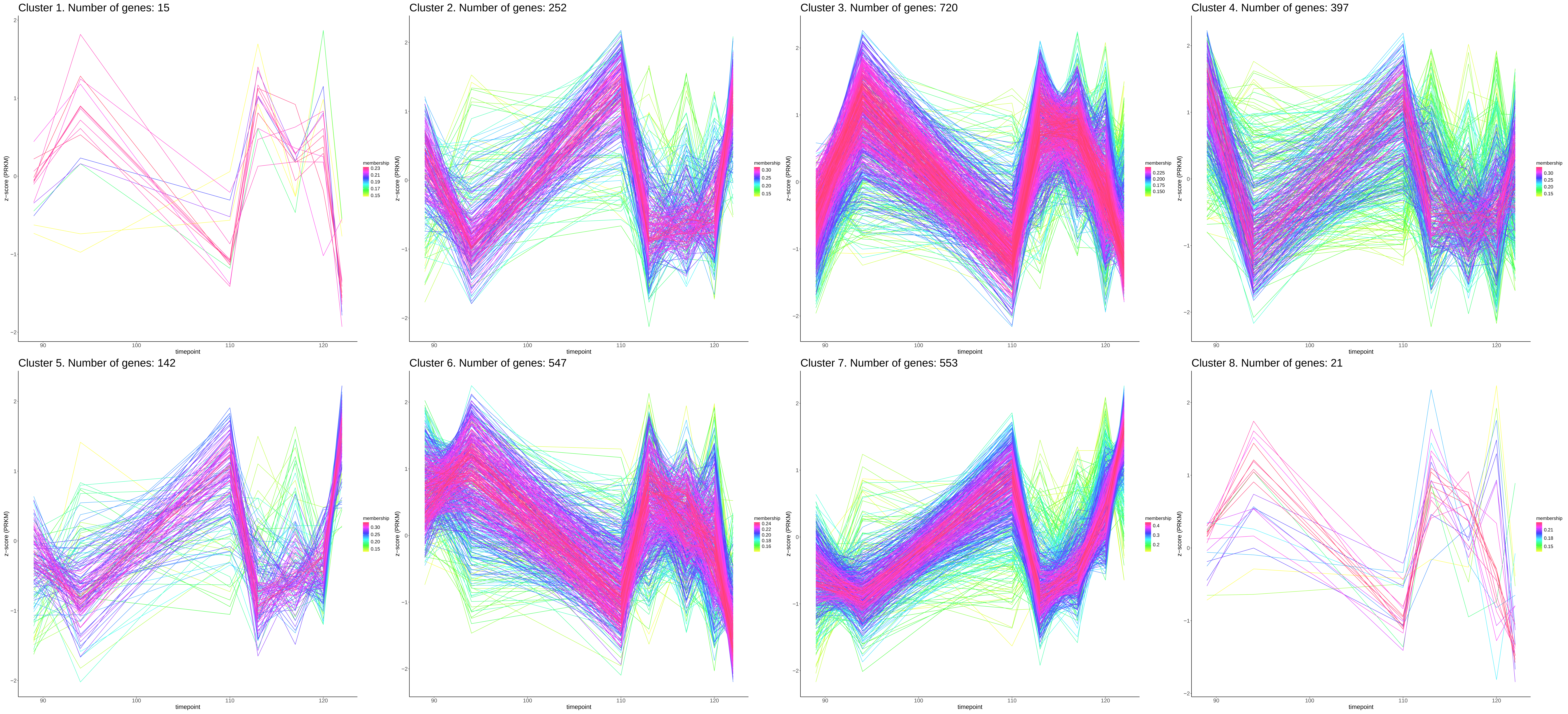

Amacrine_cells time clusters

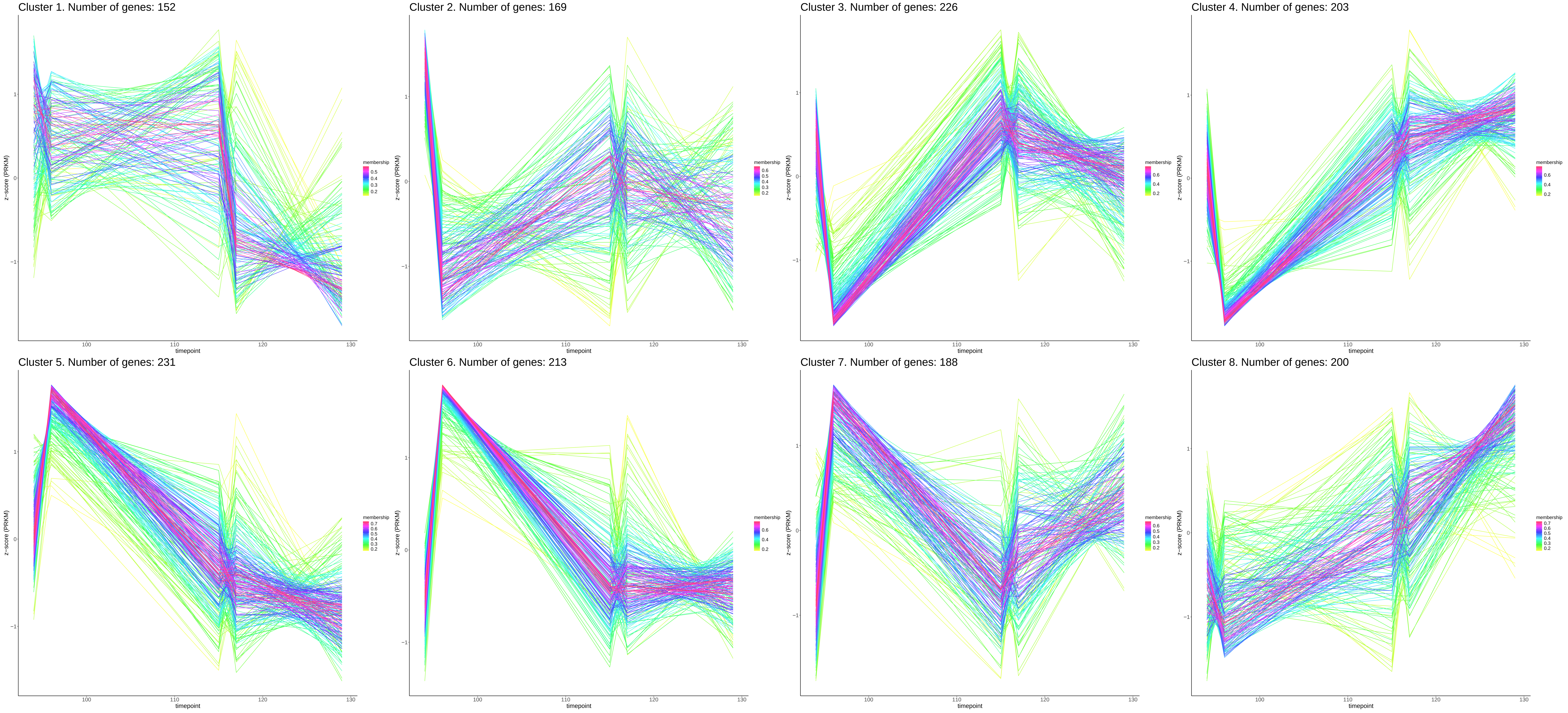

Antigen_presenting_cells time clusters

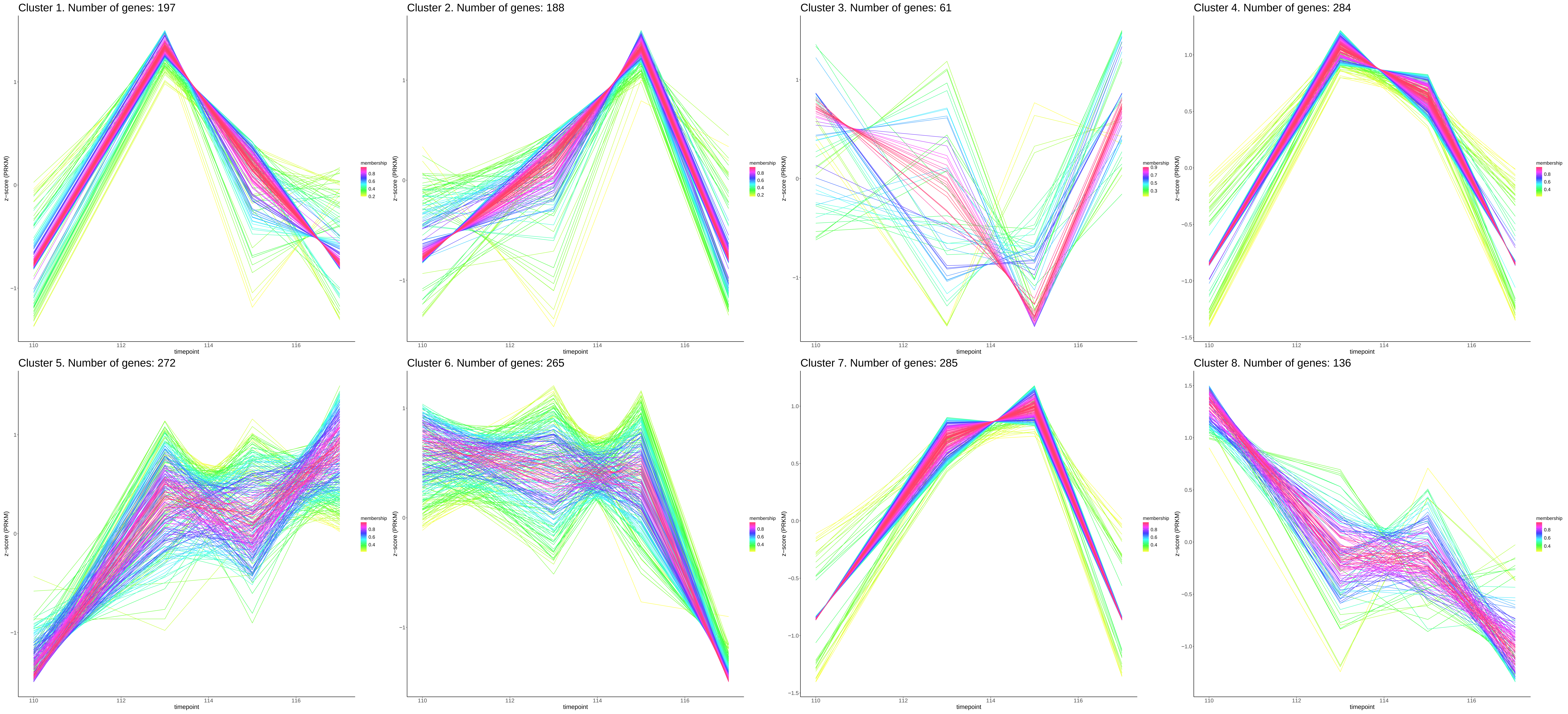

Bipolar_cells time clusters

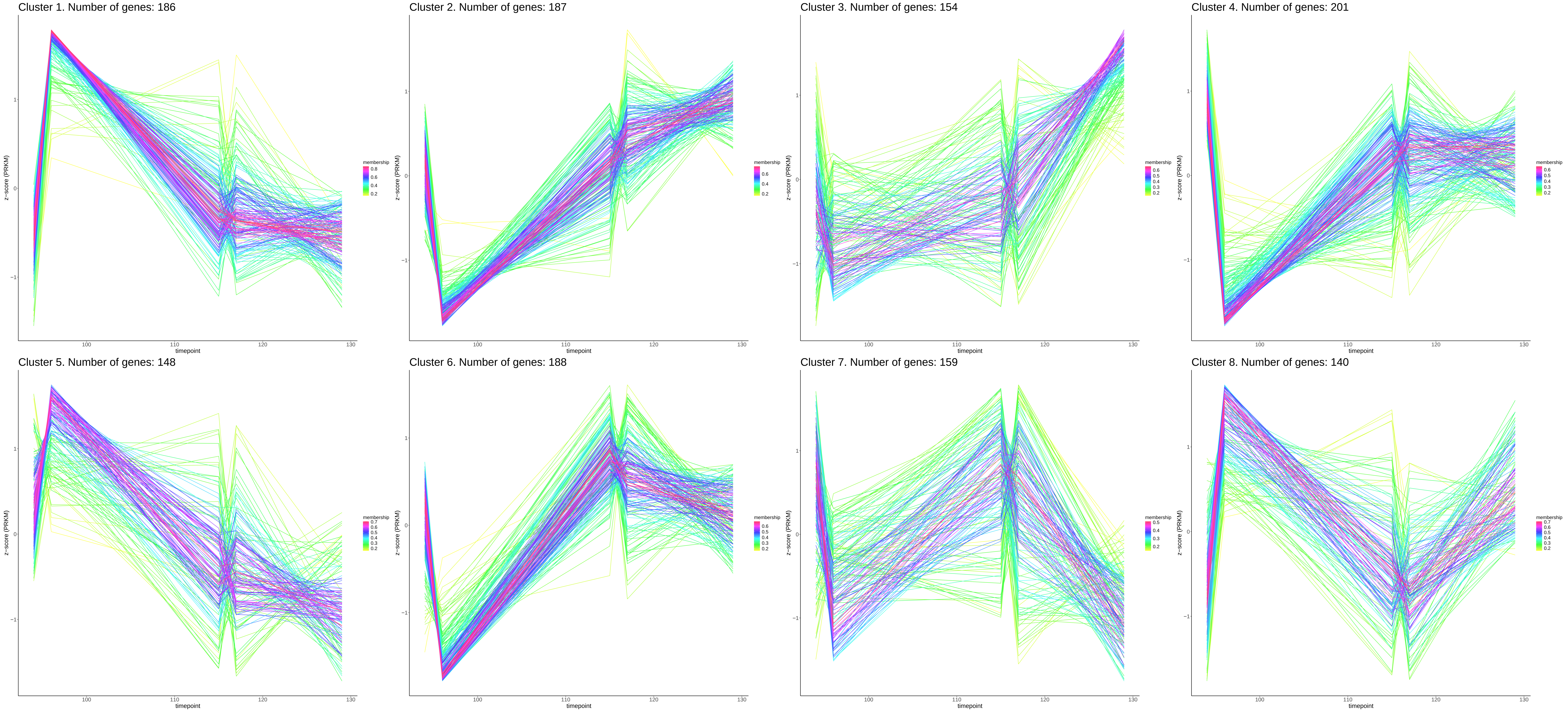

Bronchiolar_and_alveolar_epithelial_cells time clusters

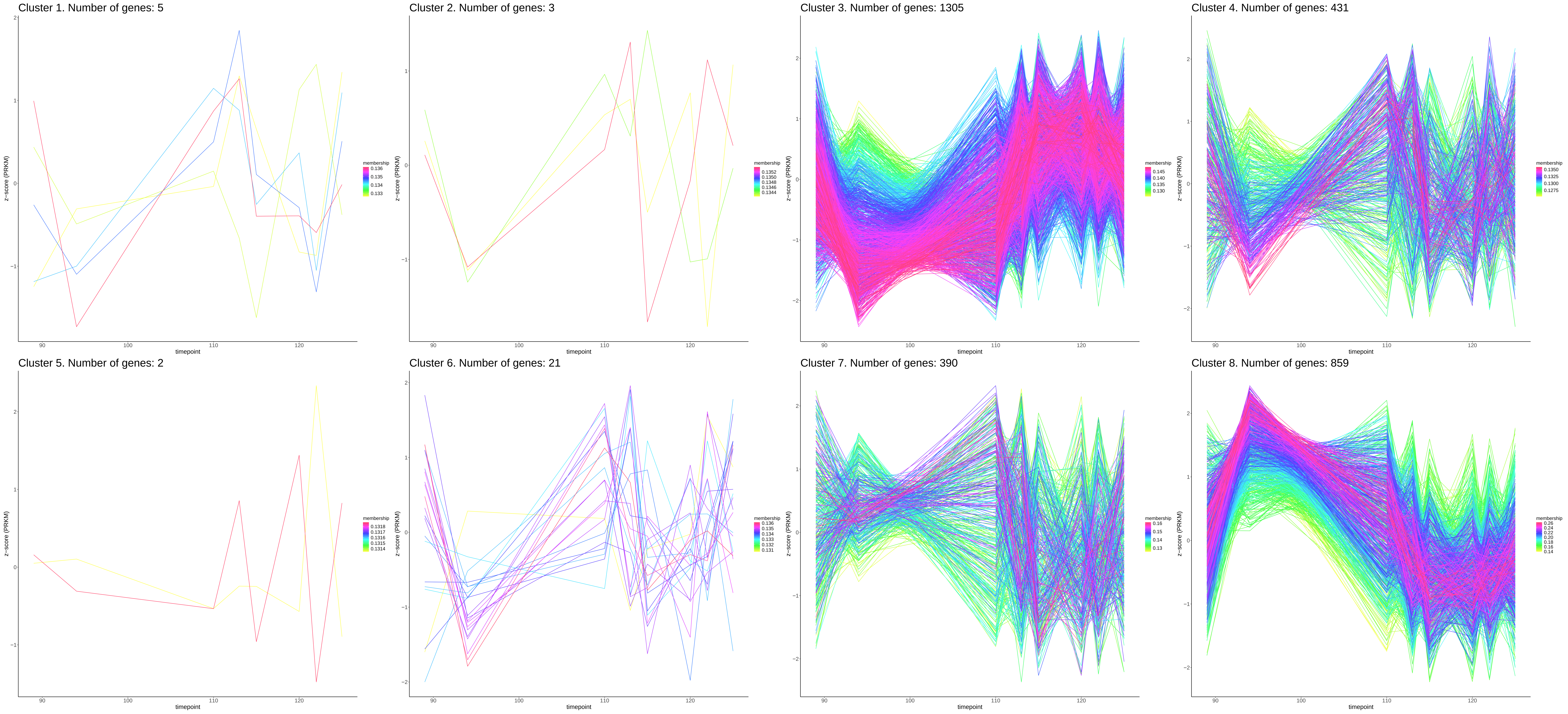

Cardiomyocytes time clusters

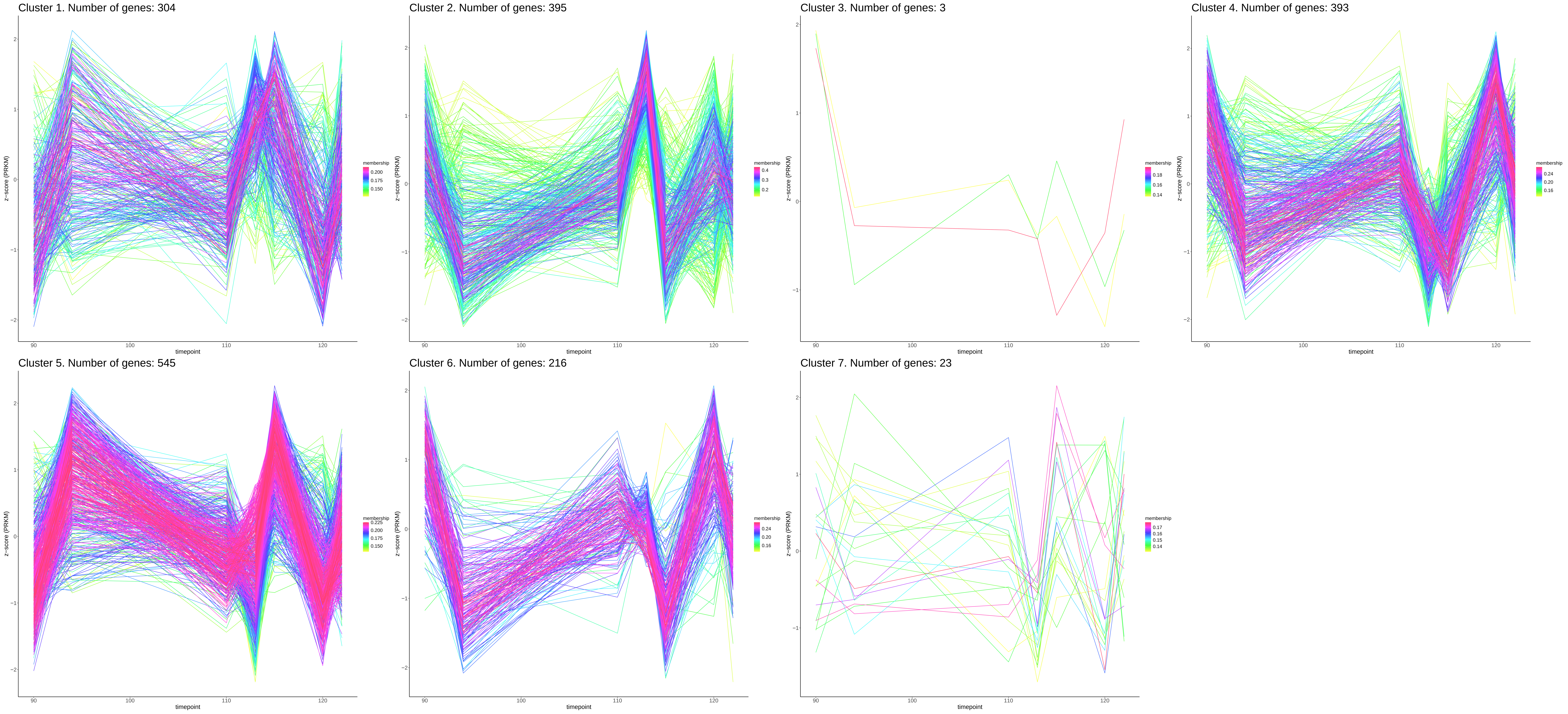

Chromaffin_cells time clusters

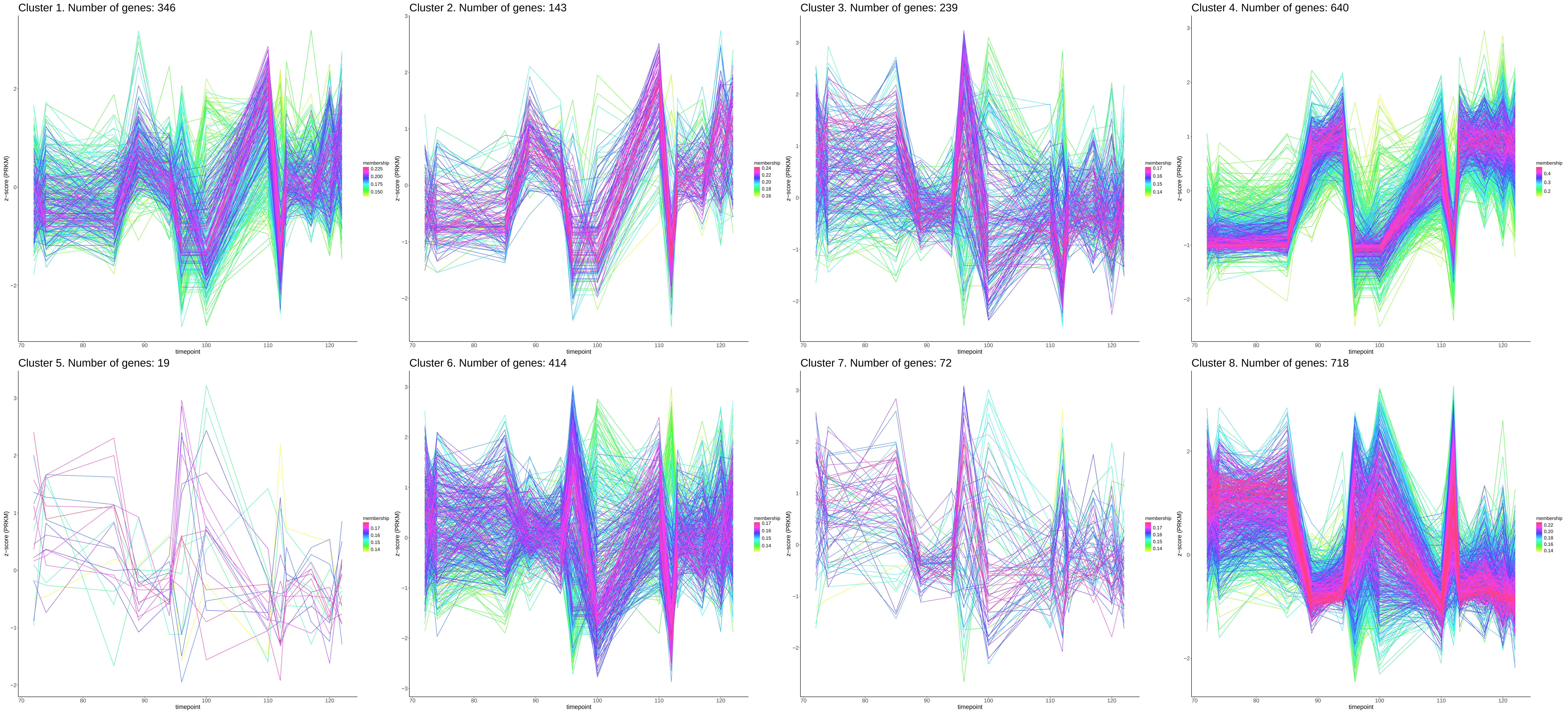

Corneal_and_conjunctival_epithelial_cells time clusters

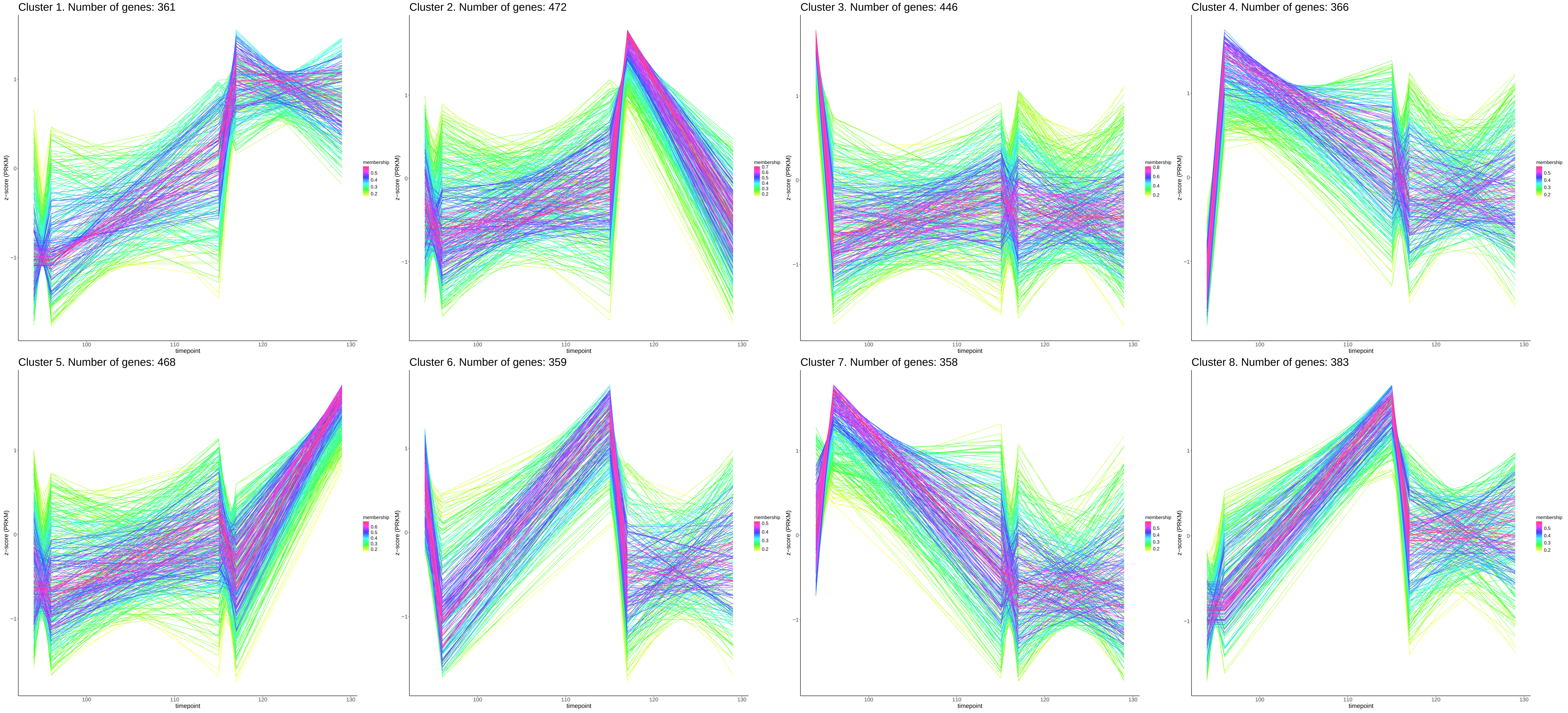

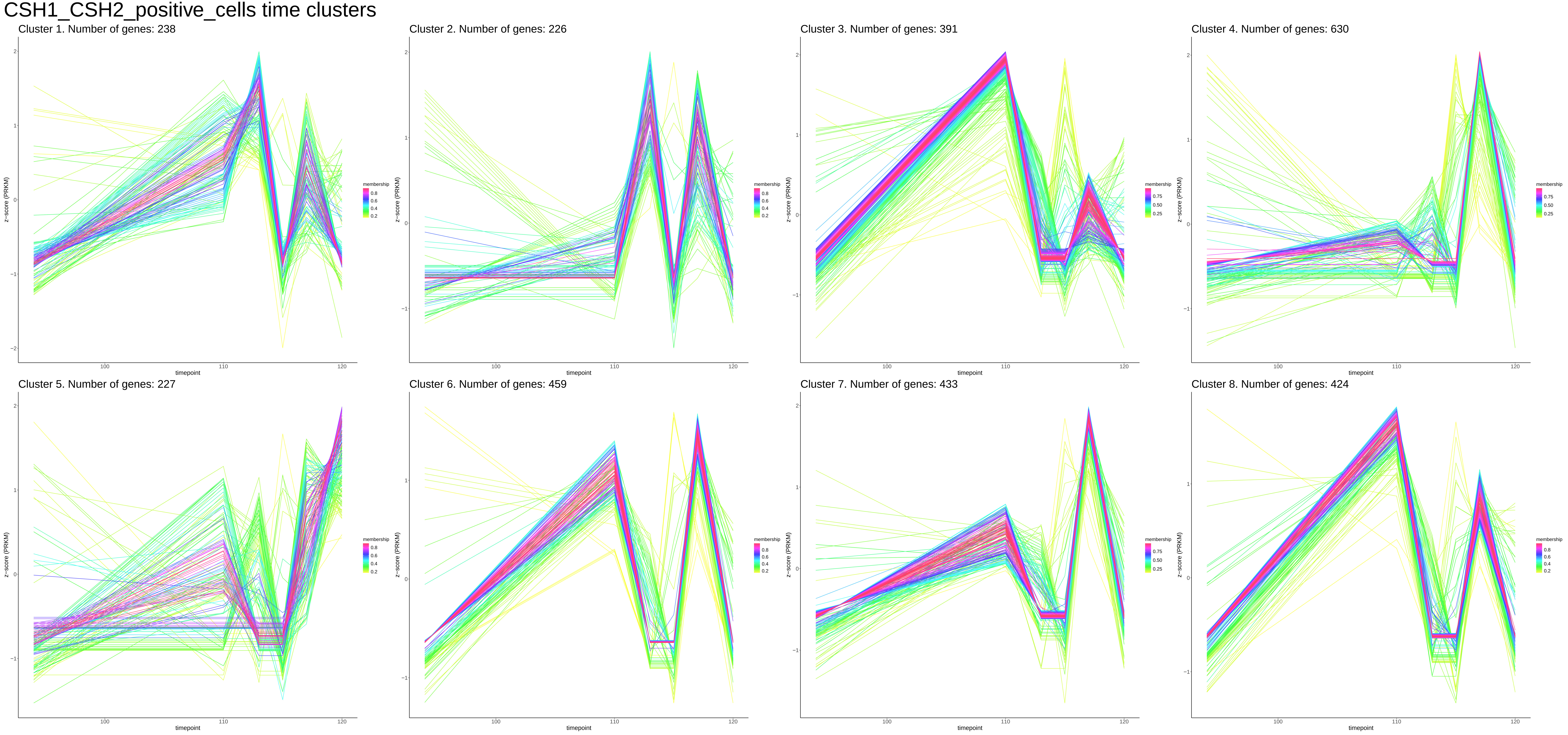
CSH1_CSH2_positive_cells time clusters
Cluster 1. Number of genes: 238
Cluster 2. Number of genes: 226
Cluster 3. Number of genes: 391
Cluster 4. Number of genes: 630
Cluster 5. Number of genes: 227
Cluster 6. Number of genes: 459
Cluster 7. Number of genes: 433
Cluster 8. Number of genes: 424
z-score (PRKM)
timepoint
100
110
120
membership

Ductal_cells time clusters

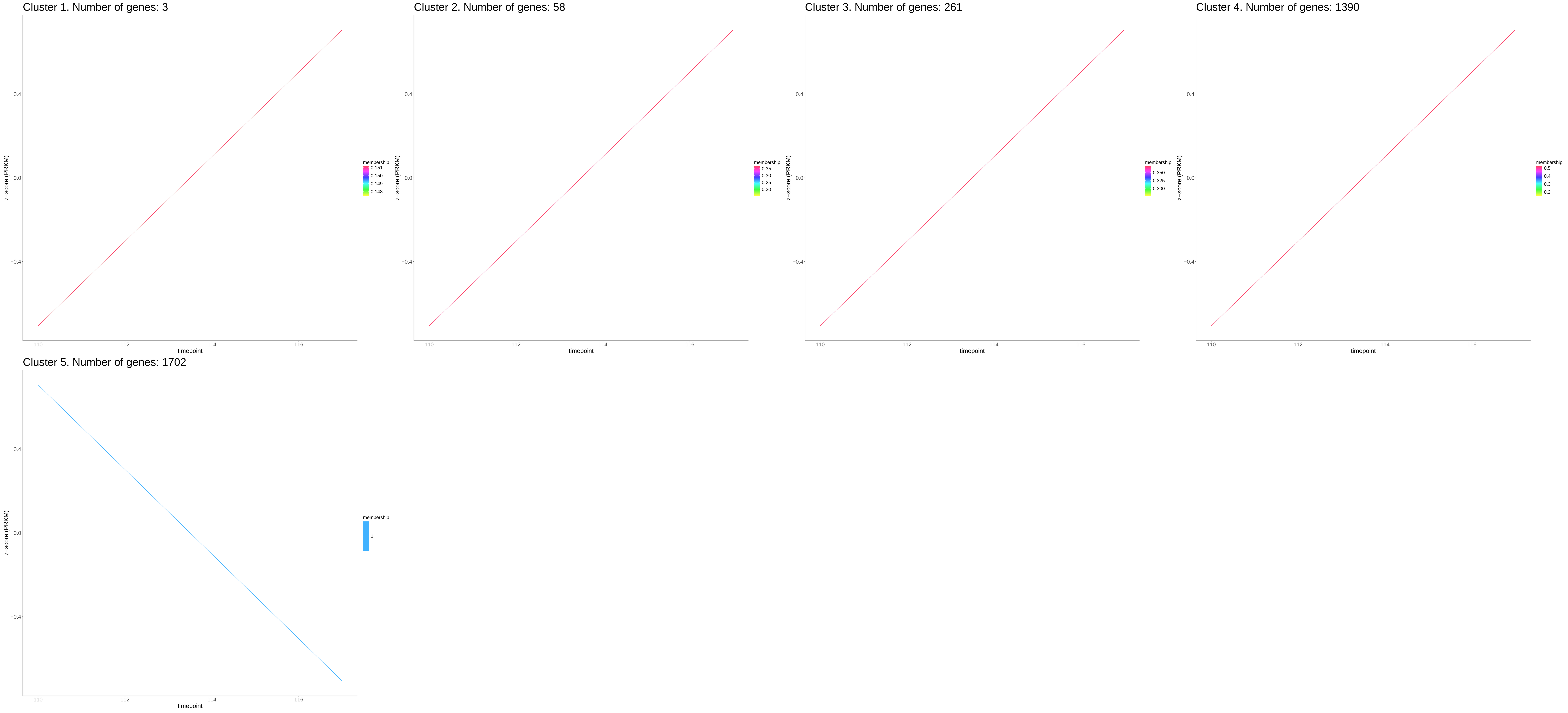

Endocardial_cells time clusters

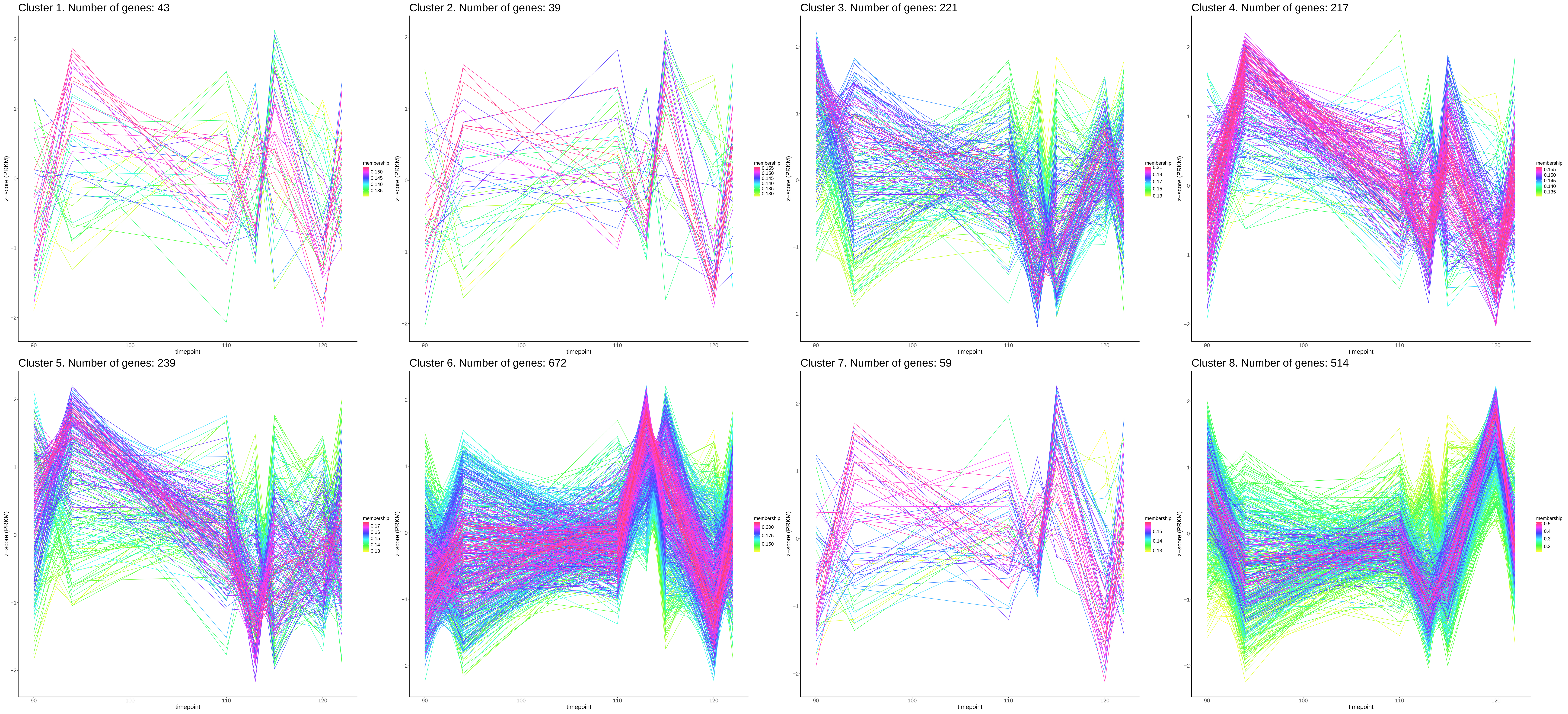

ENS_glia time clusters

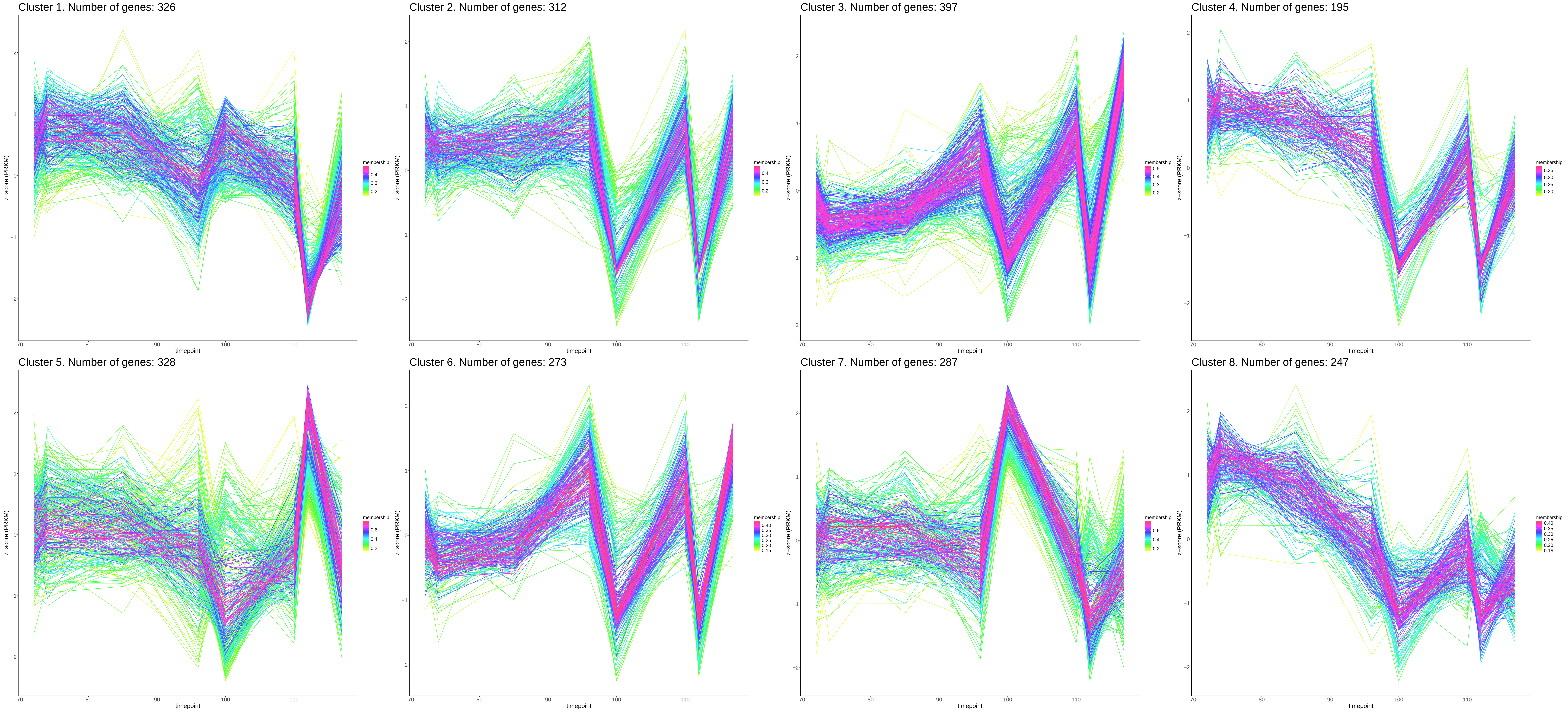

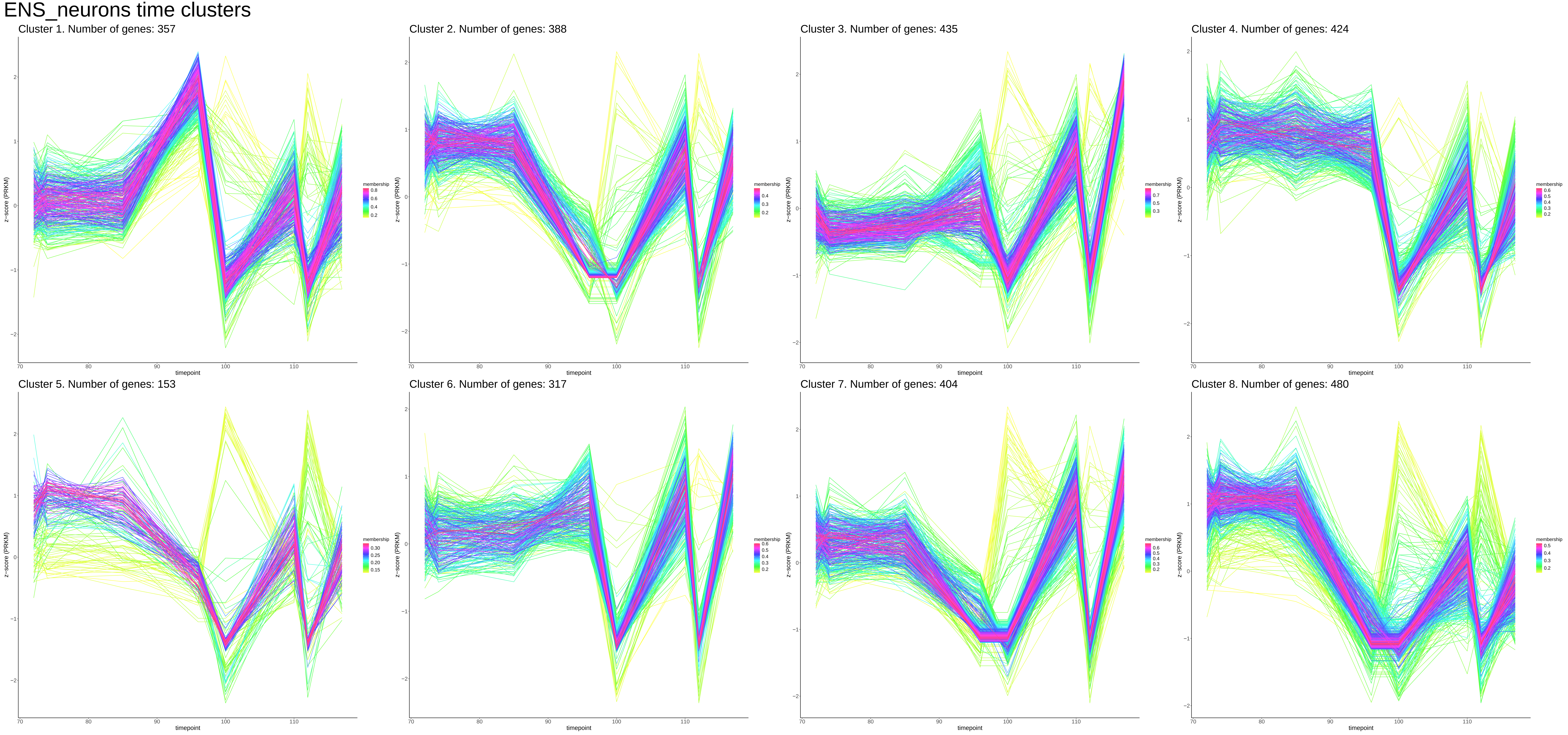
ENS_neurons time clusters
Cluster 1. Number of genes: 357
Cluster 2. Number of genes: 388
Cluster 3. Number of genes: 435
Cluster 4. Number of genes: 424
Cluster 5. Number of genes: 153
Cluster 6. Number of genes: 317
Cluster 7. Number of genes: 404
Cluster 8. Number of genes: 480
z−score (PRKM)
timepoint
membership
70
80
90
100
110

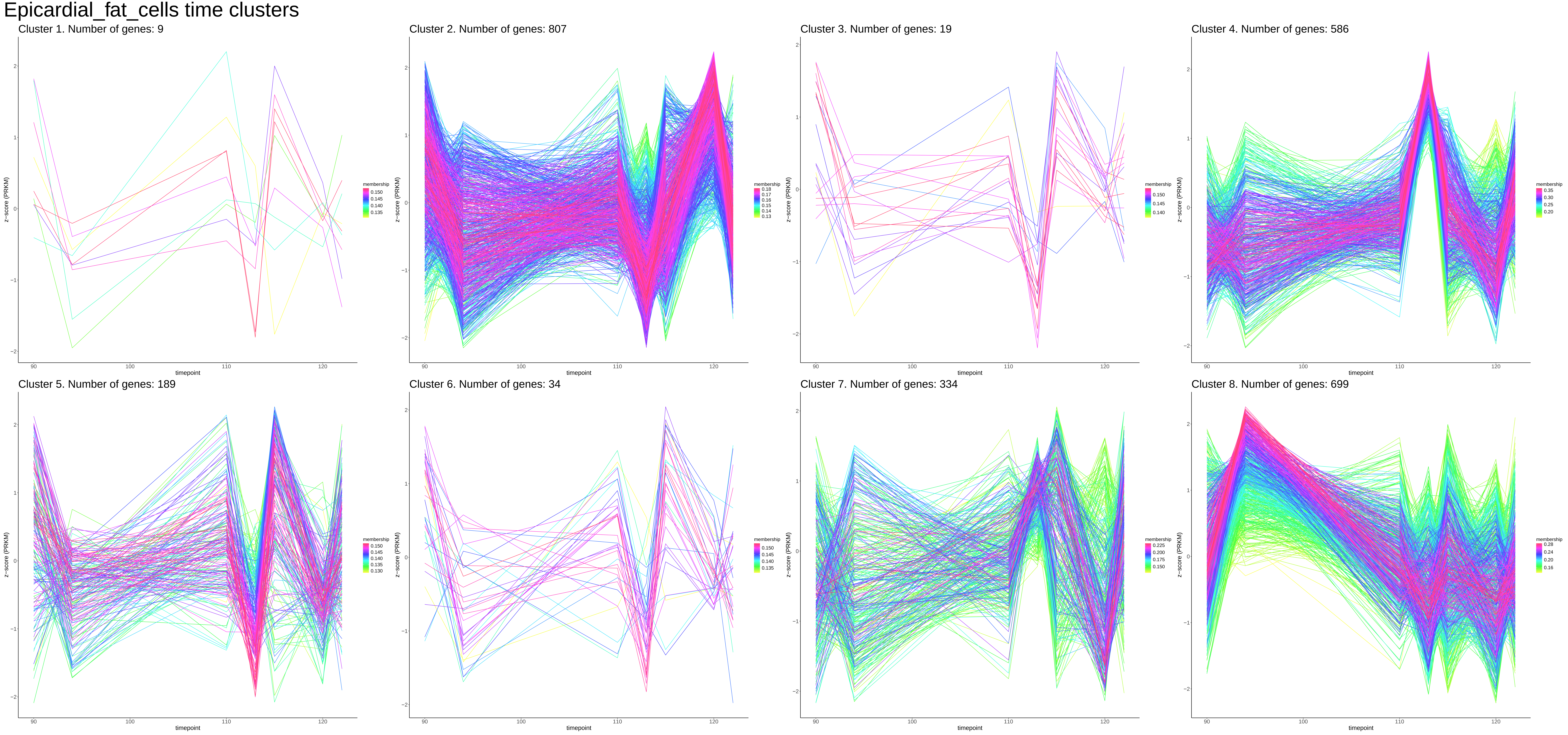
Epicardial_fat_cells time clusters
Cluster 1. Number of genes: 9
Cluster 2. Number of genes: 807
Cluster 3. Number of genes: 19
Cluster 4. Number of genes: 586
Cluster 5. Number of genes: 189
Cluster 6. Number of genes: 34
Cluster 7. Number of genes: 334
Cluster 8. Number of genes: 699
z-score (PRKM)
timepoint
membership

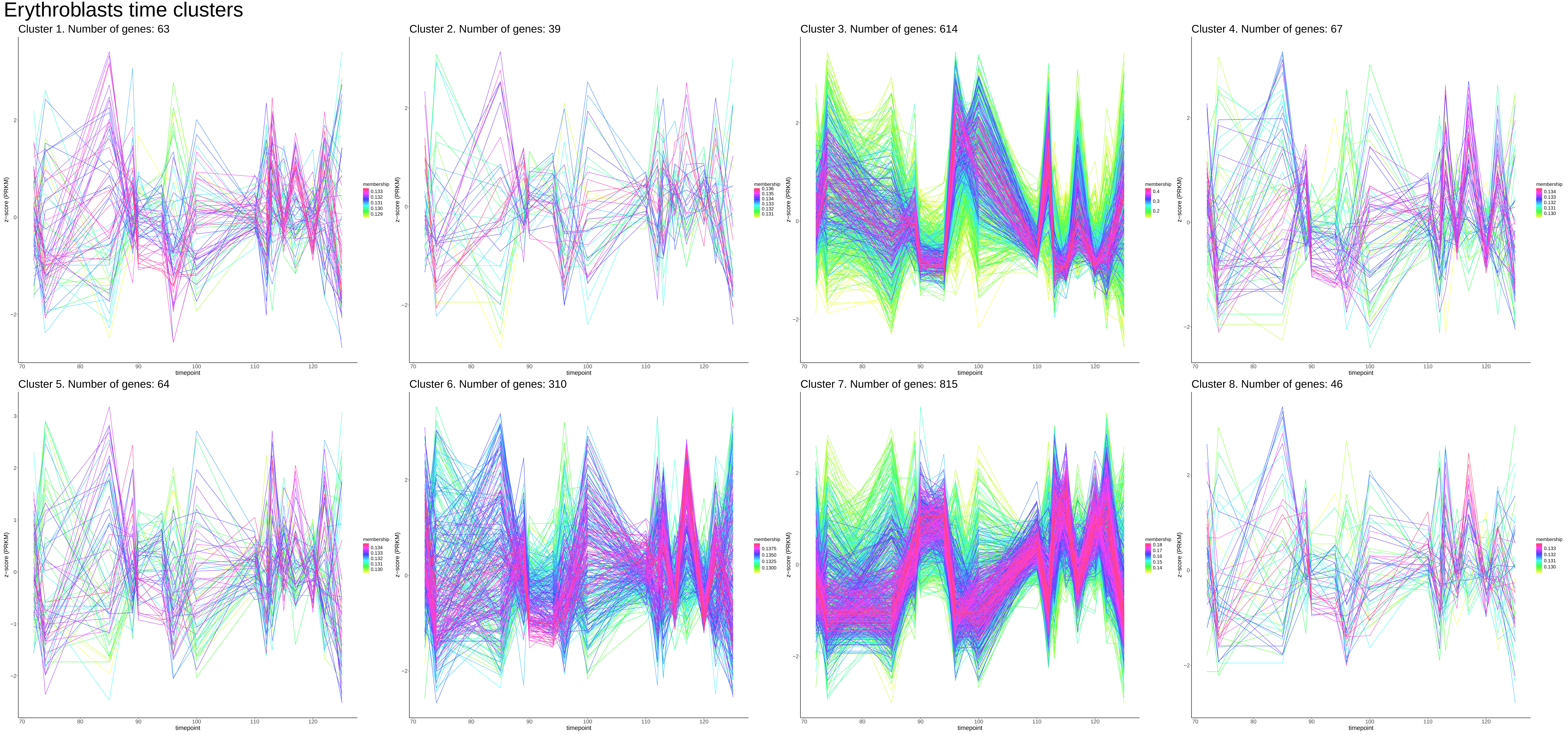

Erythroblasts time clusters
Cluster 1. Number of genes: 63
Cluster 2. Number of genes: 39
Cluster 3. Number of genes: 614
Cluster 4. Number of genes: 67
Cluster 5. Number of genes: 64
Cluster 6. Number of genes: 310
Cluster 7. Number of genes: 815
Cluster 8. Number of genes: 46
z−score (PRKM)
timepoint
membership

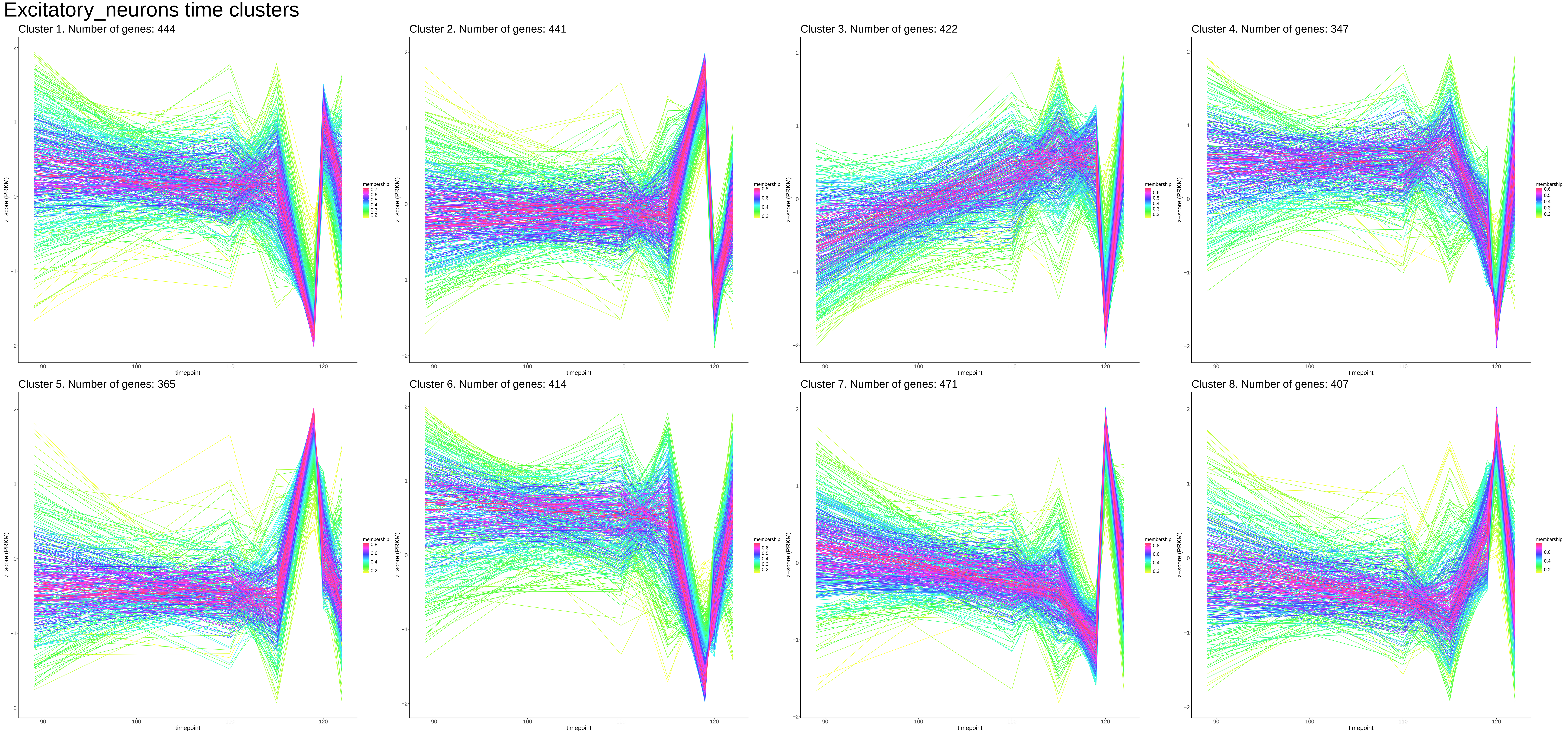
Excitatory_neurons time clusters
Cluster 1. Number of genes: 444
Cluster 2. Number of genes: 441
Cluster 3. Number of genes: 422
Cluster 4. Number of genes: 347
Cluster 5. Number of genes: 365
Cluster 6. Number of genes: 414
Cluster 7. Number of genes: 471
Cluster 8. Number of genes: 407
z−score (PRKM)
timepoint
membership

Extravillous_trophoblasts time clusters

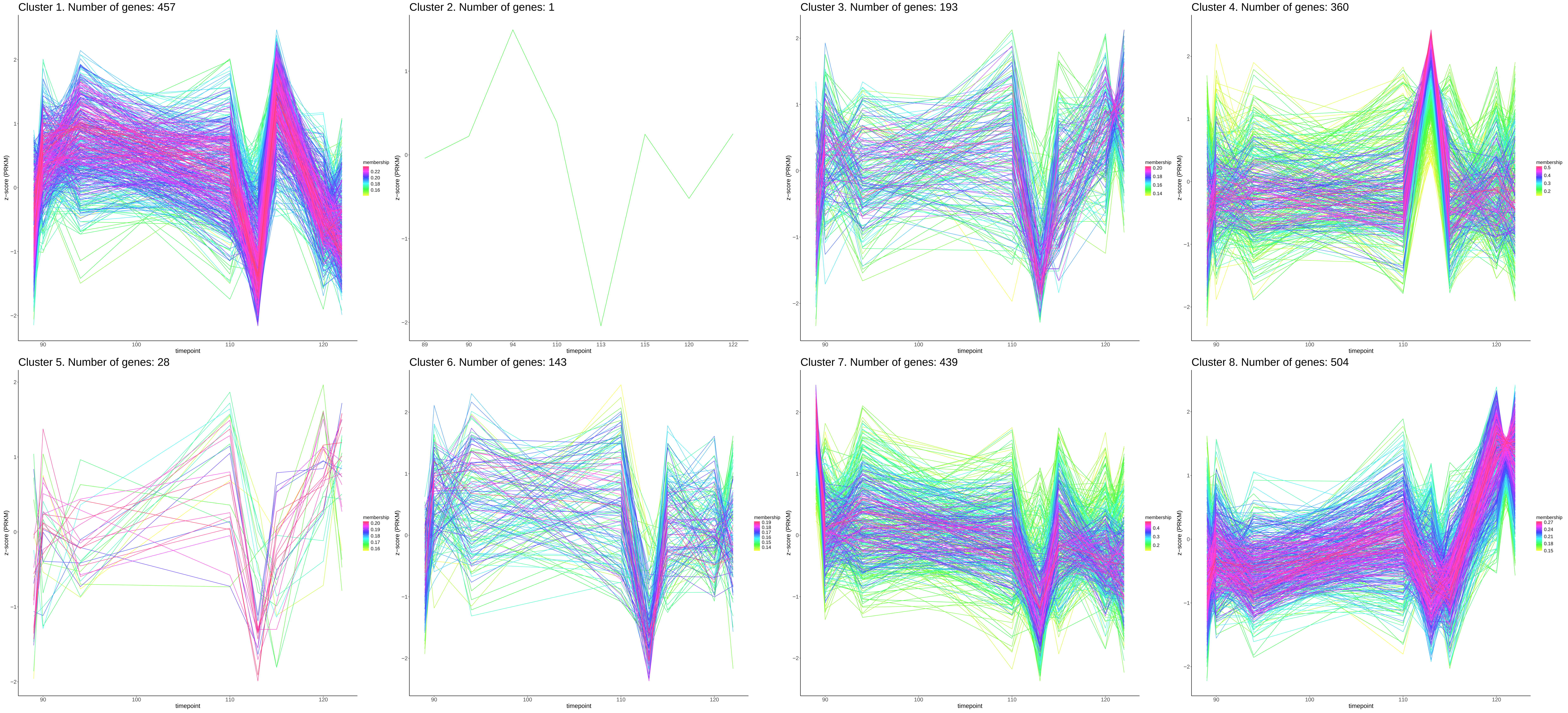

Ganglion_cells time clusters

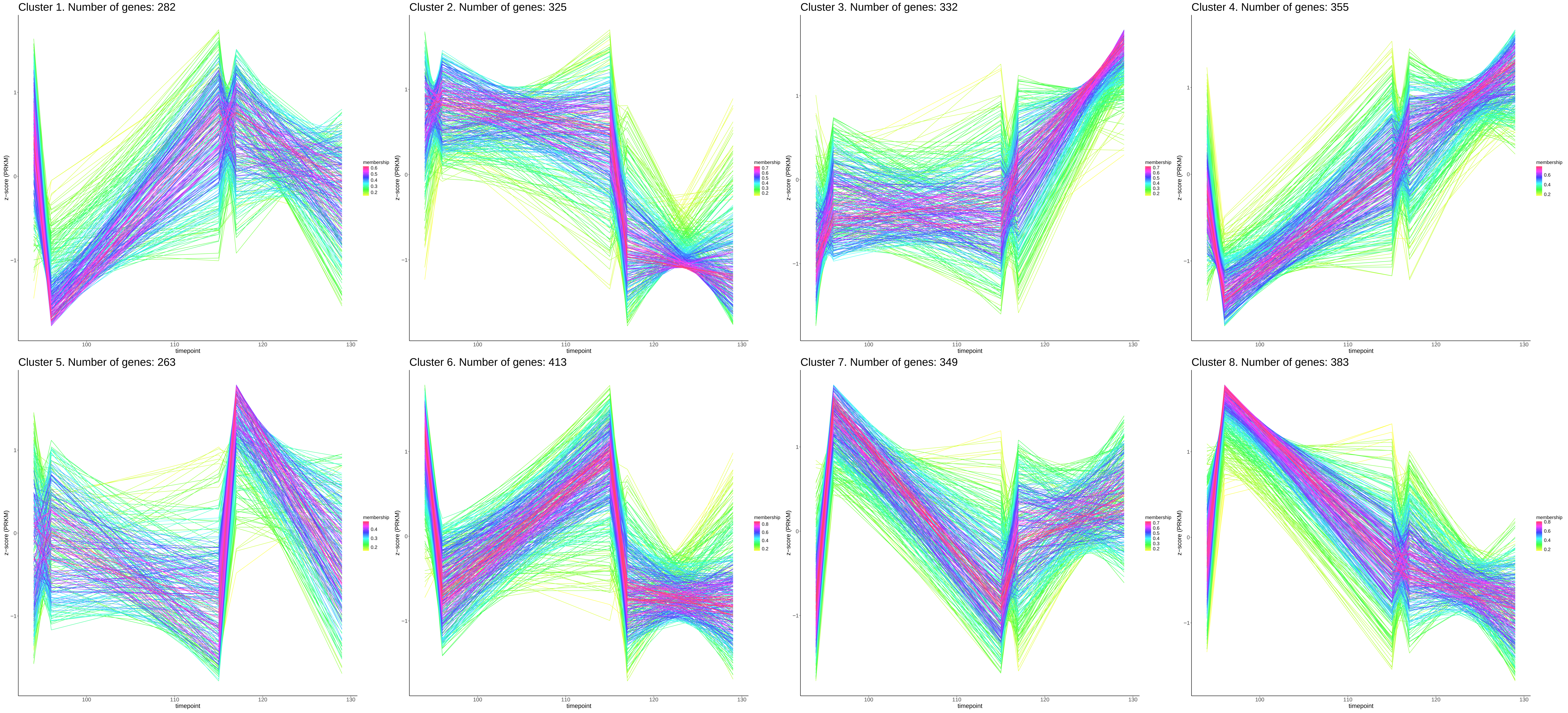

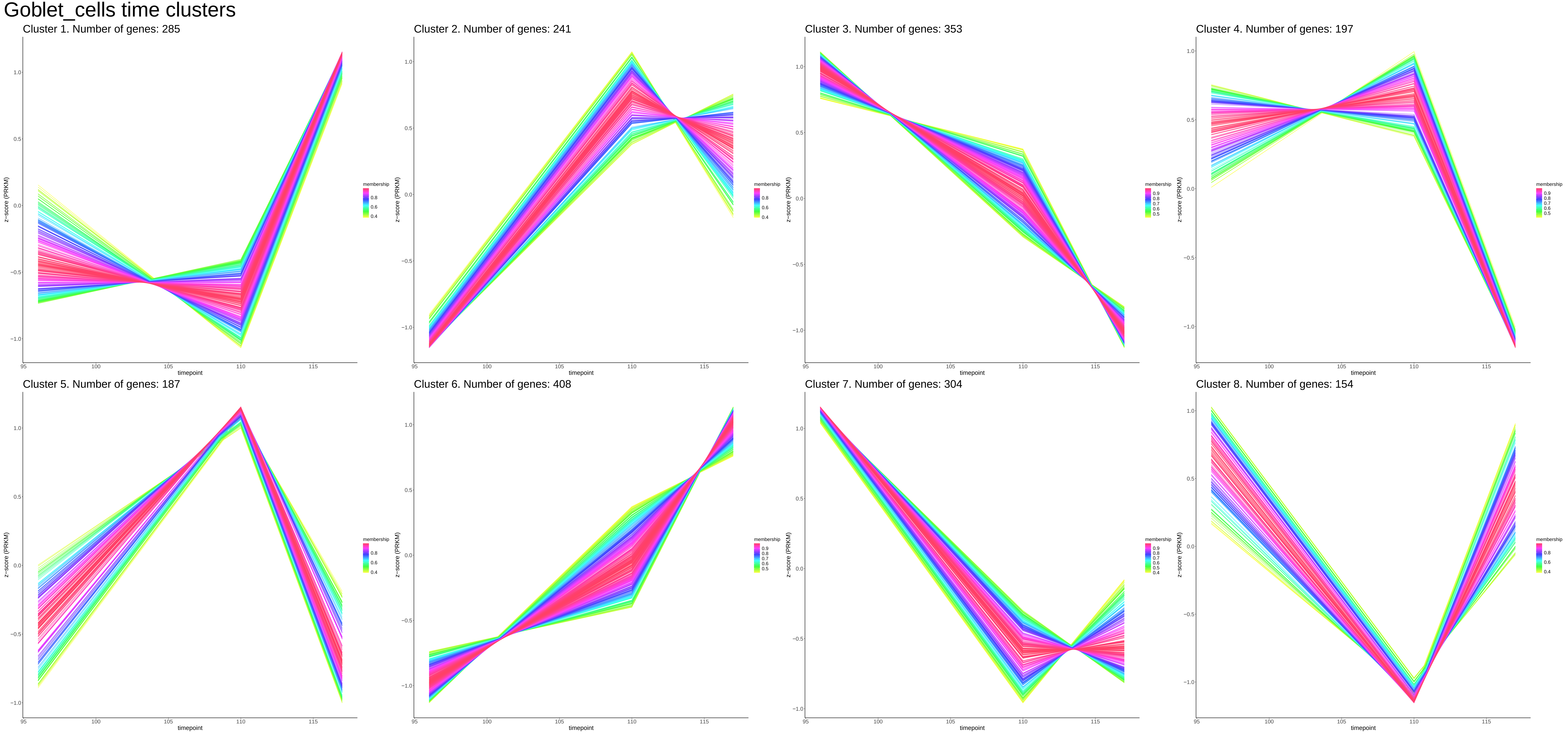
Goblet_cells time clusters
Cluster 1. Number of genes: 285
Cluster 2. Number of genes: 241
Cluster 3. Number of genes: 353
Cluster 4. Number of genes: 197
Cluster 5. Number of genes: 187
Cluster 6. Number of genes: 408
Cluster 7. Number of genes: 304
Cluster 8. Number of genes: 154
z−score (PRKM)
timepoint
membership
95
100
105
110
115

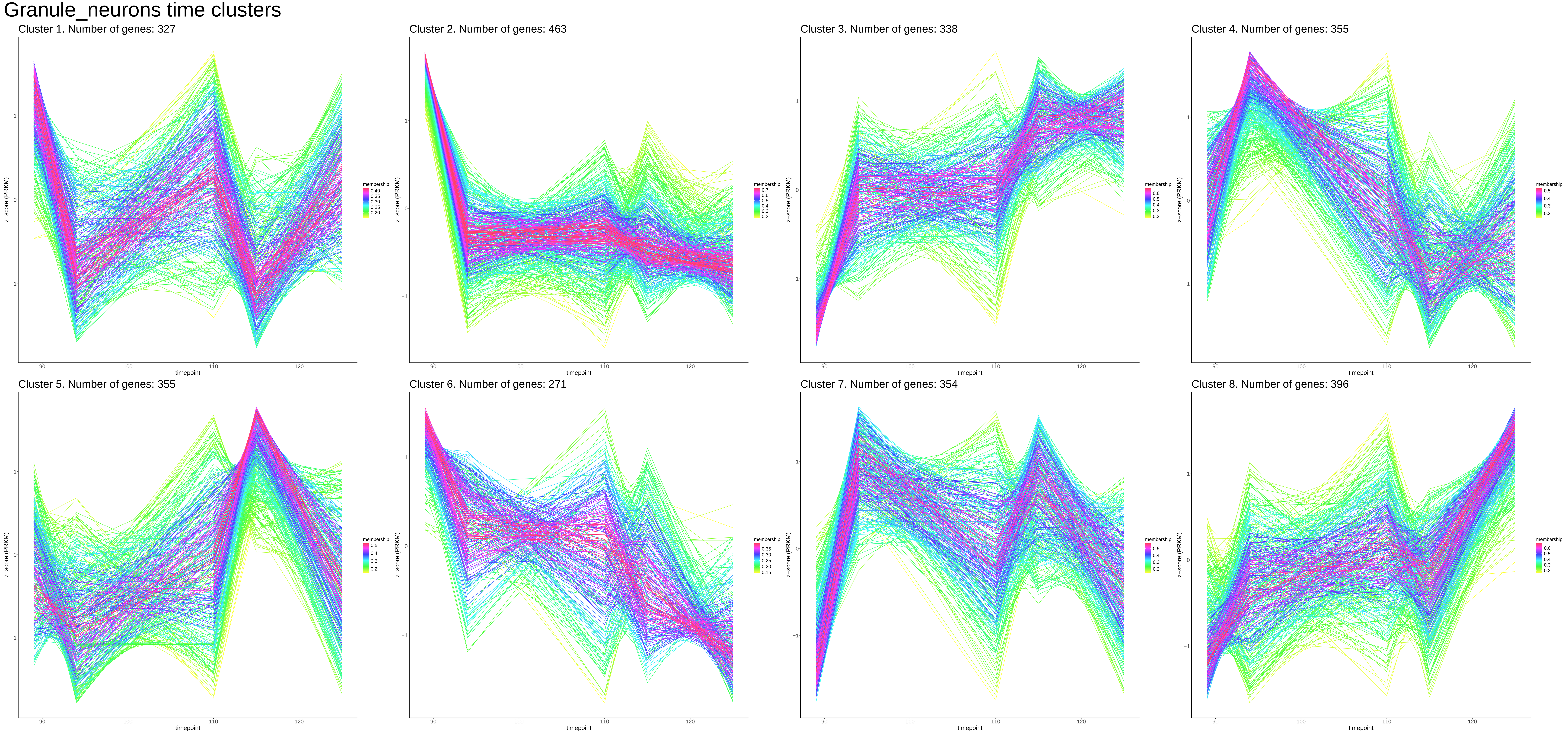
Granule_neurons time clusters
Cluster 1. Number of genes: 327
Cluster 2. Number of genes: 463
Cluster 3. Number of genes: 338
Cluster 4. Number of genes: 355
Cluster 5. Number of genes: 355
Cluster 6. Number of genes: 271
Cluster 7. Number of genes: 354
Cluster 8. Number of genes: 396
z−score (PRKM)
timepoint
90
100
110
120
membership
0.40 0.35 0.30 0.25 0.20
0.7 0.6 0.5 0.4 0.3 0.2
0.6 0.5 0.4 0.3 0.2
0.5 0.4 0.3 0.2
0.5 0.4 0.3 0.2
0.35 0.30 0.25 0.20 0.15
0.5 0.4 0.3 0.2
0.6 0.5 0.4 0.3 0.2

Hematopoietic_stem_cells time clusters

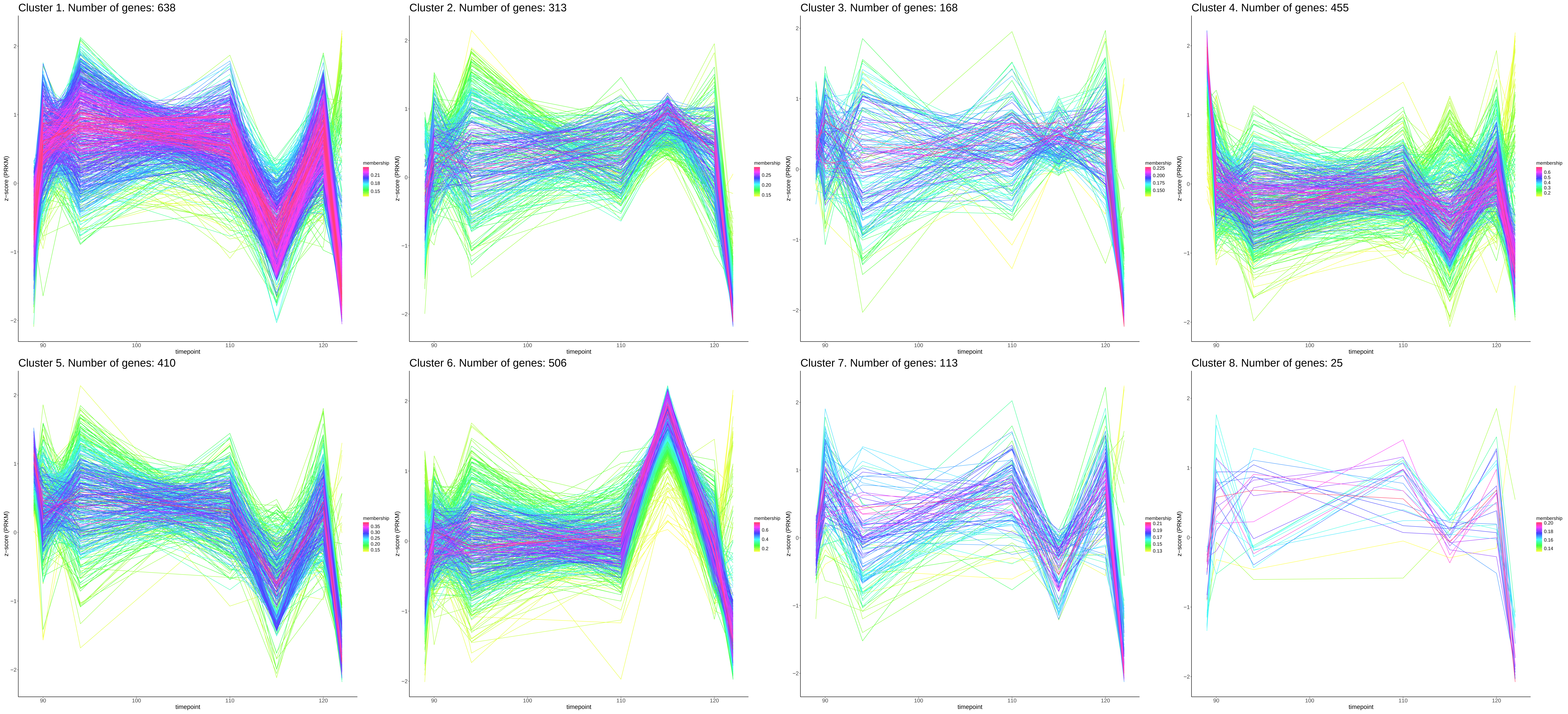

Hepatoblasts time clusters

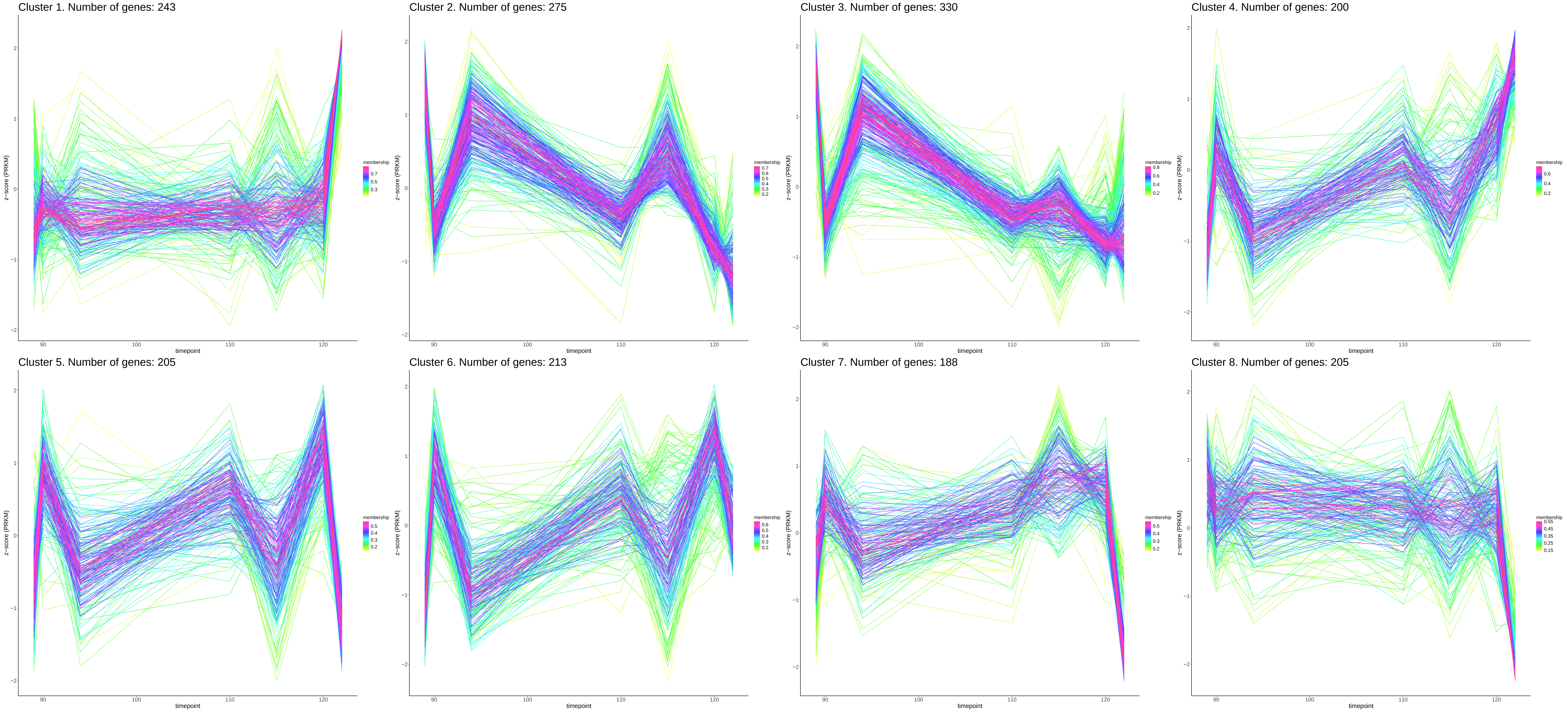

Horizontal_cells time clusters

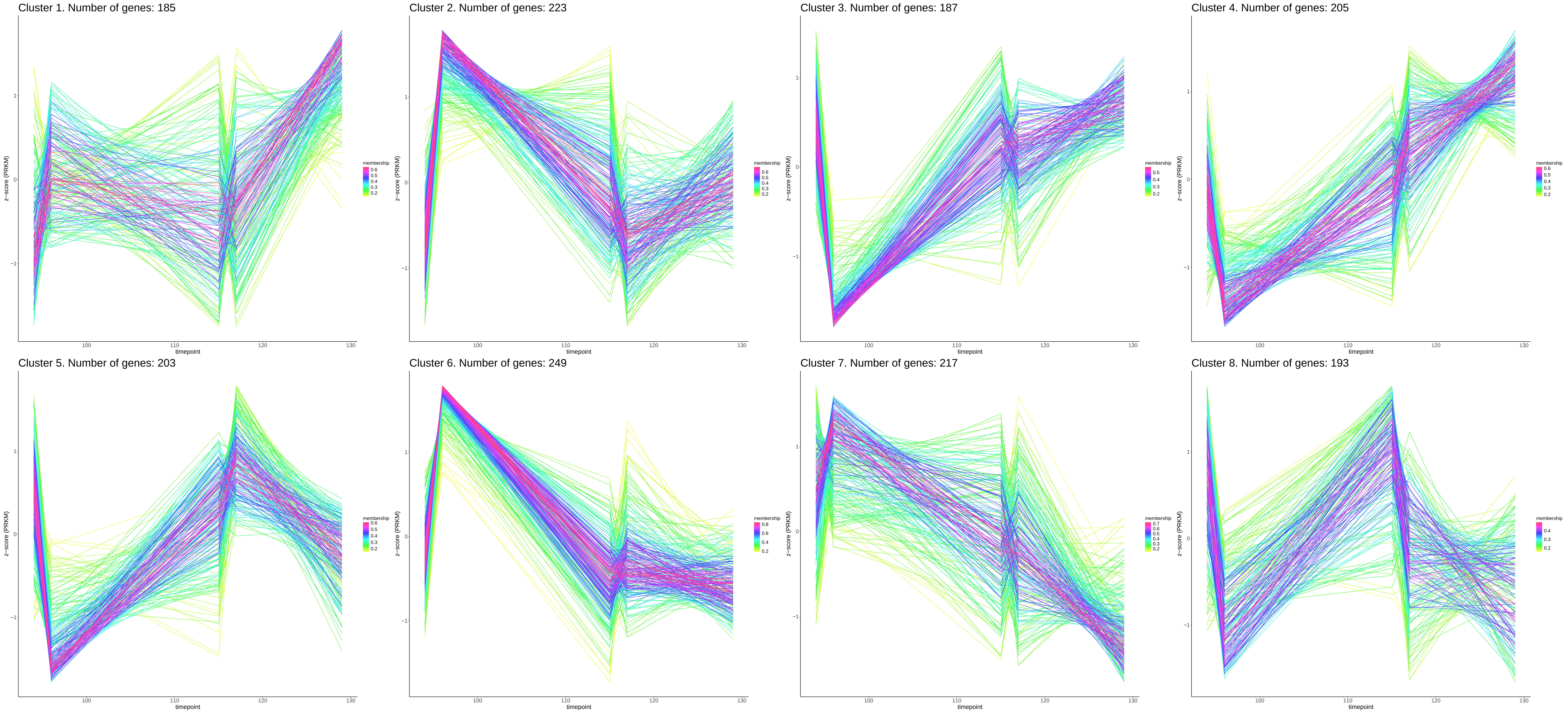

Inhibitory_interneurons time clusters

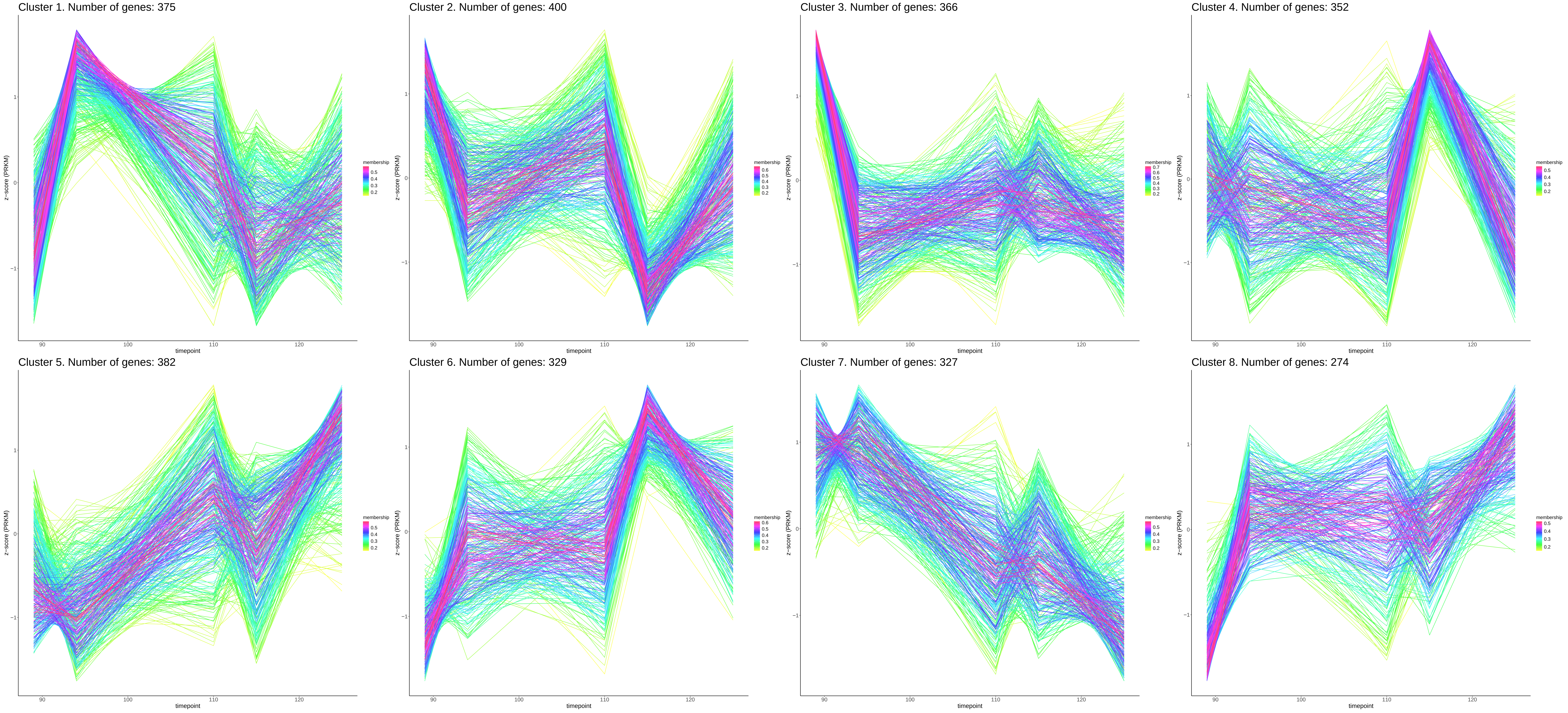

Inhibitory_neurons time clusters

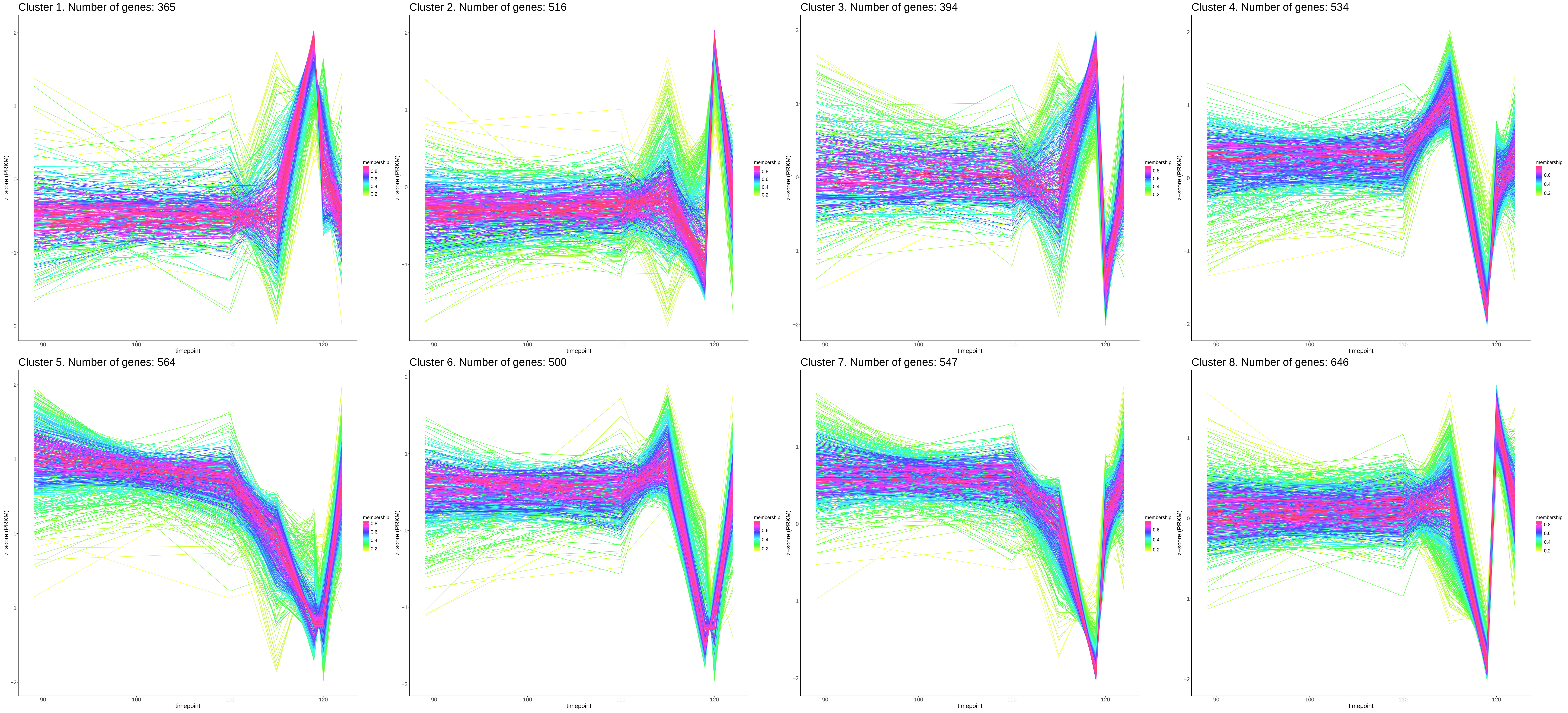

Intestinal_epithelial_cells time clusters

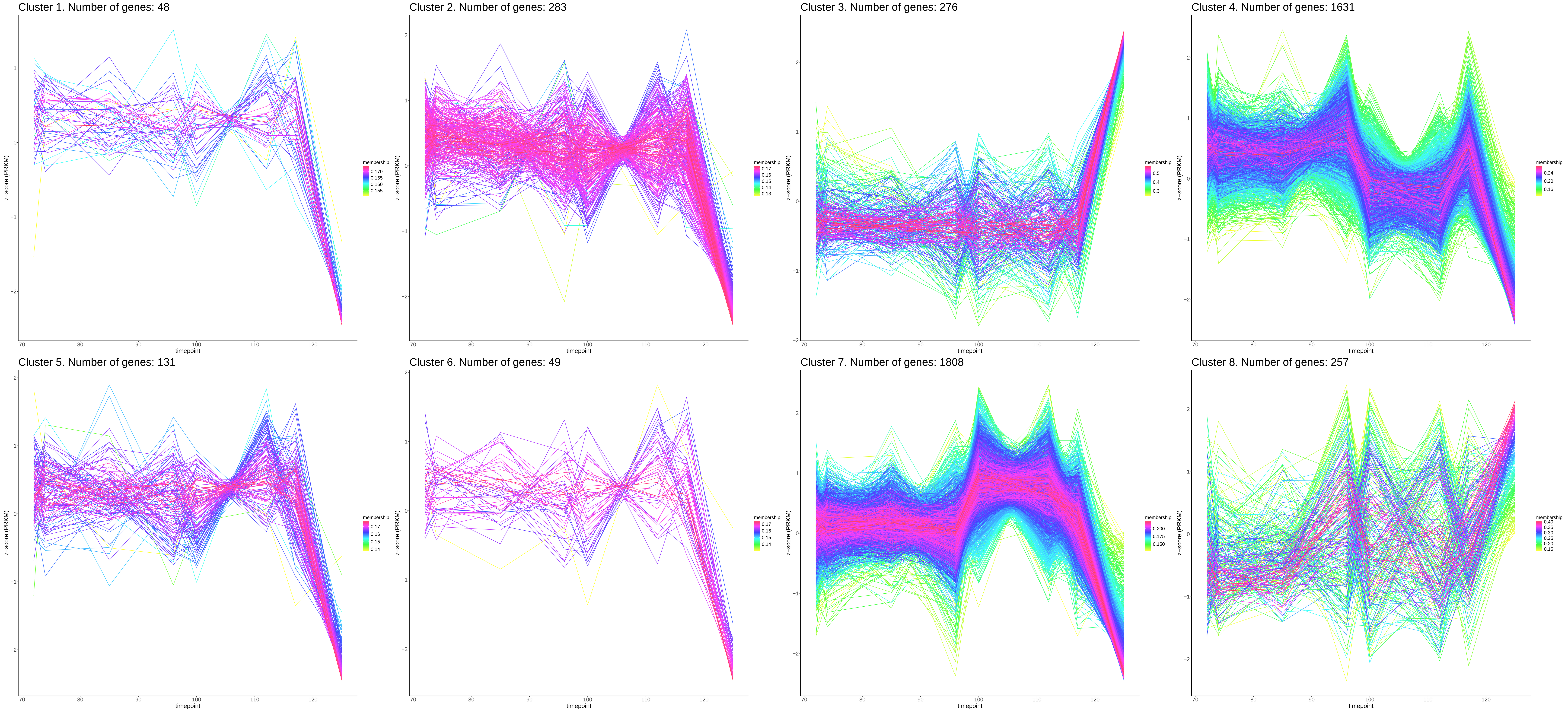

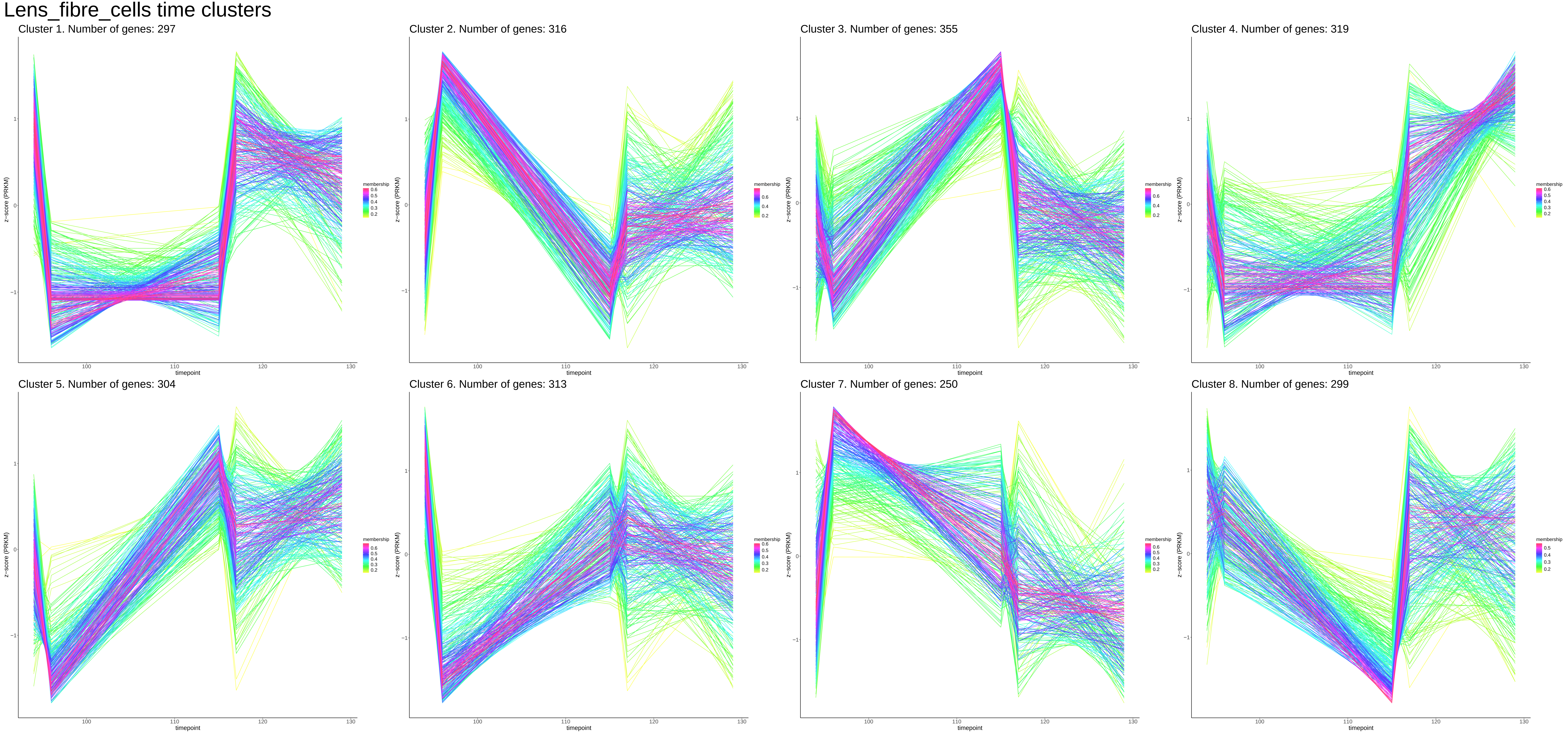
Lens_fibre_cells time clusters
Cluster 1. Number of genes: 297
Cluster 2. Number of genes: 316
Cluster 3. Number of genes: 355
Cluster 4. Number of genes: 319
Cluster 5. Number of genes: 304
Cluster 6. Number of genes: 313
Cluster 7. Number of genes: 250
Cluster 8. Number of genes: 299
z−score (PRKM)
timepoint
membership

Mesangial_cells time clusters

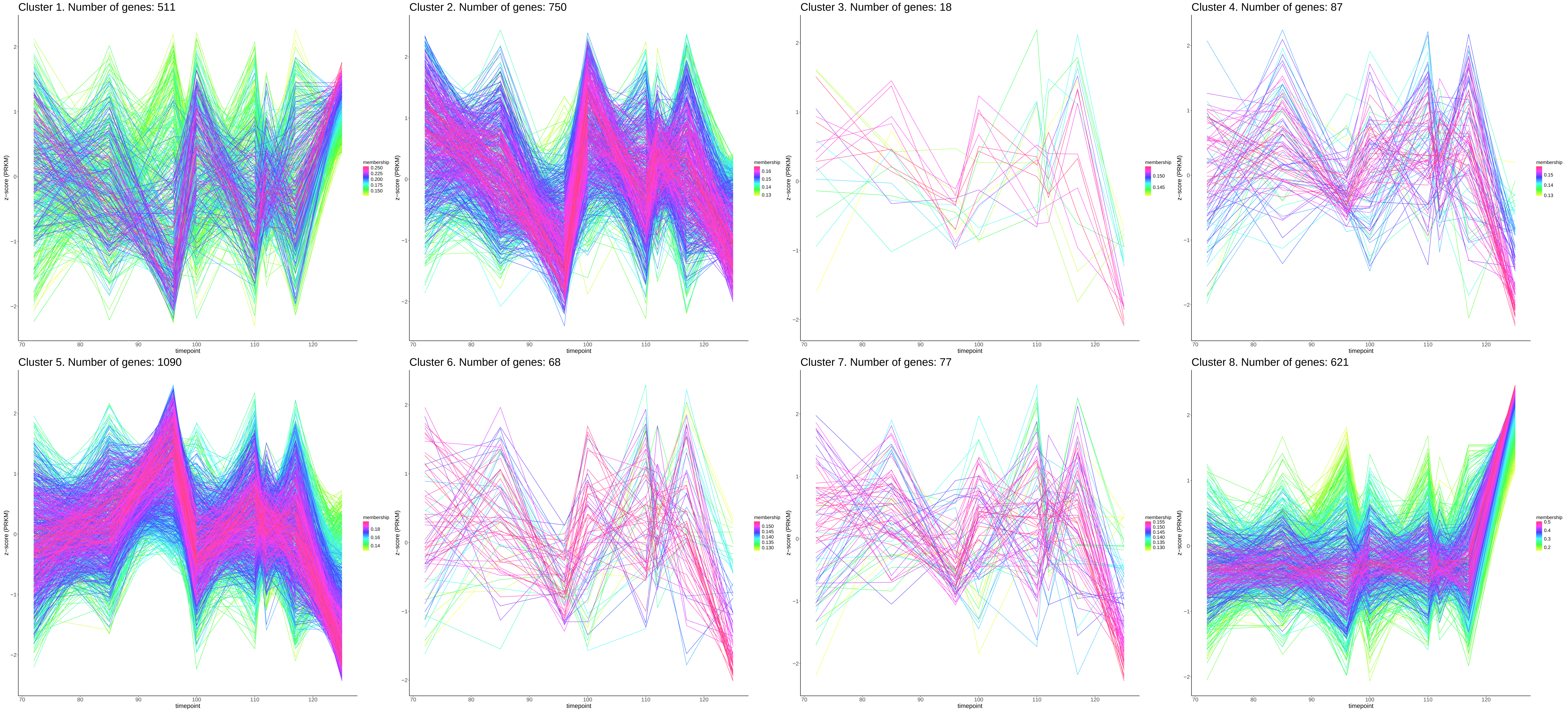

Metanephric_cells time clusters

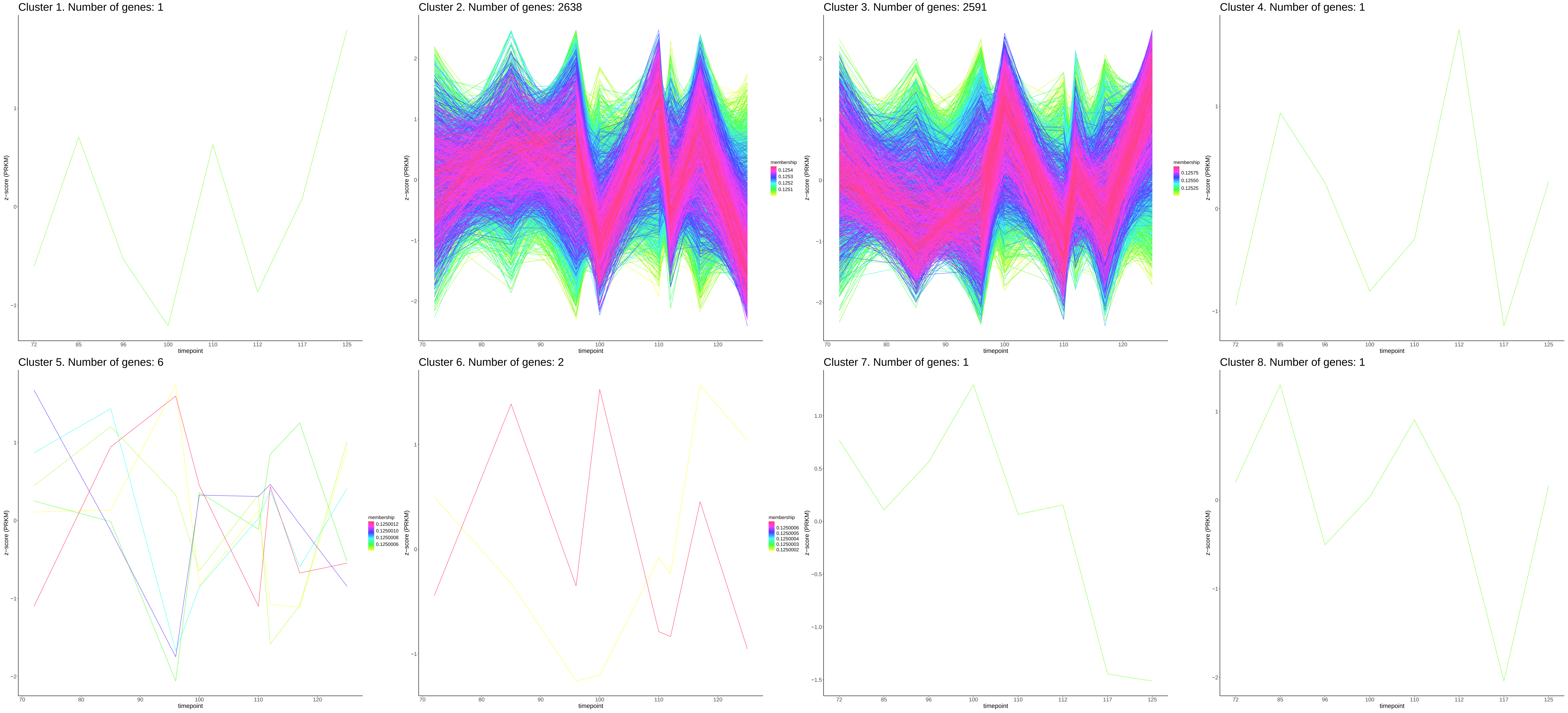

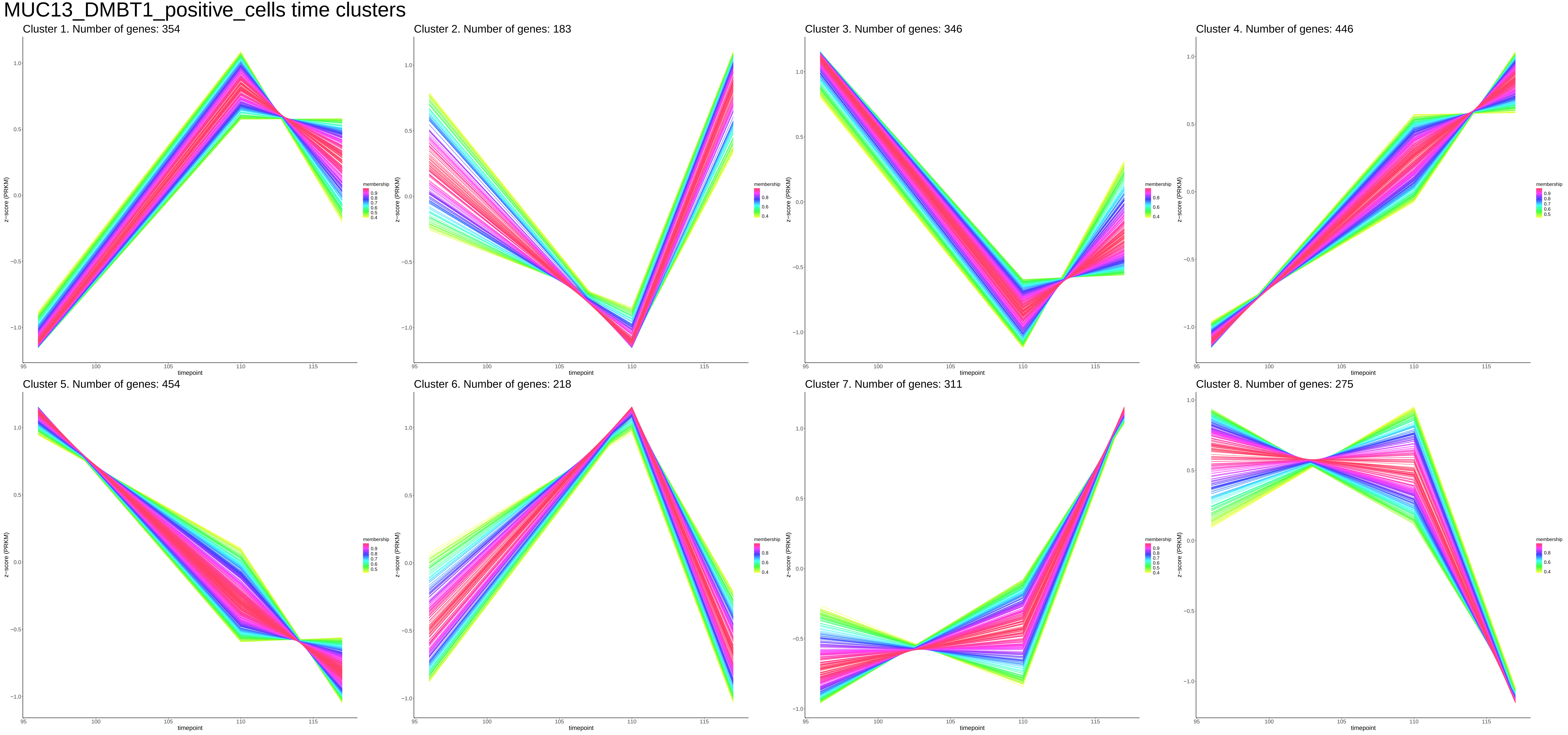
MUC13_DMBT1_positive_cells time clusters
Cluster 1. Number of genes: 354
Cluster 2. Number of genes: 183
Cluster 3. Number of genes: 346
Cluster 4. Number of genes: 446
Cluster 5. Number of genes: 454
Cluster 6. Number of genes: 218
Cluster 7. Number of genes: 311
Cluster 8. Number of genes: 275
z−score (PRKM)
timepoint
membership
95
100
105
110
115
−1.0
−0.5
0.0
0.5
1.0
0.4
0.5
0.6
0.7
0.8
0.9

Myeloid_cells time clusters

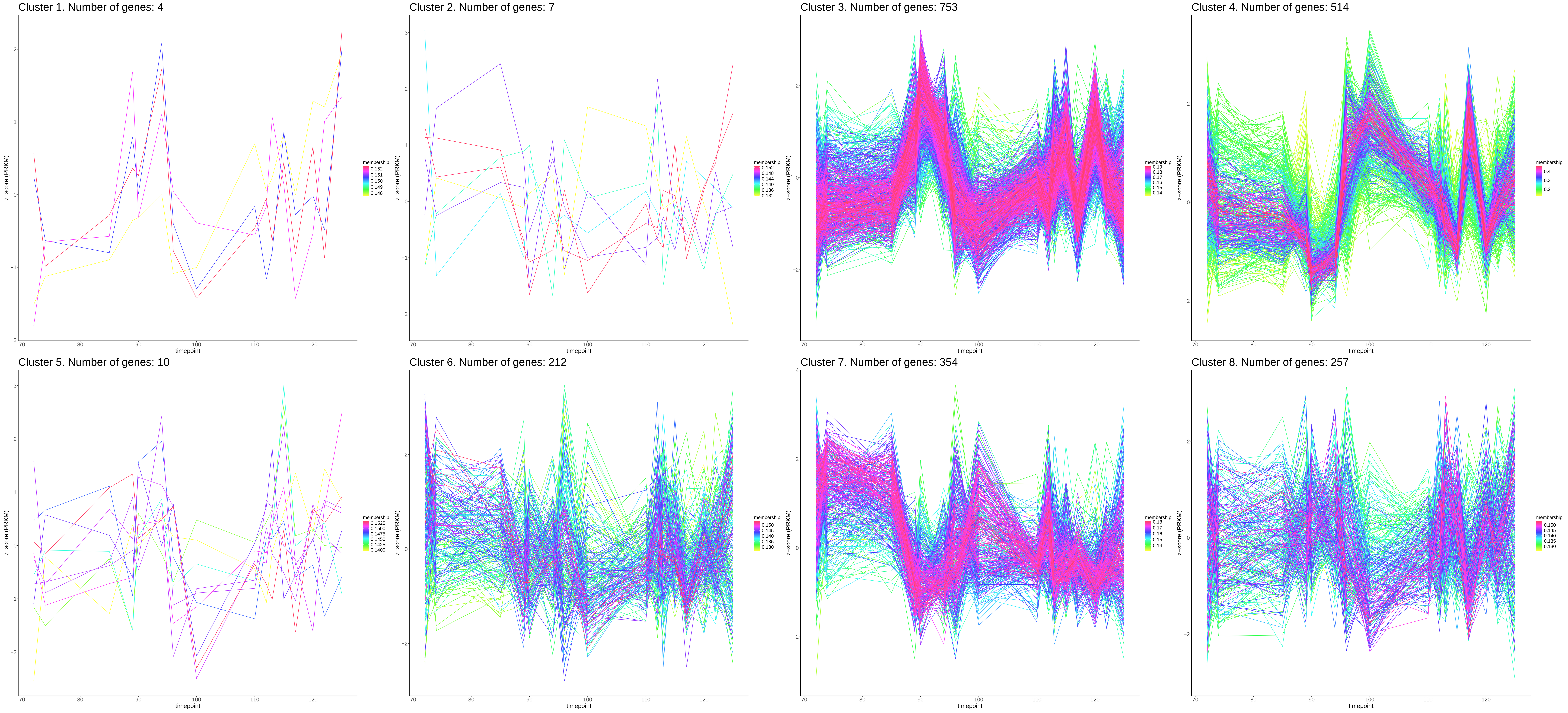

Oligodendrocytes time clusters

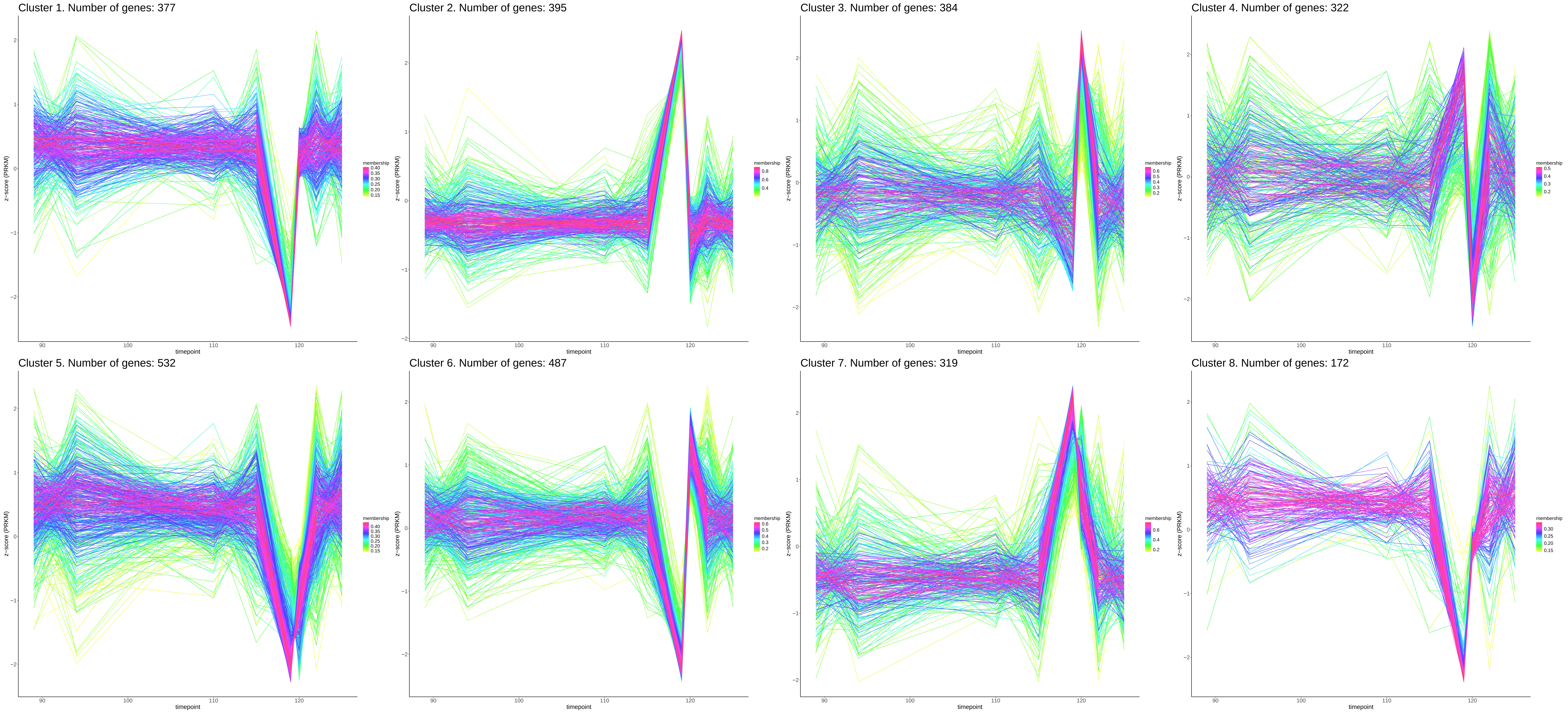

PAEP_MECOM_positive_cells time clusters

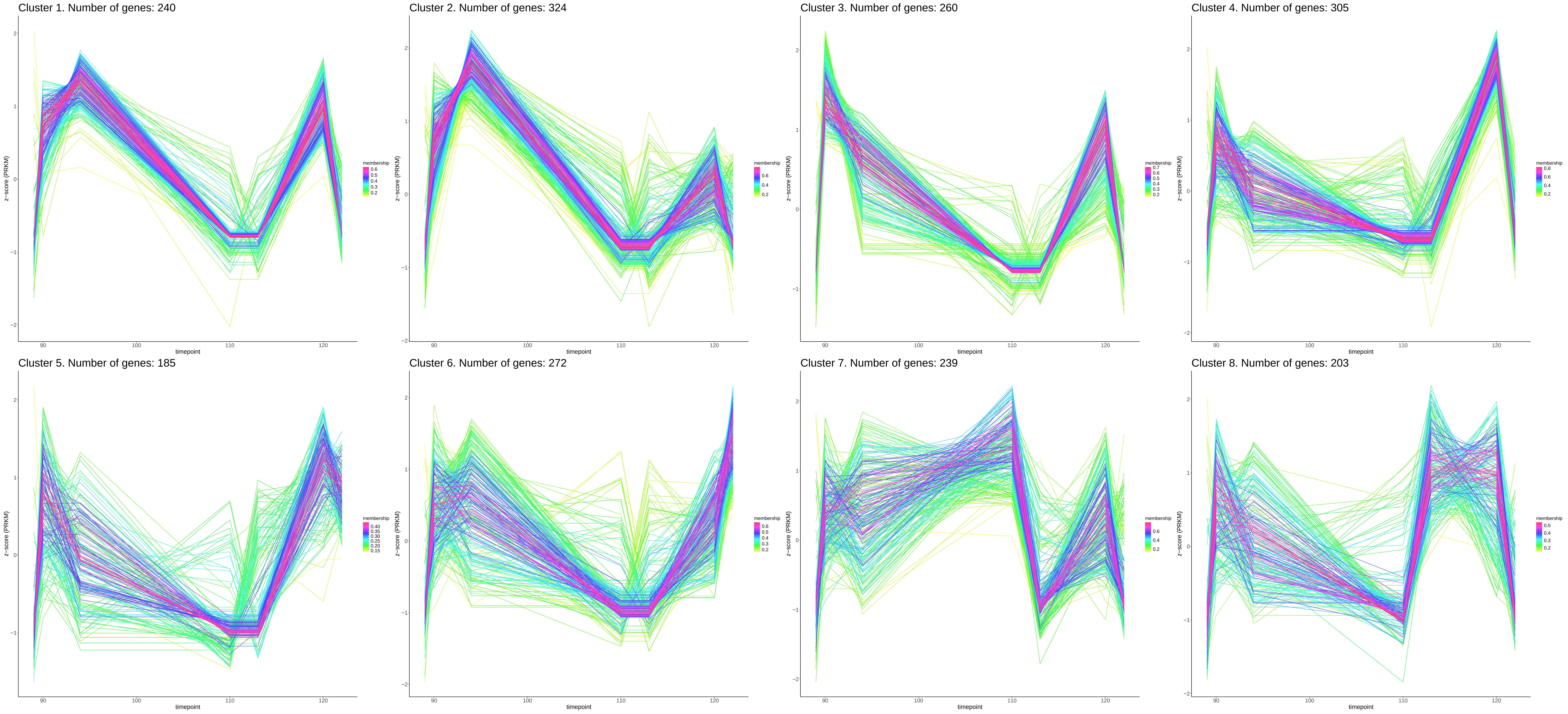

Parietal_and_chief_cells time clusters

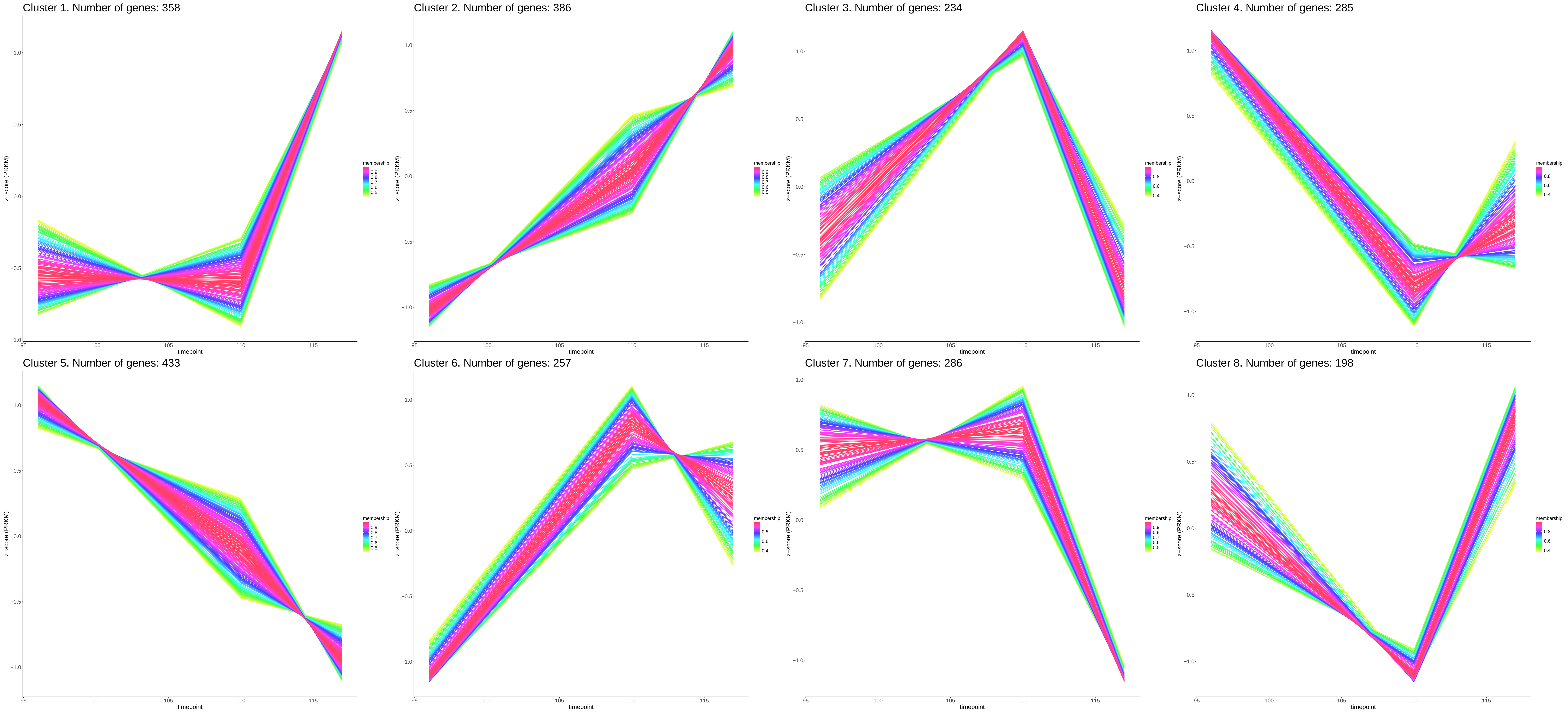

PDE11A_FAM19A2_positive_cells time clusters

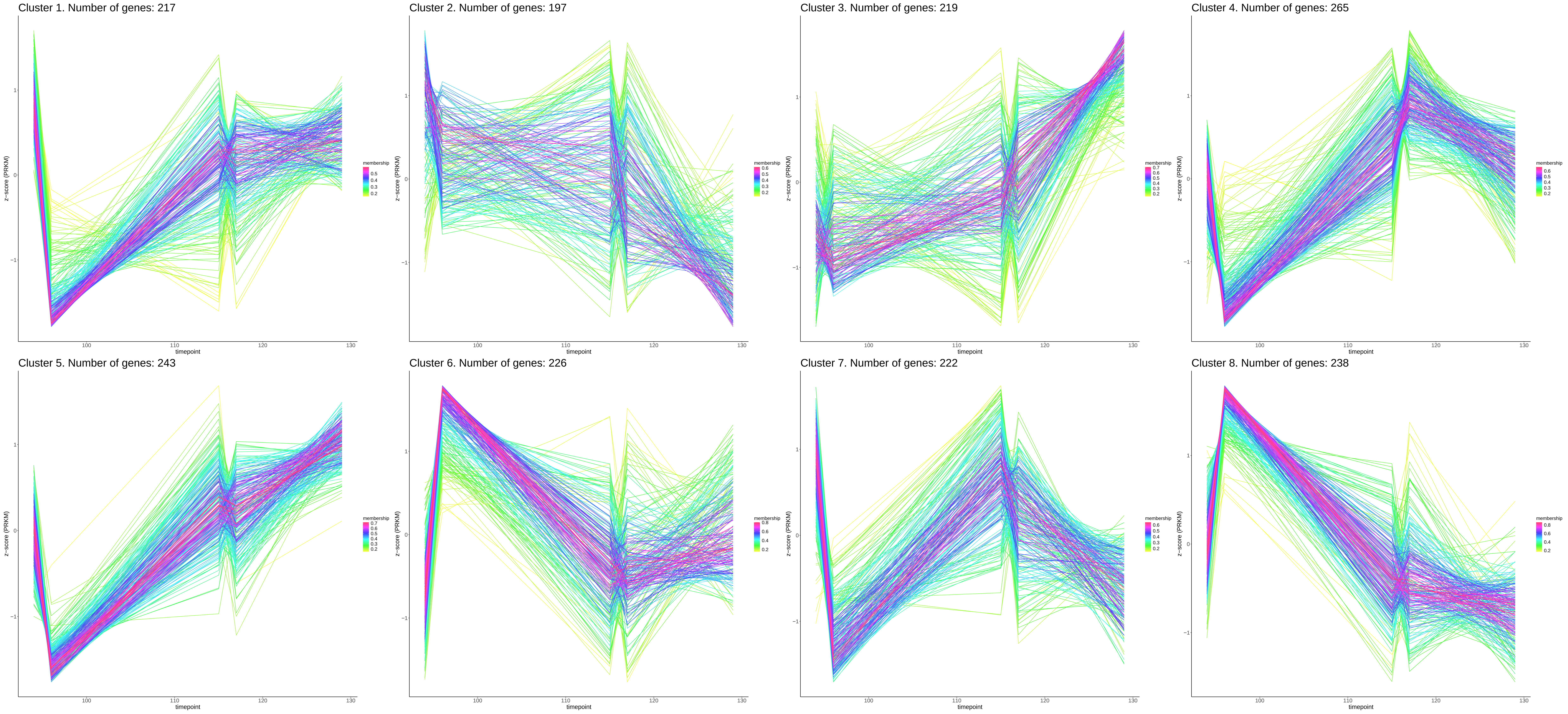

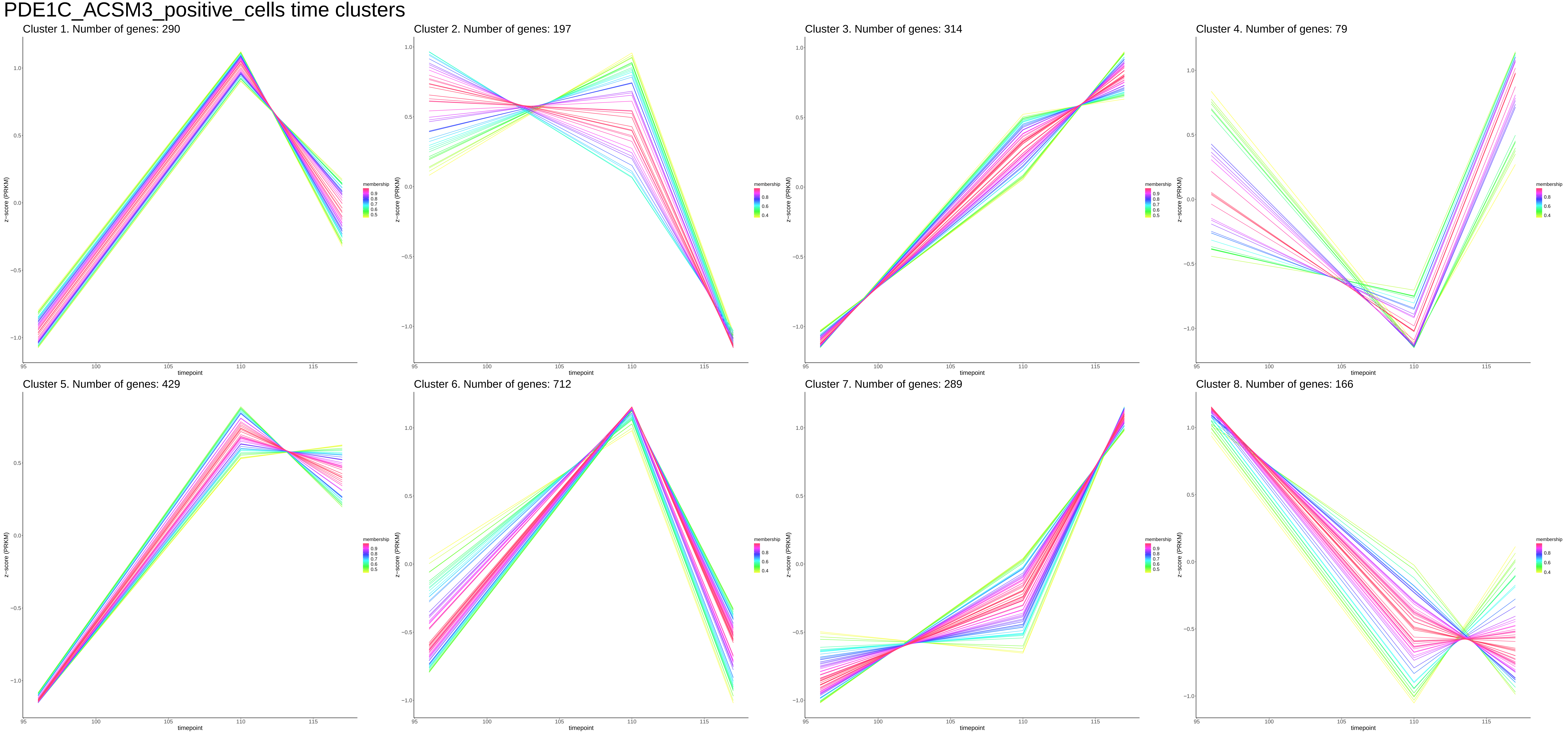
PDE1C_ACSM3_positive_cells time clusters
Cluster 1. Number of genes: 290
Cluster 2. Number of genes: 197
Cluster 3. Number of genes: 314
Cluster 4. Number of genes: 79
Cluster 5. Number of genes: 429
Cluster 6. Number of genes: 712
Cluster 7. Number of genes: 289
Cluster 8. Number of genes: 166
z−score (PRKM)
timepoint
membership

Photoreceptor_cells time clusters

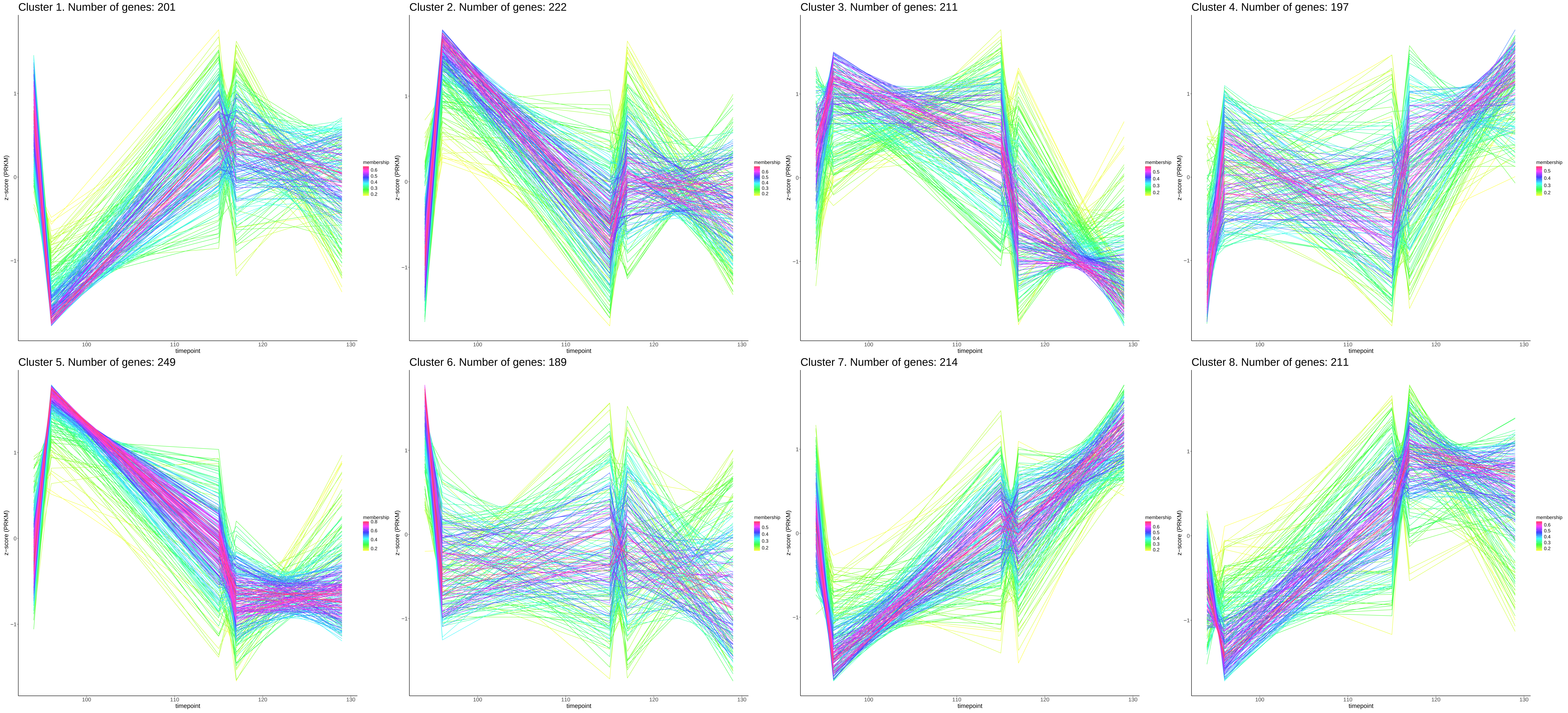

Purkinje_neurons time clusters

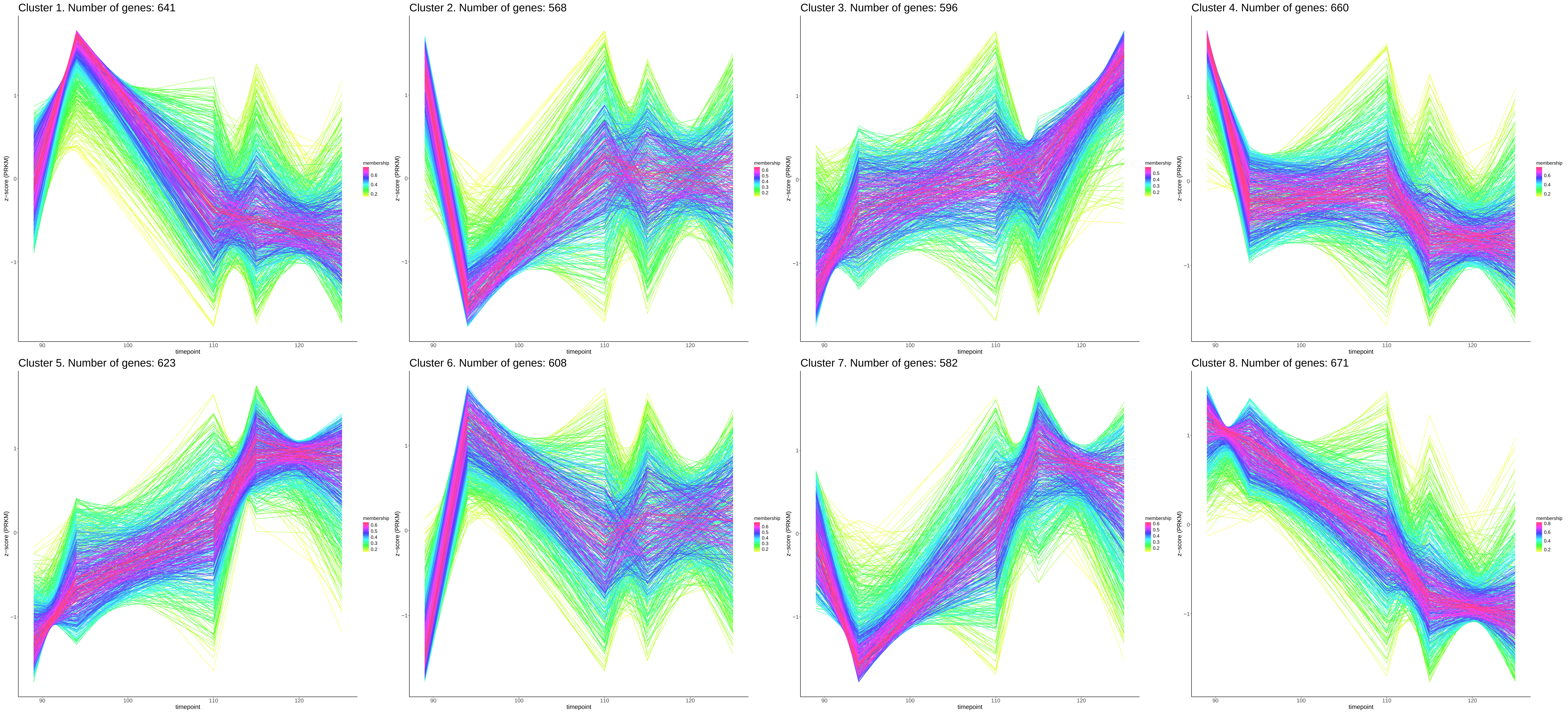

Retinal_pigment_cells time clusters

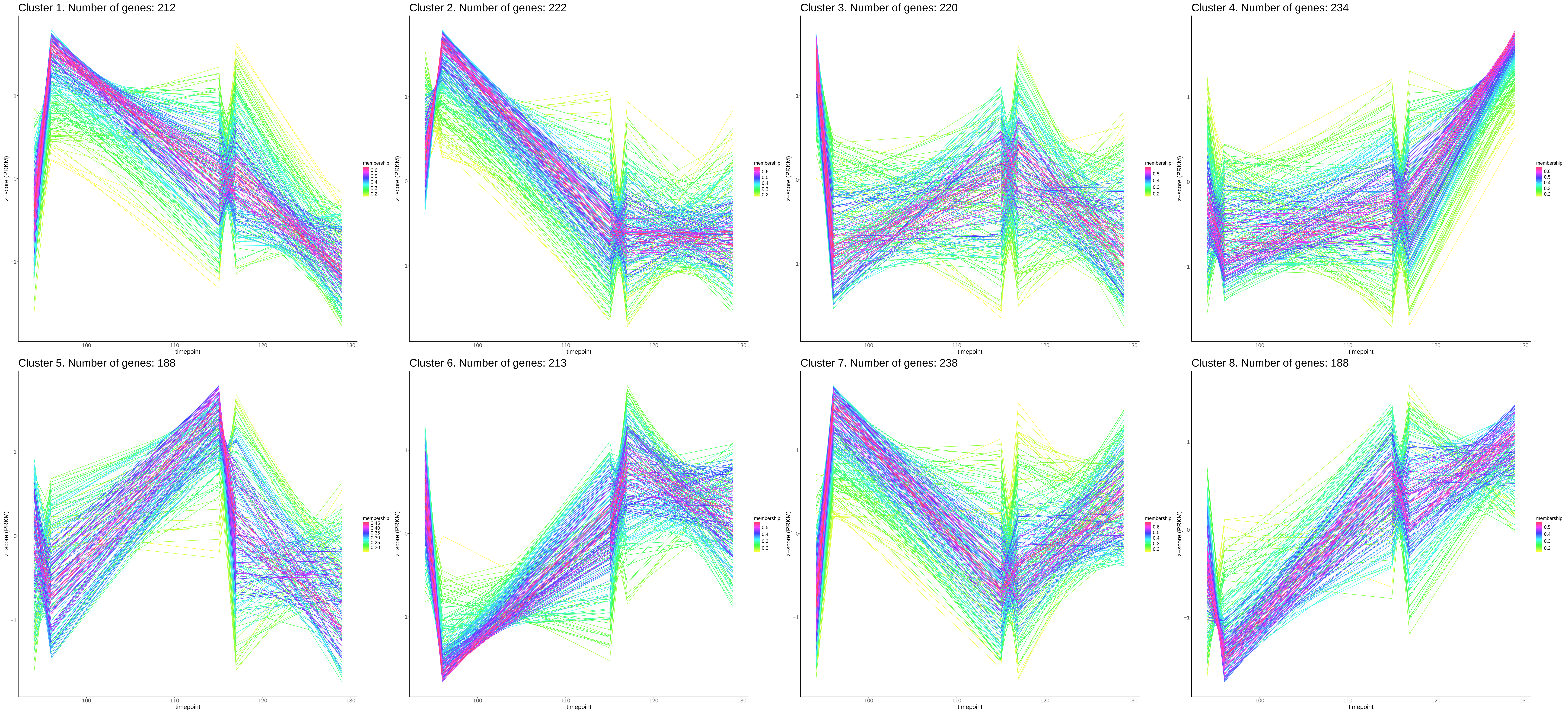

Retinal_progenitors_and_Muller_glia time clusters

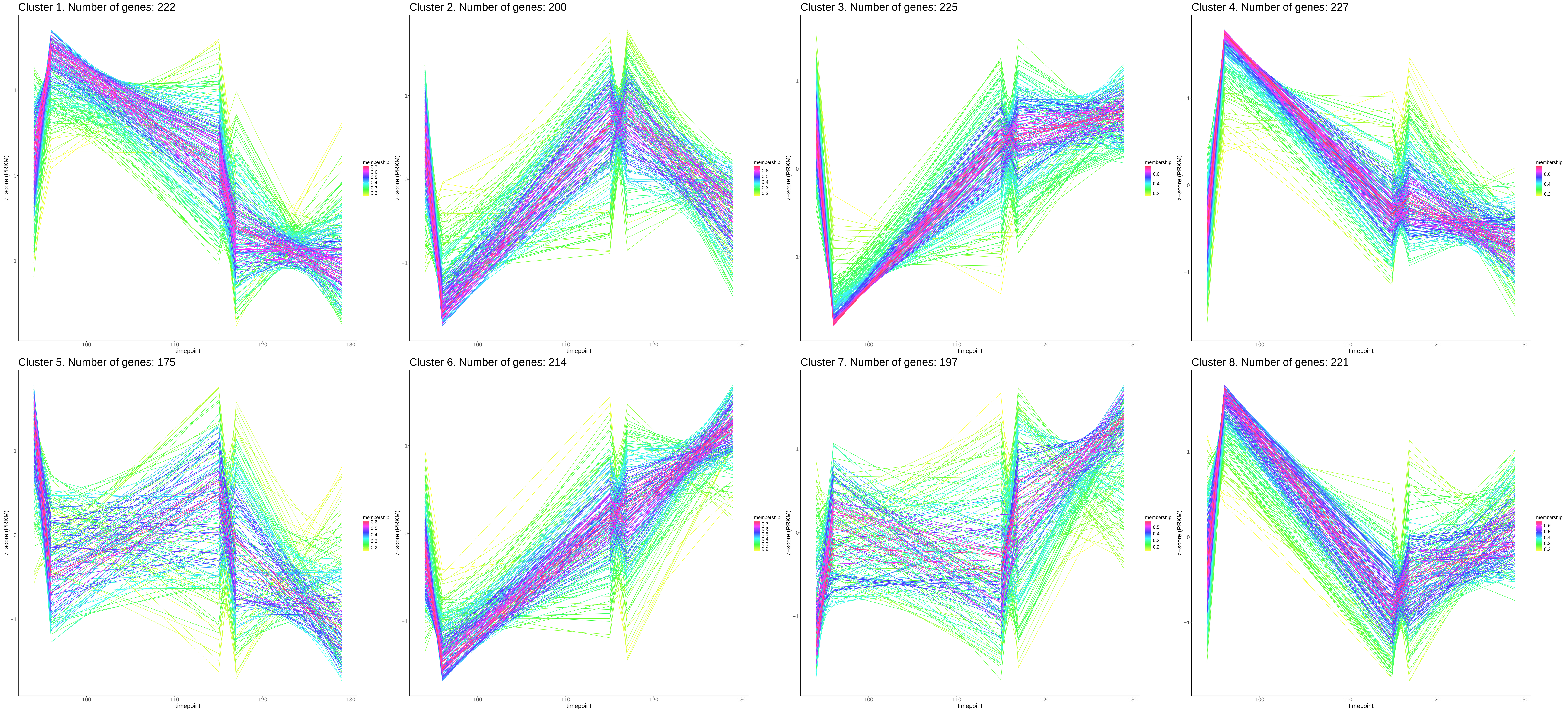

SATB2_LRRC7_positive_cells time clusters

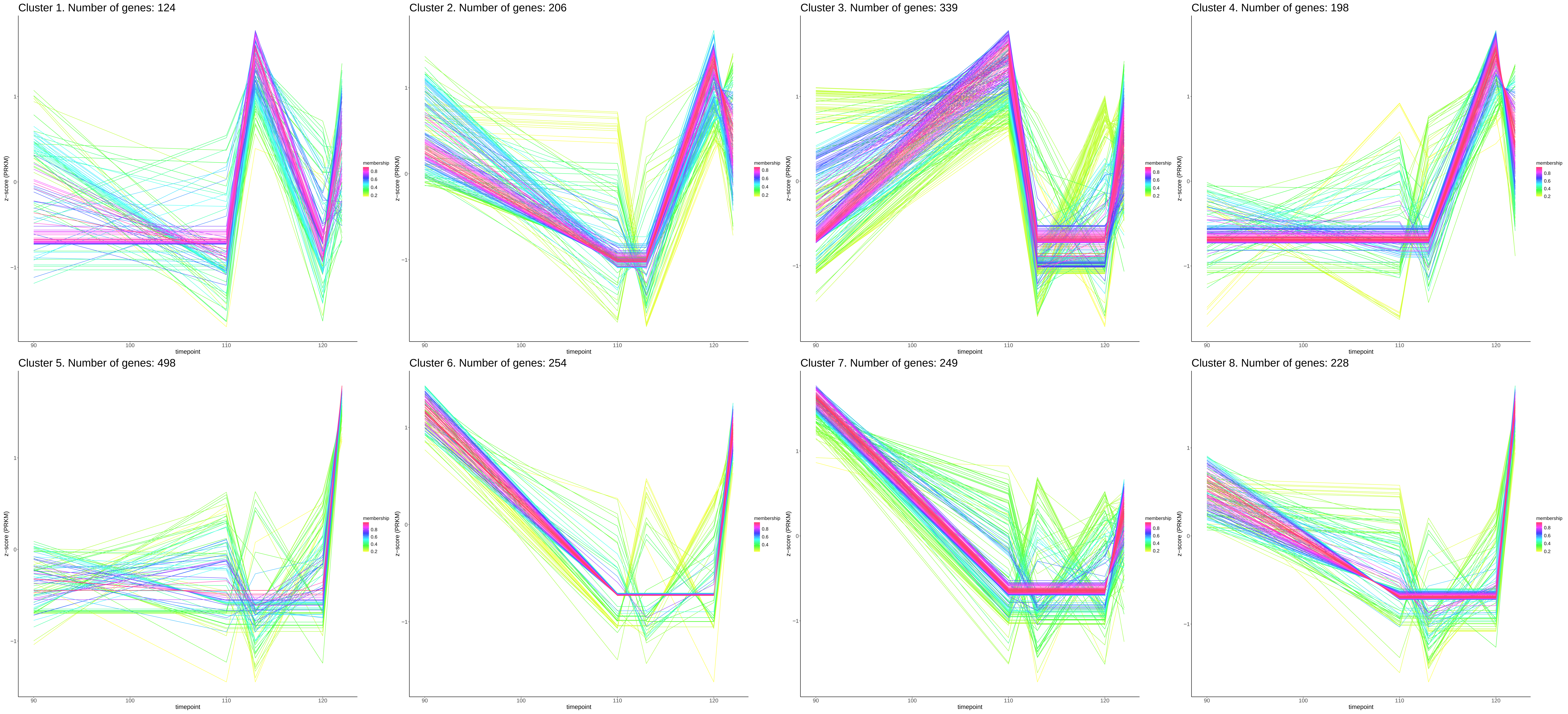

Satellite_cells time clusters

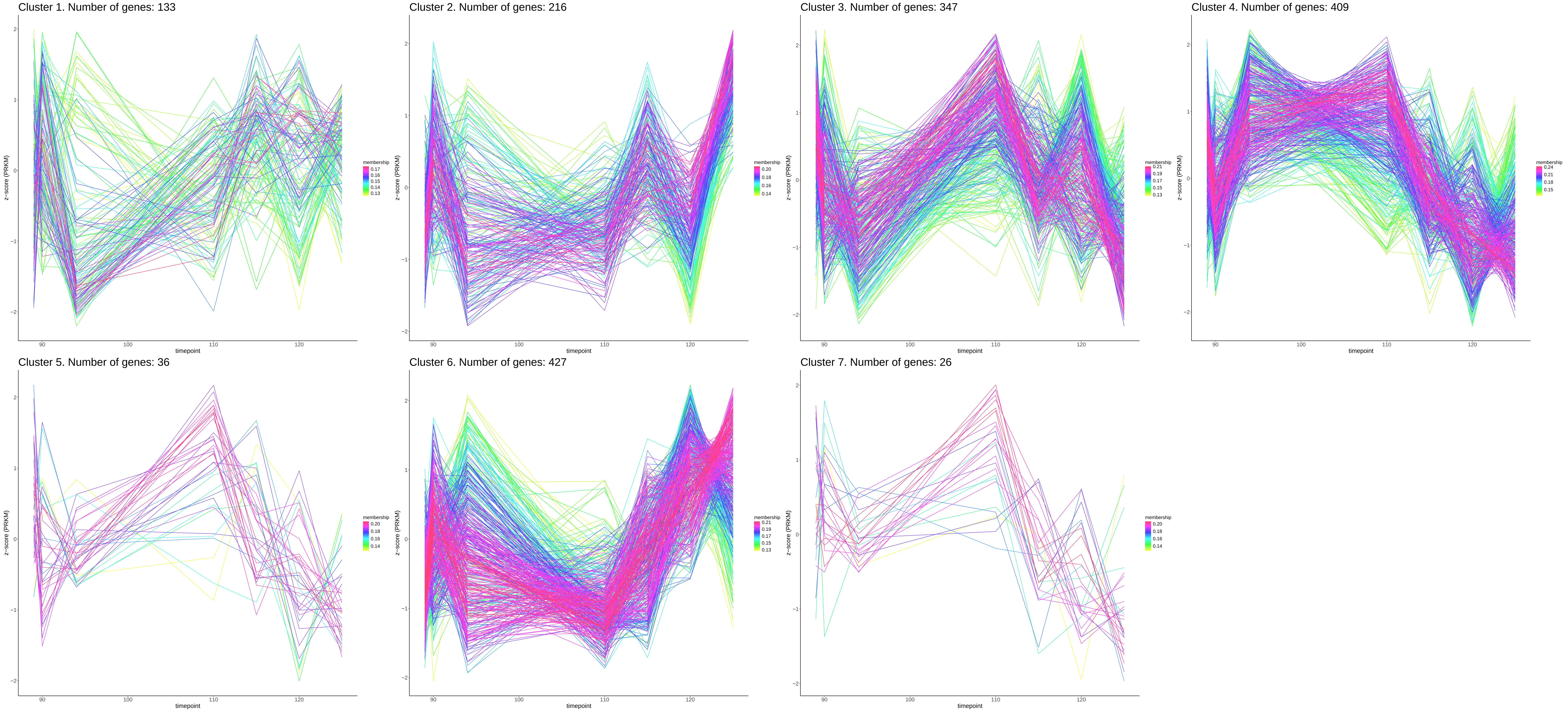

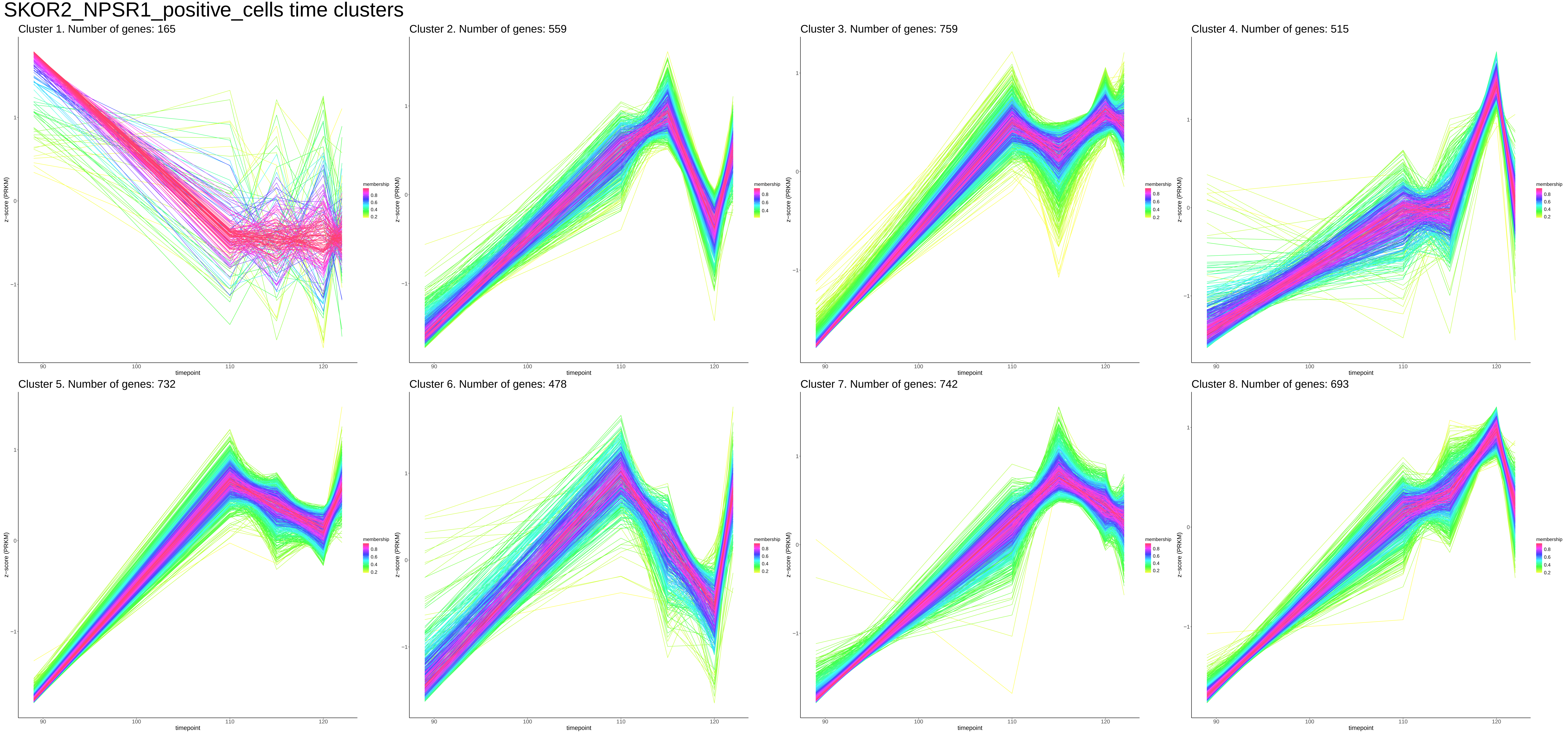
SKOR2_NPSR1_positive_cells time clusters
Cluster 1. Number of genes: 165
Cluster 2. Number of genes: 559
Cluster 3. Number of genes: 759
Cluster 4. Number of genes: 515
Cluster 5. Number of genes: 732
Cluster 6. Number of genes: 478
Cluster 7. Number of genes: 742
Cluster 8. Number of genes: 693
z−score (PRKM)
timepoint
membership

SLC24A4_PEX5L_positive_cells time clusters

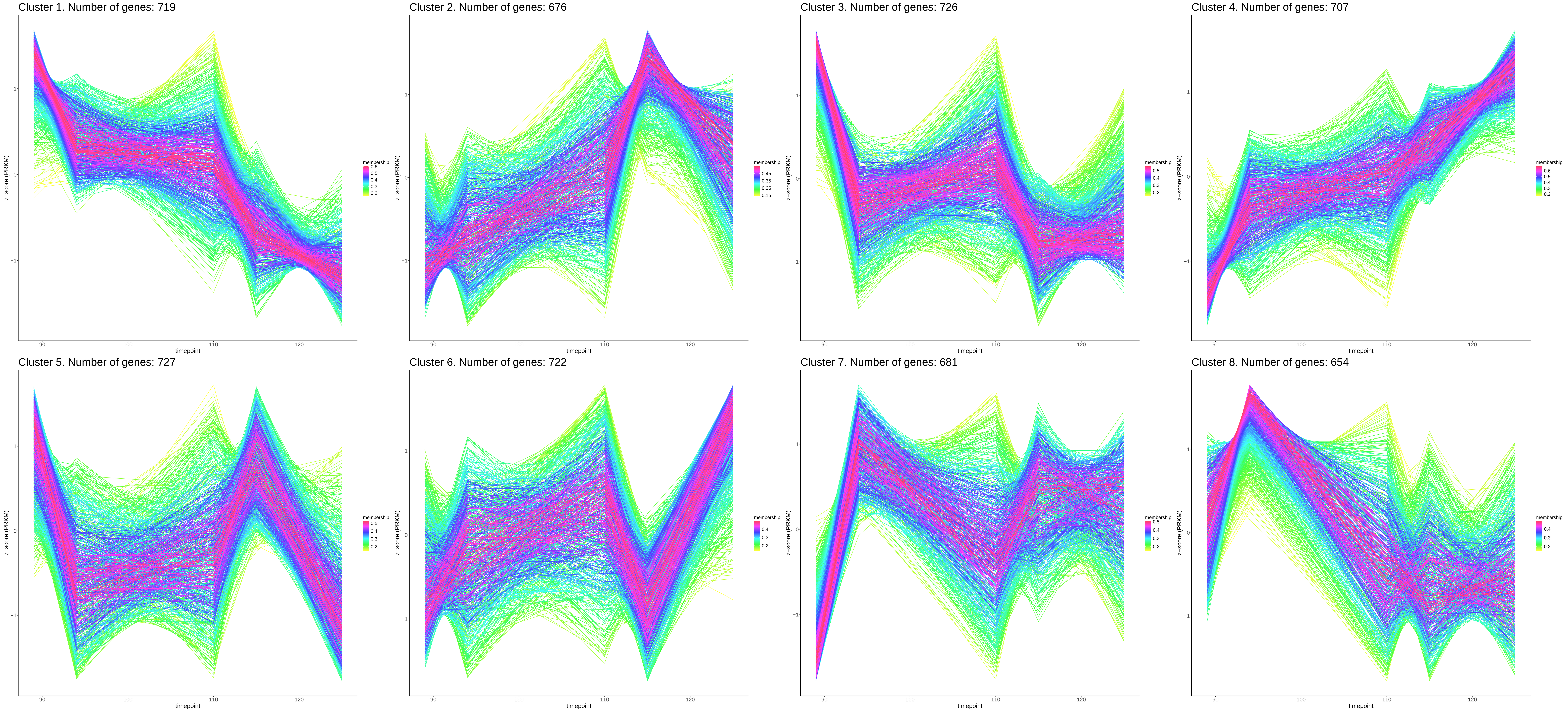

SLC26A4_PAEP_positive_cells time clusters

Cluster 1. Number of genes: 887

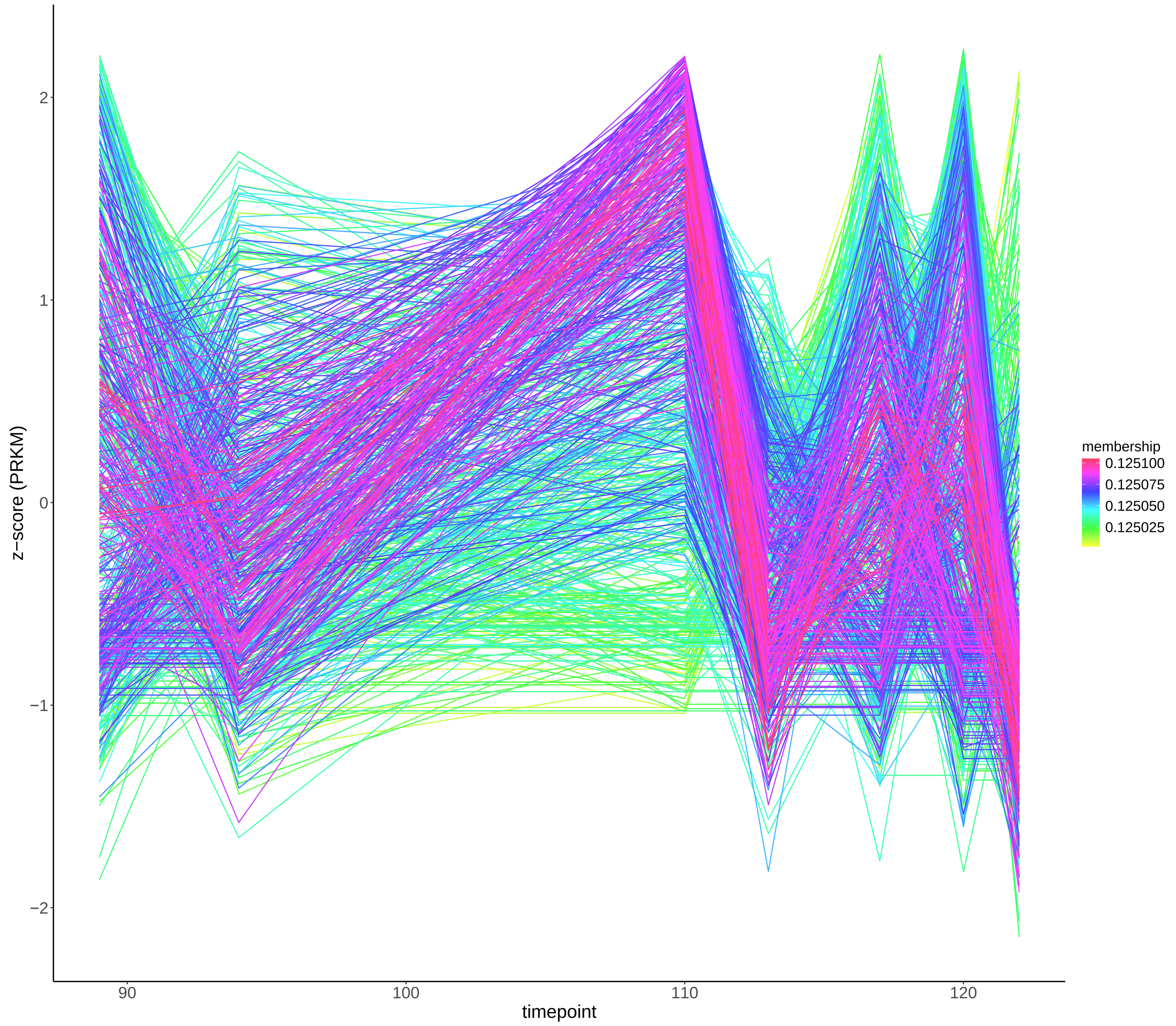

Cluster 2. Number of genes: 567

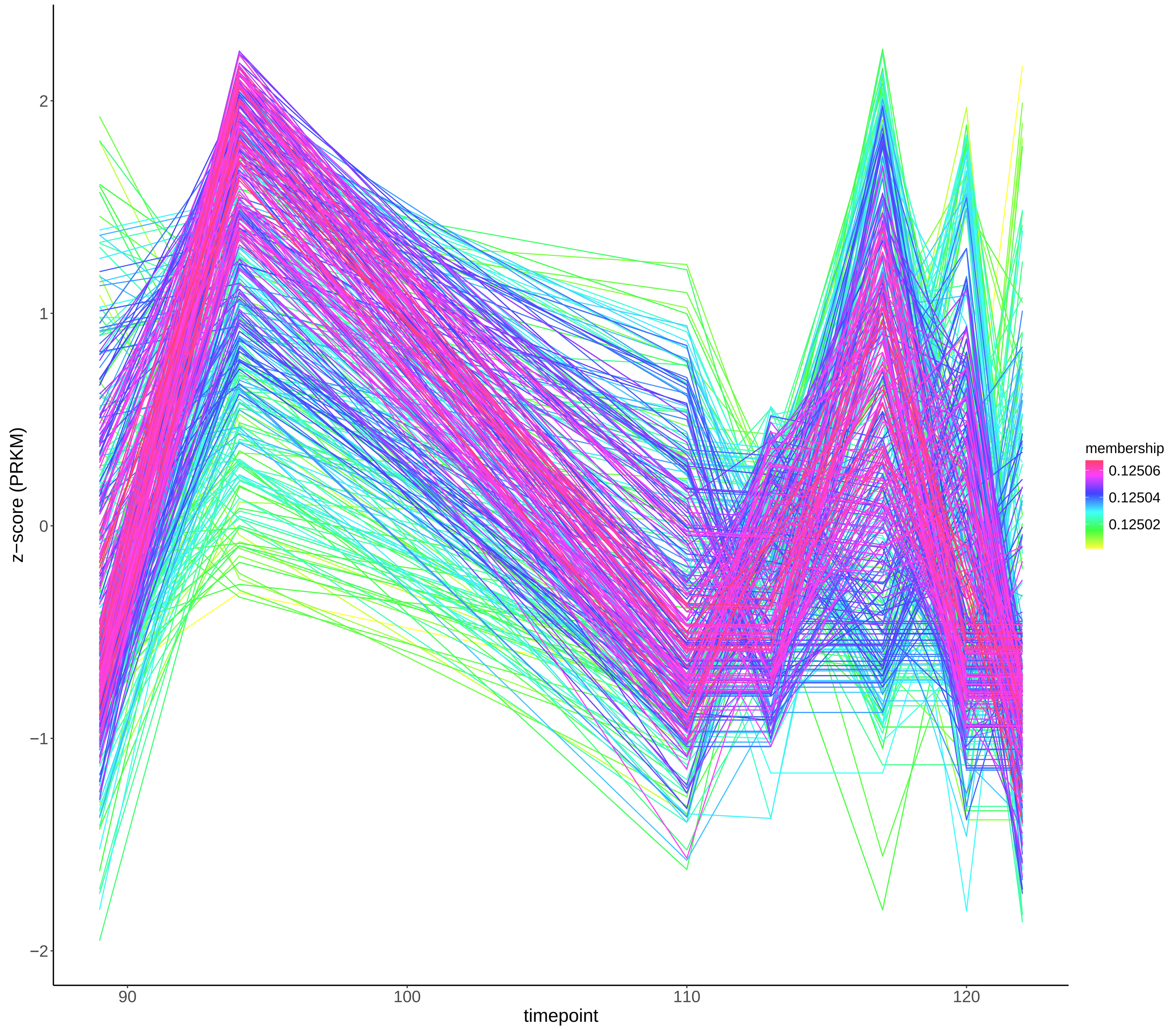

Cluster 3. Number of genes: 1063

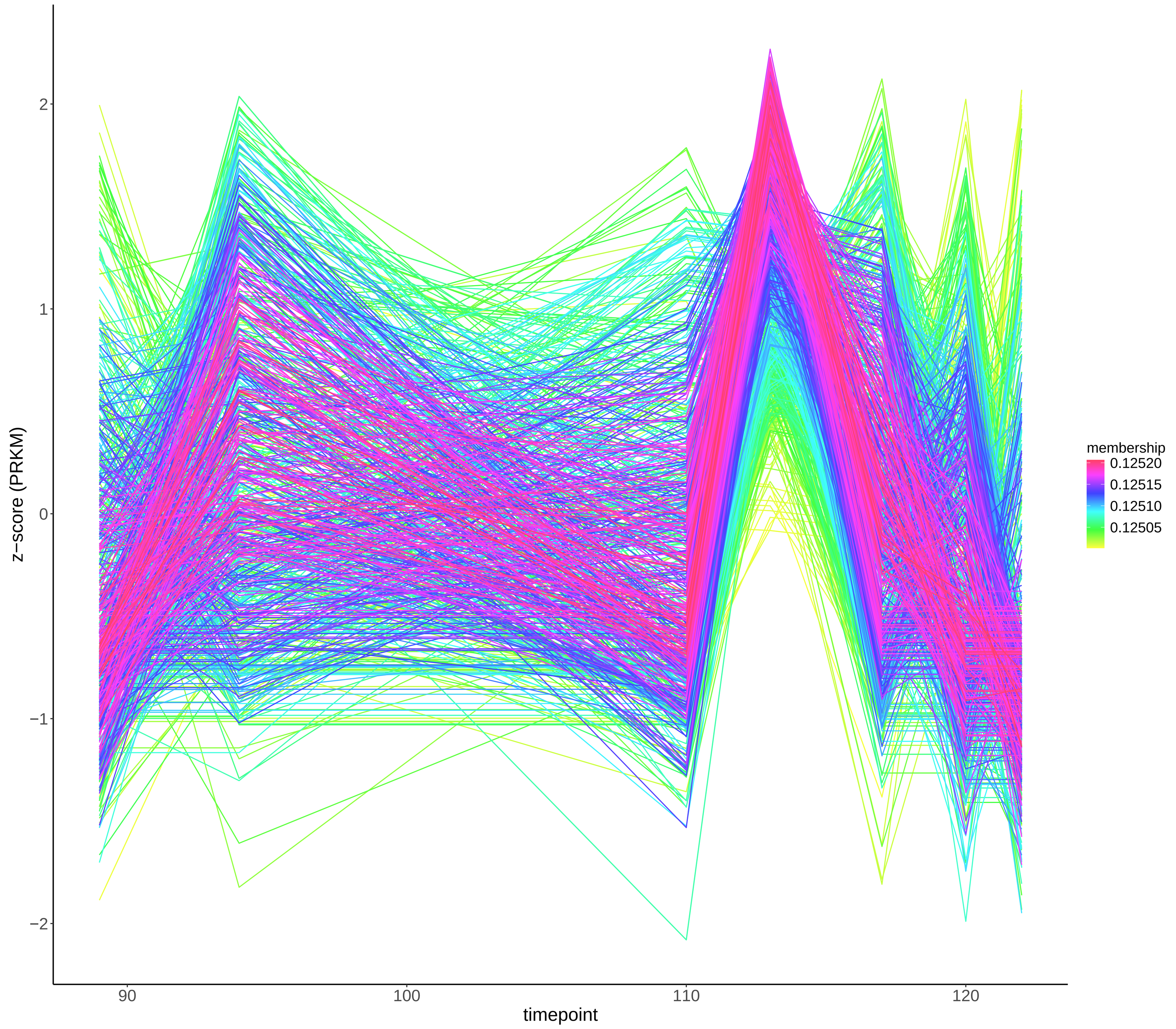

Cluster 4. Number of genes: 17

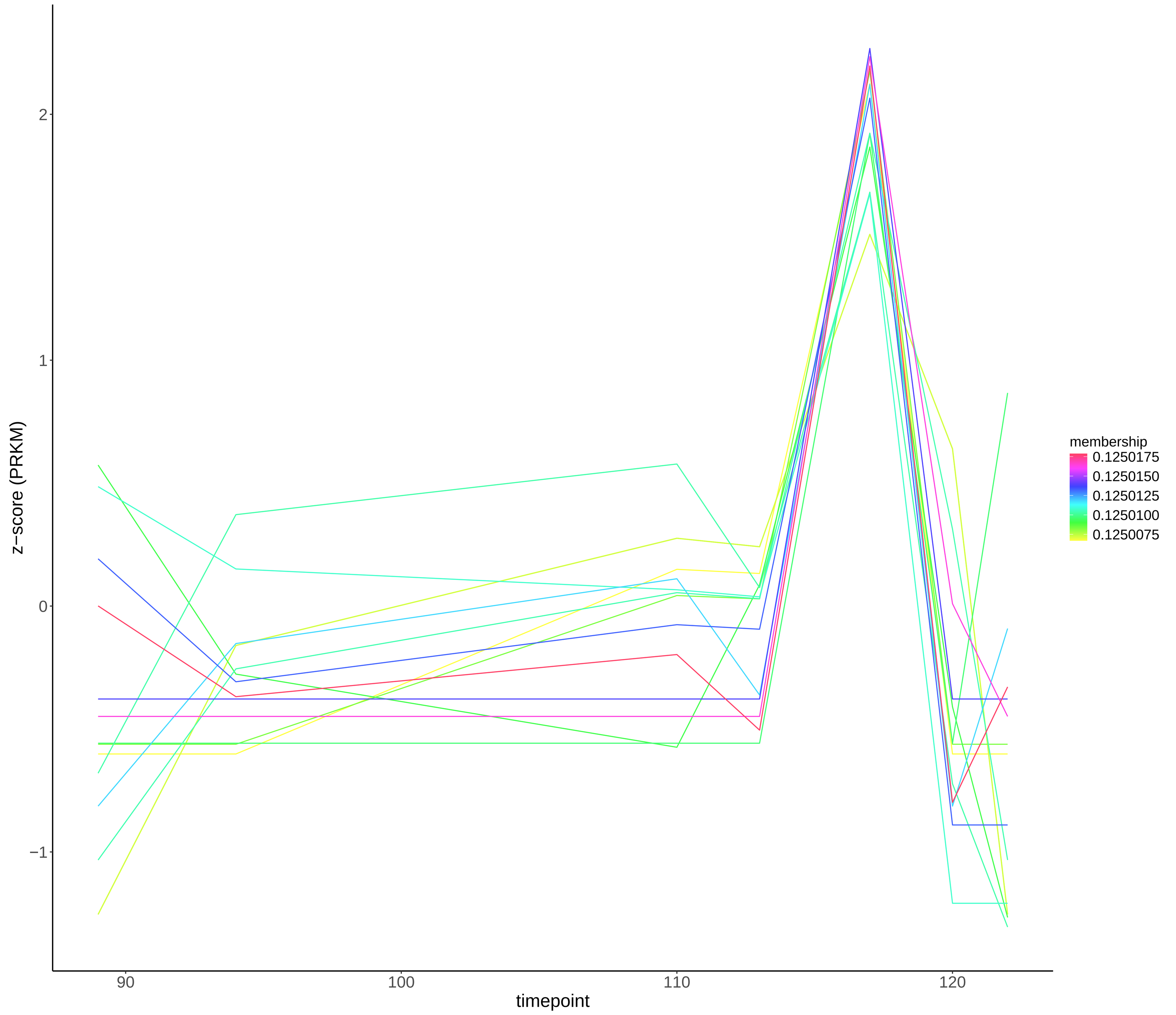

Cluster 5. Number of genes: 77

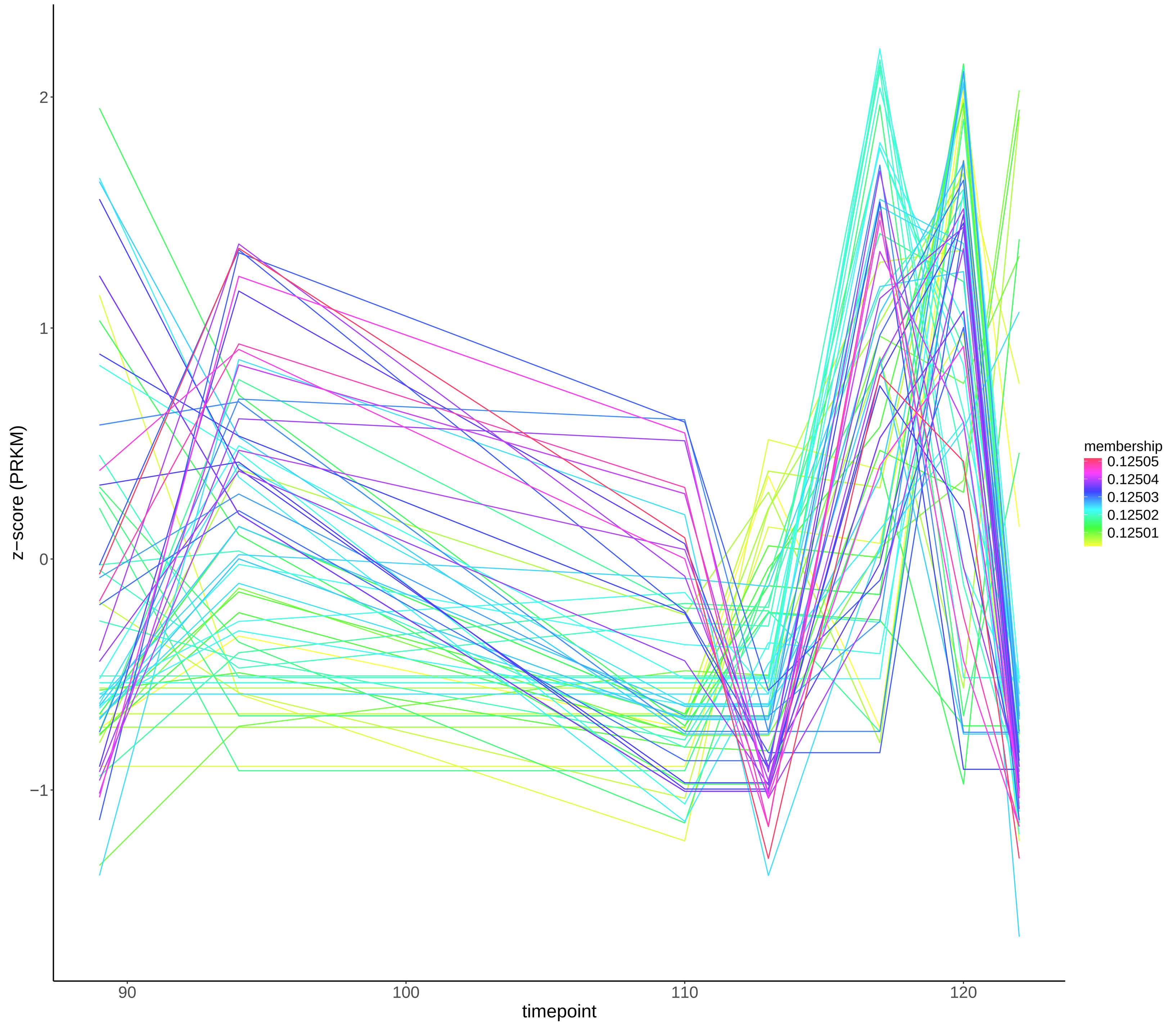

Cluster 6. Number of genes: 13

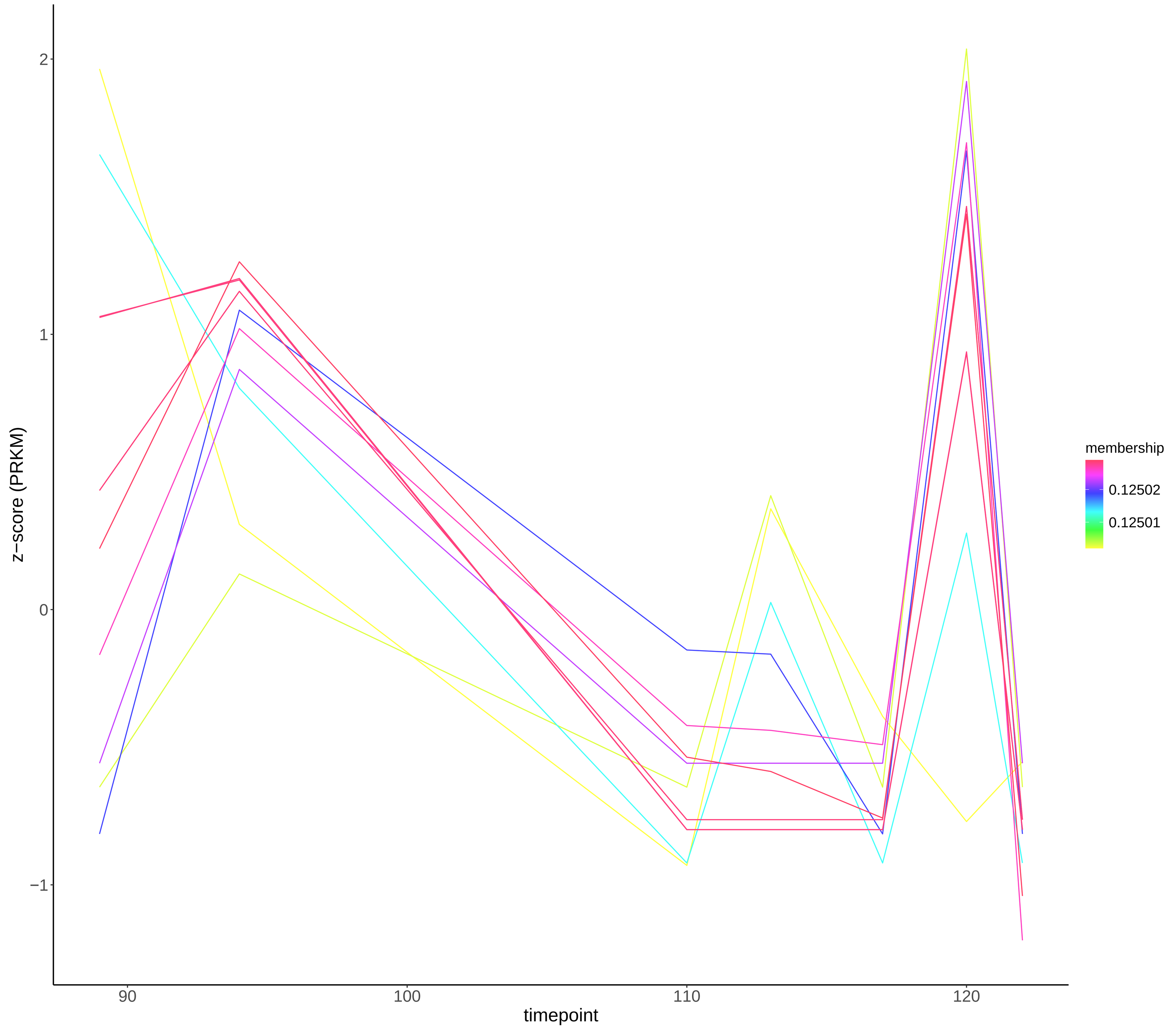

Cluster 7. Number of genes: 5

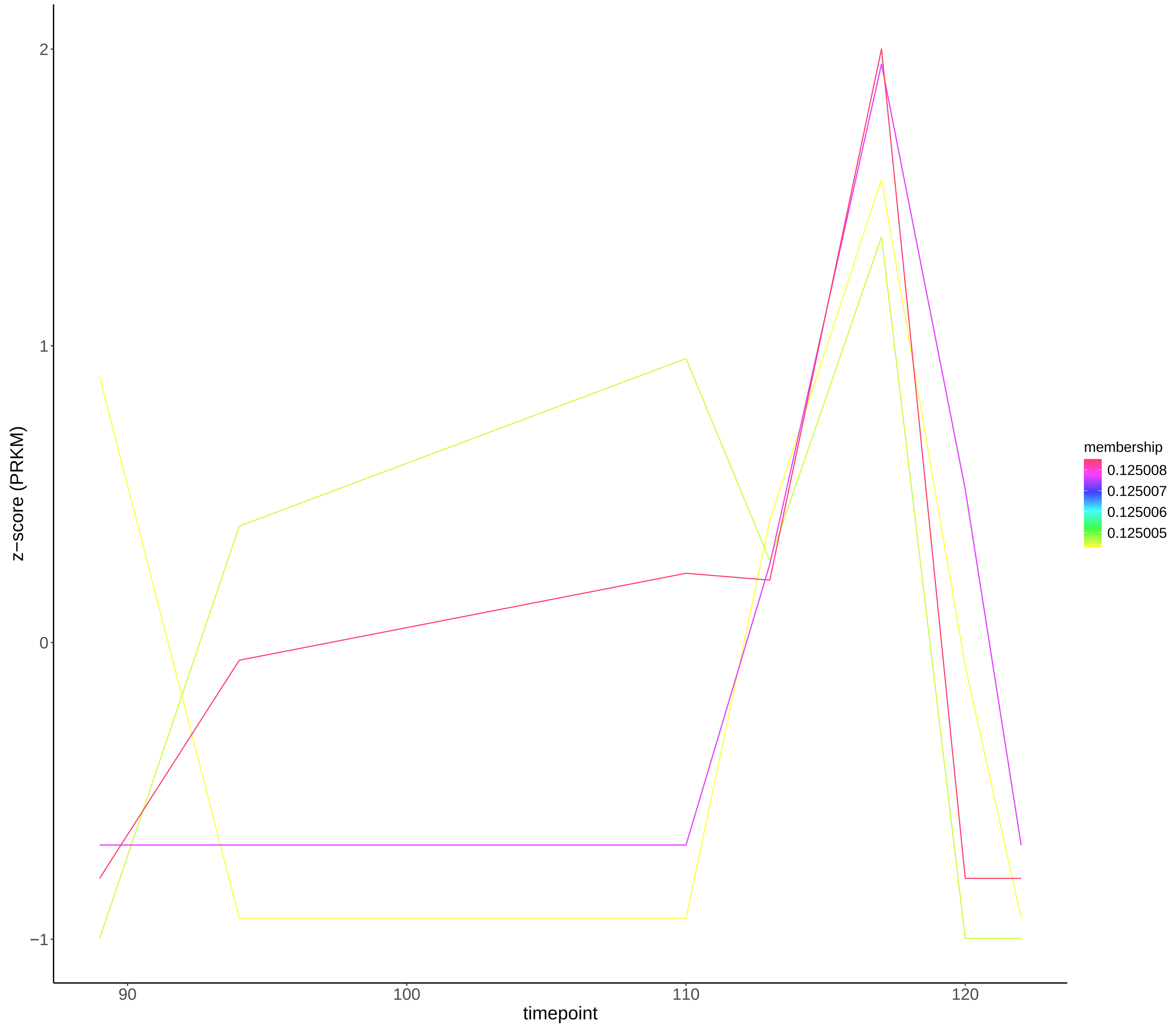

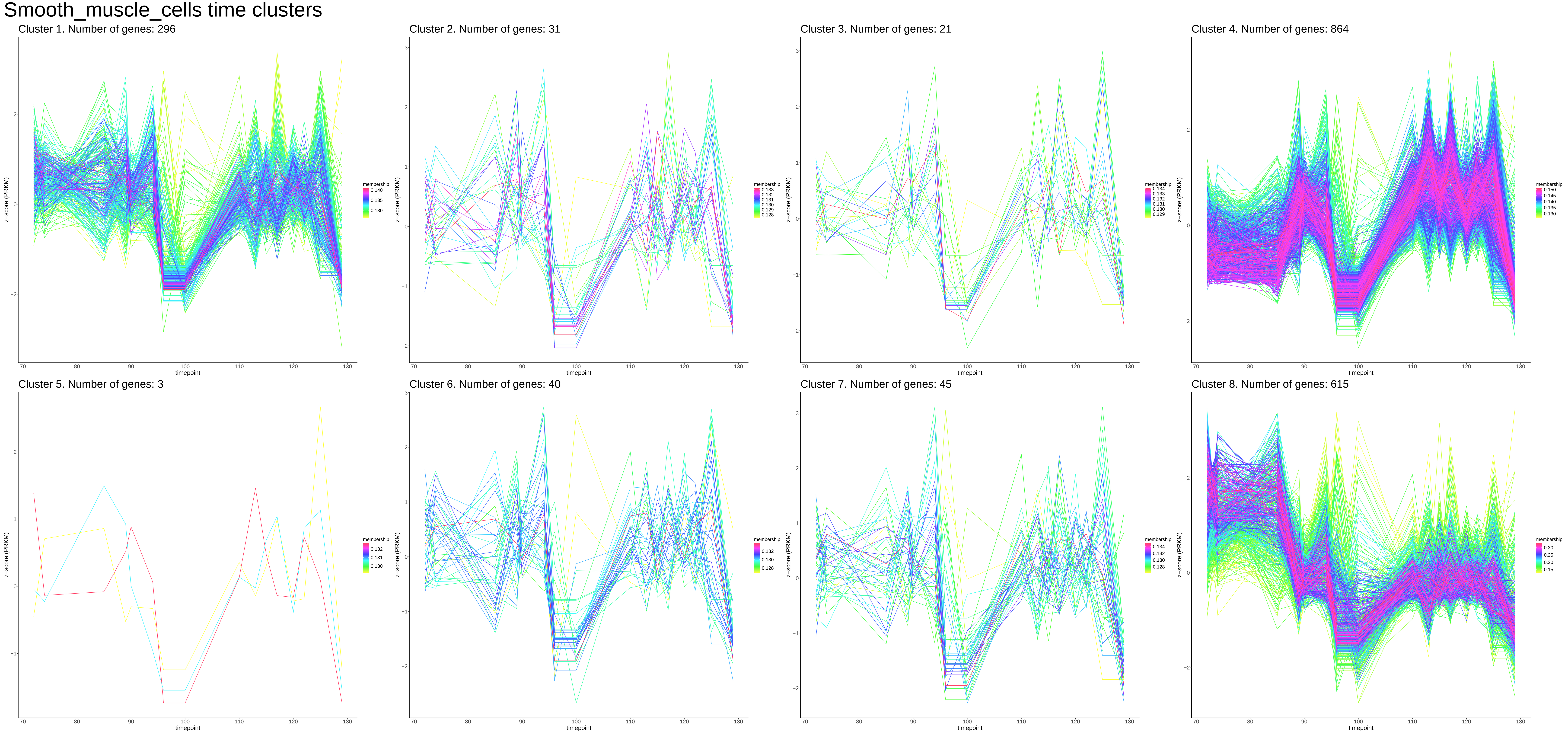
Smooth_muscle_cells time clusters
Cluster 1. Number of genes: 296
Cluster 2. Number of genes: 31
Cluster 3. Number of genes: 21
Cluster 4. Number of genes: 864
Cluster 5. Number of genes: 3
Cluster 6. Number of genes: 40
Cluster 7. Number of genes: 45
Cluster 8. Number of genes: 615
z−score (PRKM)
timepoint
membership

Stellate_cells time clusters

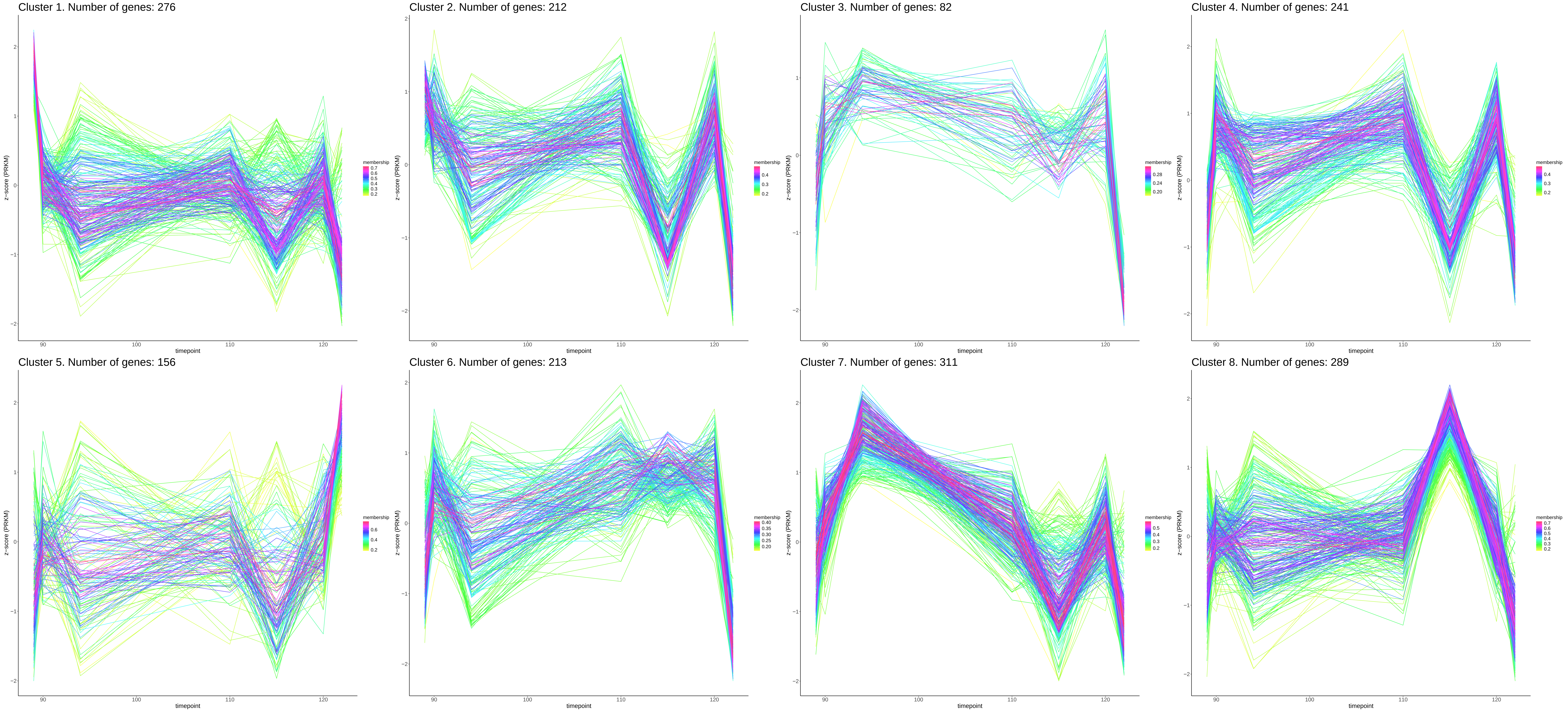

Syncytiotrophoblasts_and_villous_cytotrophoblasts time clusters

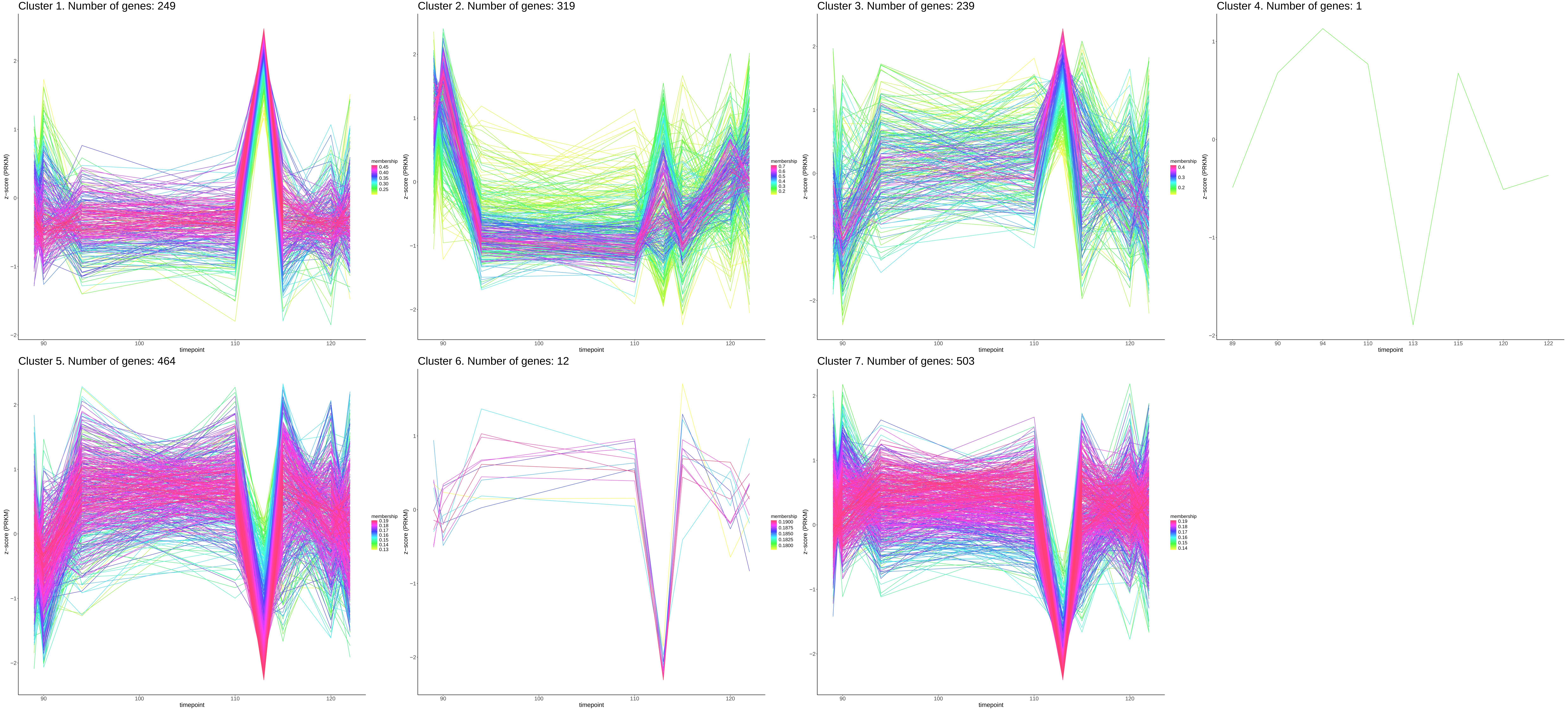

Thymocytes time clusters

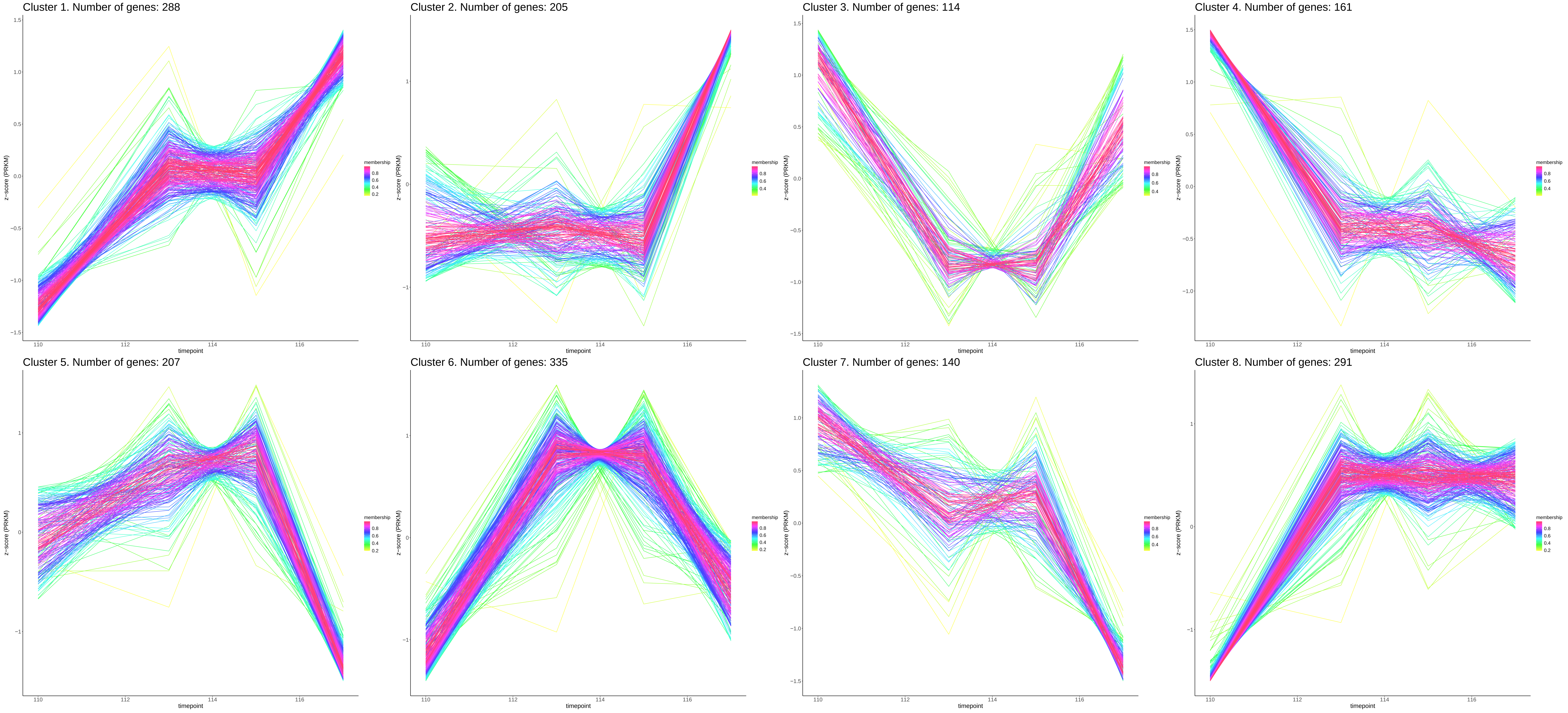

Trophoblast_giant_cells time clusters

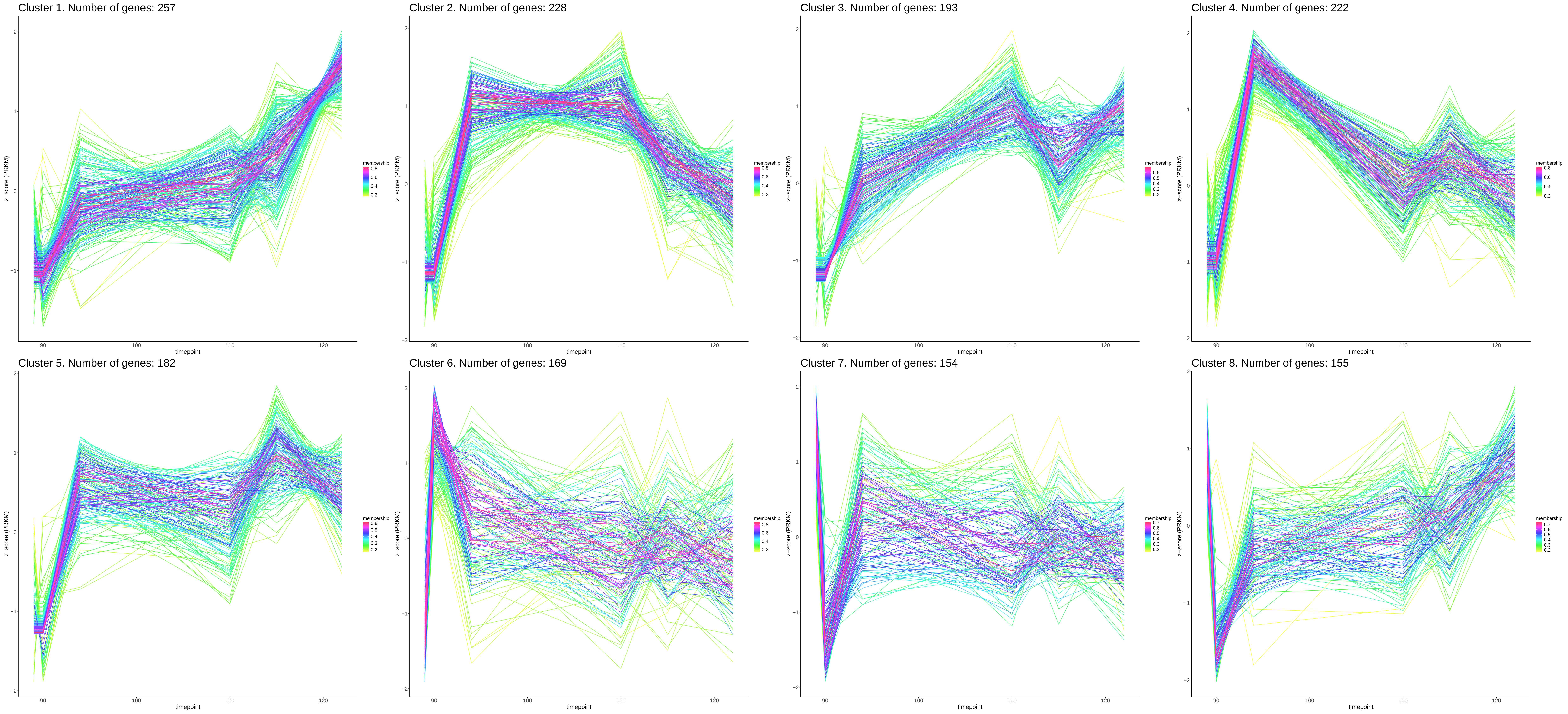

Unipolar_brush_cells time clusters

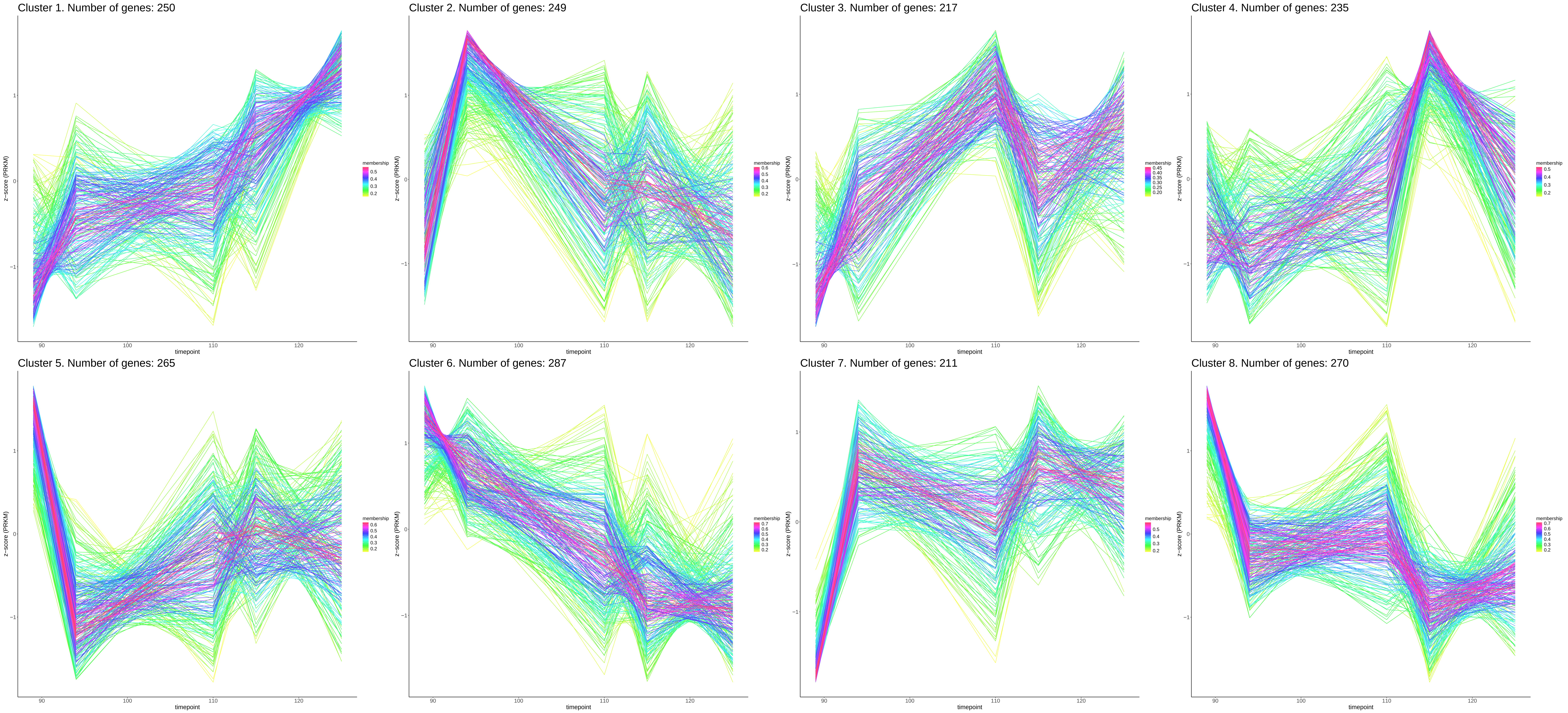

Ureteric_bud_cells time clusters

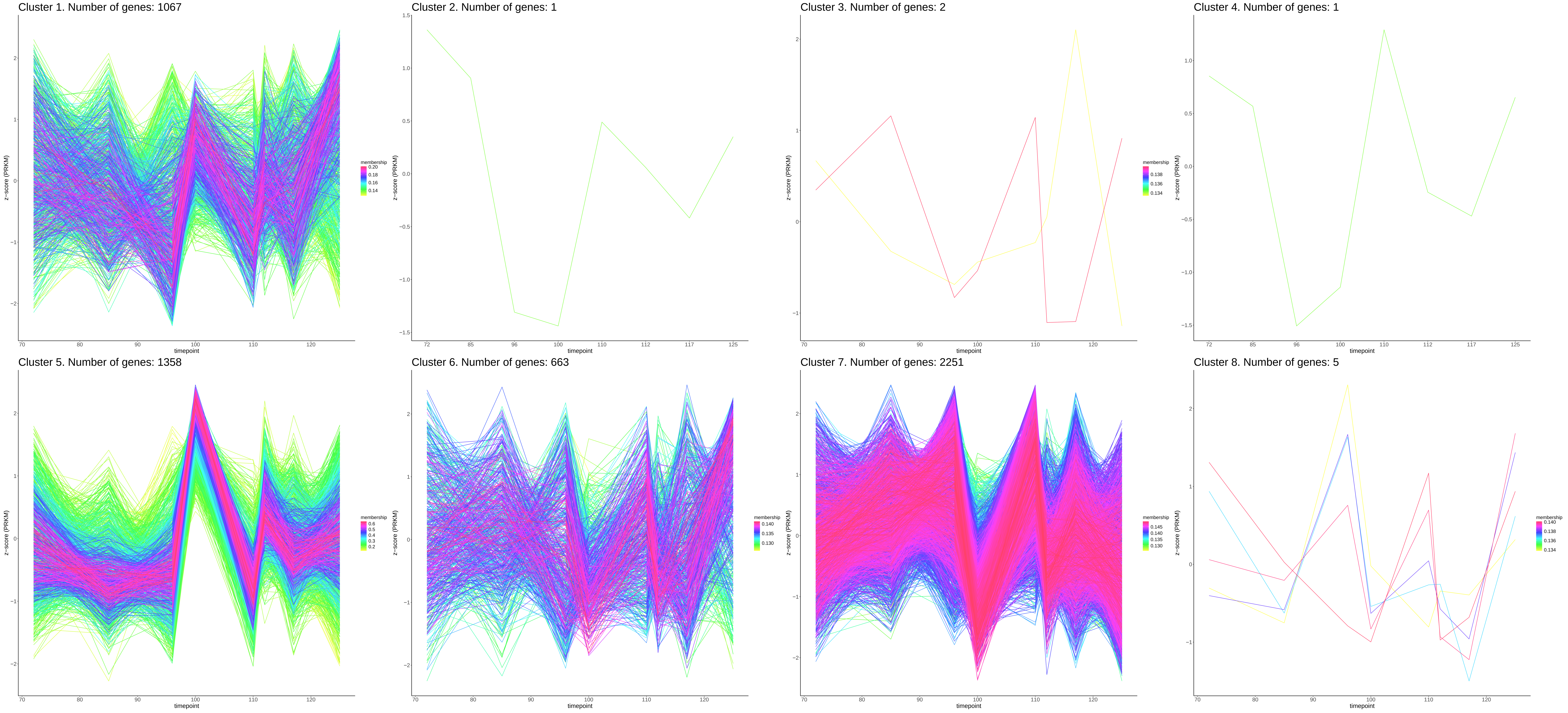

Vascular_endothelial_cells time clusters

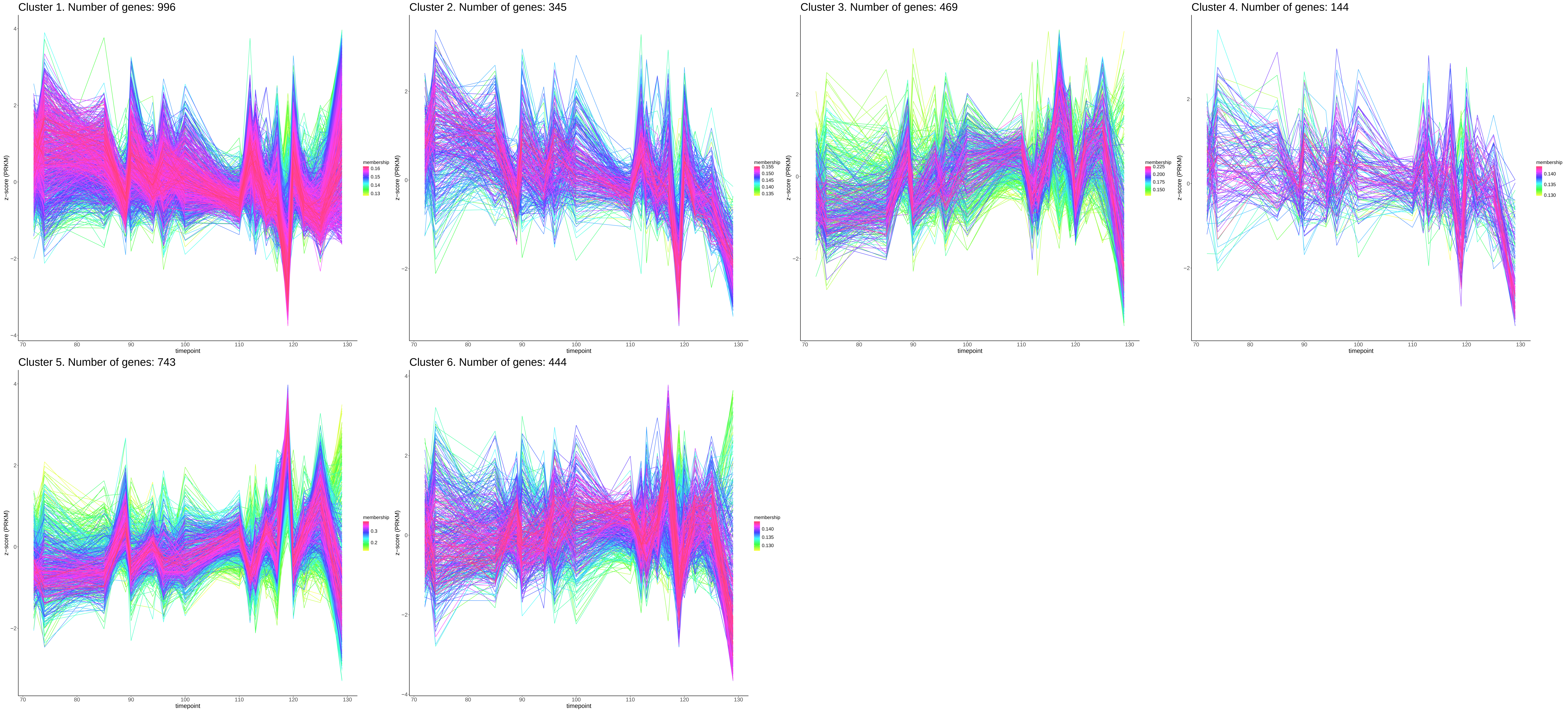

Supplement: lqae180_Supplemental_Files [file FigS1-FetalGeneAtlas_TCseqPlots.pdf › FigS1-FetalGeneAtlas_TCseqPlots.pdf]
